# Spatial fragmentation in the distribution of diatom endosymbionts from the taxonomically clarified dinophyte *Kryptoperidinium triquetrum* (= *Kryptoperidinium foliaceum*, Peridiniales)

**DOI:** 10.1038/s41598-023-32949-y

**Published:** 2023-05-26

**Authors:** Urban Tillmann, Stephan Wietkamp, Juliane Kretschmann, Juliana Chacón, Marc Gottschling

**Affiliations:** 1 Alfred-Wegener-Institute, Helmholtz Centre for Polar and Marine Research, Am Handelshafen 12, 27 570 Bremerhaven, Germany; 2 Department Biologie, Systematics, Biodiversity & Evolution of Plants, GeoBio-Center, Ludwig‐Maximilians-Universität München, Menzinger Str. 67, 80 638 Munich, Germany

**Keywords:** Coevolution, Phylogenetics, Speciation, Taxonomy, Ocean sciences, Biodiversity, Biogeography, Ecosystem ecology

## Abstract

Among the photosynthetically active dinophytes, the Kryptoperidiniaceae are unique in having a diatom as endosymbiont instead of the widely present peridinin chloroplast. Phylogenetically, it is unresolved at present how the endosymbionts are inherited, and the taxonomic identities of two iconic dinophyte names, *Kryptoperidinium foliaceum* and *Kryptoperidinium triquetrum*, are also unclear. Multiple strains were newly established from the type locality in the German Baltic Sea off Wismar and inspected using microscopy as well as molecular sequence diagnostics of both host and endosymbiont. All strains were bi-nucleate, shared the same plate formula (i.e., po, X, 4′, 2a, 7′′, 5c, 7s, 5′′′, 2′′′′) and exhibited a narrow and characteristically L-shaped precingular plate 7′′. Within the molecular phylogeny of Bacillariaceae, endosymbionts were scattered over the tree in a highly polyphyletic pattern, even if they were gained from different strains of a single species, namely *K. triquetrum*. Notably, endosymbionts from the Baltic Sea show molecular sequences distinct from the Atlantic and the Mediterranean Sea, which is the first report of such a spatial fragmentation in a planktonic species of dinophytes. The two names *K. foliaceum* and *K. triquetrum* are taxonomically clarified by epitypification, with *K. triquetrum* having priority over its synonym *K. foliaceum*. Our study underlines the need of stable taxonomy for central questions in evolutionary biology.

## Introduction

Photosynthesis is a fundamental process that essentially shapes the living world as we know it. The origin and establishment of chloroplasts are inferred to have taken place by the close interaction of initially solitary cells during a multi-step evolutionary process^1,2^. The graded series of successive stages comprises spatially regular meeting of partners, recognition and mutual interaction, eventual phagocytosis (or other modes of food uptake), coping with the host’s immune system, intracellular maintenance and interactions, synchronisation of replication and horizontal gene transfer^3–7^. Thus, integration of chloroplast organelles corresponds to the progressive dependence initially of an endosymbiont to a host cell at the structural, physiological, genomic and organisational levels^8,9^.

An endosymbiont retains genes for its own proteins and therefore, its biogenesis does not need to be supported by protein import from the host cell^10,11^. In contrast, an organelle preserves only a small fraction of its original gene set, and all other required genes have been transferred to the host’s nucleus (endosymbiotic gene transfer). The presumably single event of primary endosymbiosis in the Archaeplastida^12,13^ goes back to the Proterozoic eon^14–16^ and has given rise to a highly efficient machinery of carbon fixation as energy source. Secondary and tertiary endosymbiosis events are considered to have taken place multiple times independently^4,9,17,18^. Today, they comprise many different levels of endosymbiont and plastid integration in a taxonomically heterogeneous set of organisms such as euglenids, brown algae, coccoliths, diatoms and dinophytes.

Several phototrophic microorganisms such as the cercozoan *Paulinella*^19,20^ and the ciliate *Mesodinium*^21,22^ have received considerable attention to study the early stages of chloroplast establishment. Moreover, dinophytes are a primary target of research on the origin and establishment of plastids, as they are nothing if not diverse regarding photosynthesis and the involved partners^7,23,24^. Most of the photosynthetically active dinophytes have a peridinin-pigmented plastid deriving from a red algae (based on secondary endosymbiosis), which has been replaced by other types of plastids in some lineages. They include *Lepidodinium* with an independent secondary endosymbiont of pedinophyte origin^25^, Brachydiniaceae with fucoxanthin-pigmented plastids as a result of tertiary endosymbiosis^26,27^ and some gymnodinioid dinophytes performing kleptoplastidy^28,29^.

Another exceptional group of dinophytes are the Kryptoperidiniaceae hosting a tertiary endosymbiont derived from a diatom^7,30–32^. They have a unique and morphologically conserved type of an eyespot^33,34^ that has possibly been derived from the original peridinin chloroplast^23,35–37^. An almost intact diatom ultrastructure with insignificant genome reduction^38,39^ (except the total loss of the frustule), and absence of co-phylogeny between hosts and endosymbionts^40,41^, are supportive for repeated and geologically young, if not recent events of diatom capture. The evolutionary scenario is also corroborated by existence of kleptoplasty in *Durinskia capensis*^42^.

Kryptoperidiniaceae comprise some 20 species of *Blixaea*, *Dinothrix*, *Durinskia*, *Kryptoperidinium* and *Unruhdinium* occurring in both marine and freshwater environments^32,43–45^. They belong to the Peridiniales and may form a group having five cingular plates (*versus* six plates predominant among peridinialean dinophytes) together with Blastodiniaceae, Ensiculiferaceae and Zooxanthellaceae^46,47^, but statistical support for the group in molecular phylogenetics is still low. Within that group, Kryptoperidiniaceae are the only dinophytes encountering not more than two intercalary plates (*versus* three such plates in many peridinialean remainders), which might be apomorphic. There are no fossils known of Kryptoperidiniaceae, but origin and early diversification have been dated to the Cretaceous^41,48,49^, indicating a relatively old age of the group. This estimated age much exceeds the oldest diatom fossils that are considered relatives of the extant endosymbionts^50,51^.

Among marine dinotoms, *Kryptoperidinium* is the best studied and most widespread group and forms dense blooms in coastal areas worldwide^43,52–54^. It has been reported from the Baltic Sea, the Mediterranean Sea, the Black Sea, the Caspian Sea, the North Sea, the Atlantic Ocean, the Indian Ocean (with the Persian Gulf) and the Pacific Ocean, including also the seas around Australia^52,54–56^. The algae have been investigated in detail regarding life history^53,57^, behaviour^58^, ultrastructure^23,35,57^, compounds^59^ and pigment profiles^52,60^.

*Kryptoperidinium* is currently considered monotypic, but there are deviating reports of the thecal plate formula either consisting of three^53^ or four apical plates^43,52,54^, and the number of cingular plates (i.e., four or five) is also unclear. Moreover, the species’ name was confused in the past^61^. For a long time, the name *Kryptoperidinium foliaceum*^55,62^ was applied, but this is now considered a younger heterotypic synonym of *Kryptoperidinium triquetrum*^63,64^. Notably, both taxa have been described from the Baltic Sea off Wismar, but their taxonomic identity remains ambiguous until newly collected material from the type locality has been inspected using the battery of contemporary techniques (i.e., the crucial issue of the present study). *Kryptoperidinium* hosts various bacillariacean diatoms as endosymbiont, mostly related to free-living species of *Nitzschia*^40,41,65^.

In this study, the taxonomic identities of two iconic names of dinophytes, *K. foliaceum* and *K. triquetrum*, are clarified, and the thecal plate pattern of the species is inferred from multiple strains from different geographic origins. Comprehensive rRNA sequences are provided not only of the hosts, but also of the endosymbionts. The endosymbiont sequences are embedded in a data matrix using concatenated sequences of diatoms^66^. The absence of co-phylogeny between the endosymbionts and their hosts is shown, favouring an evolutionary scenario of ongoing, repeated though group-specific uptake of diatoms. A single species of dinotoms may harbour a diversity of endosymbionts showing biogeographic correlations, and our results may stimulate functional research on rise and establishment of chloroplasts in general.

## Results

### Morphology in light microscopy

All strains of *Kryptoperidinium* studied here (Table 1) were morphologically indistinguishable. For taxonomic clarification, one strain from each of the two sampling sites in Wismar was selected. Specifically, 1 of the 7 strains from Wendorf pier off Wismar (W1-E4, deposited at the Central Collection of Algal Cultures, CCAC 9297B) was selected for epitypification of *K. foliaceum* and 1 of the 7 strains from Wismar marina (W4-A6, deposited at the Central Collection of Algal Cultures, CCAC 9296B) for epitypification of *K. triquetrum*. Strain W4-A6 will be described and depicted in detail, and respective micrographs of other selected strains (including strains from Finland and Spain) are presented in the Supplementary information (Figs. S1‒S17, Table S1).

**Table 1:** *Kryptoperidinium triquetrum* strains inspected in the course of the study.

Strain	Origin	Isolator	Date	LM	Figure plate
G-E8	Greifswald	U. Tillmann	2019	x	Figure S9
G-E10	Greifswald	U. Tillmann	2019	x	
G-F9	Greifswald	U. Tillmann	2019	x	Figure S10
G-F11	Greifswald	U. Tillmann	2019	x	
GeoB 459	Mediterranean, Aegean Sea	M. Kirsch	2010	x	Figure S17
KFF 0901	Föglö, Aland	A. Kremp	2009	x	Figures S11 and S12
KFF 1001	Föglö, Aland	P. Hakanen	2010	x	Figures S13 and S14
VGO 556	Ulla estuary, Ria de Aurosa	n.a	2002	x	Figure S15
VGO 1124	Baiona, Rio da Vigo	n.a	n.a.	x	Figure S16
W1-C6	Wismar Wendorf pier	U. Tillmann	2019	x	
W1-C7	Wismar Wendorf pier	U. Tillmann	2019	x	
W1-D1	Wismar Wendorf pier	U. Tillmann	2019	x	
W1-D6	Wismar Wendorf pier	U. Tillmann	2019	x	Figure S6
W1-D11	Wismar Wendorf pier	U. Tillmann	2019	x	Figures S7 and S8
**W1-E4**	**Wismar Wendorf pier**	**U. Tillmann**	**2019**	**x**	Figures S4 and S5
W1-E12	Wismar Wendorf pier	U. Tillmann	2019	x	
**W4-A6**	**Wismar marina**	**U. Tillmann**	**2019**	**x**	Figures 1, 2, 3, 4, 5, 6 and 7 (Supplementary Figures 1–7)
W4-A7	Wismar marina	U. Tillmann	2019	x	
W4-A9	Wismar marina	U. Tillmann	2019	x	
W4-A10	Wismar marina	U. Tillmann	2019	x	Figure S1
W4-B10	Wismar marina	U. Tillmann	2019	x	
W4-F1	Wismar marina	U. Tillmann	2019	x	
W4-F9	Wismar marina	U. Tillmann	2019	x	Figures S2 and S3
W20-H6	Wismar marina	U. Tillmann	2020	–	
W20-H7	Wismar marina	U. Tillmann	2020	–	

Bold: strains used for epitypification. Strains KFF 0901 and KFF 1001 were obtained from the FINMARI Culture collection/SYKE Marine Research Centre and Tvärminne Zoological Station (FINMARI CC). Strains VGO 556 and VGO 1124 were obtained from the VGOHAB culture collection of Vigo (Spain).

**Figure 1:**
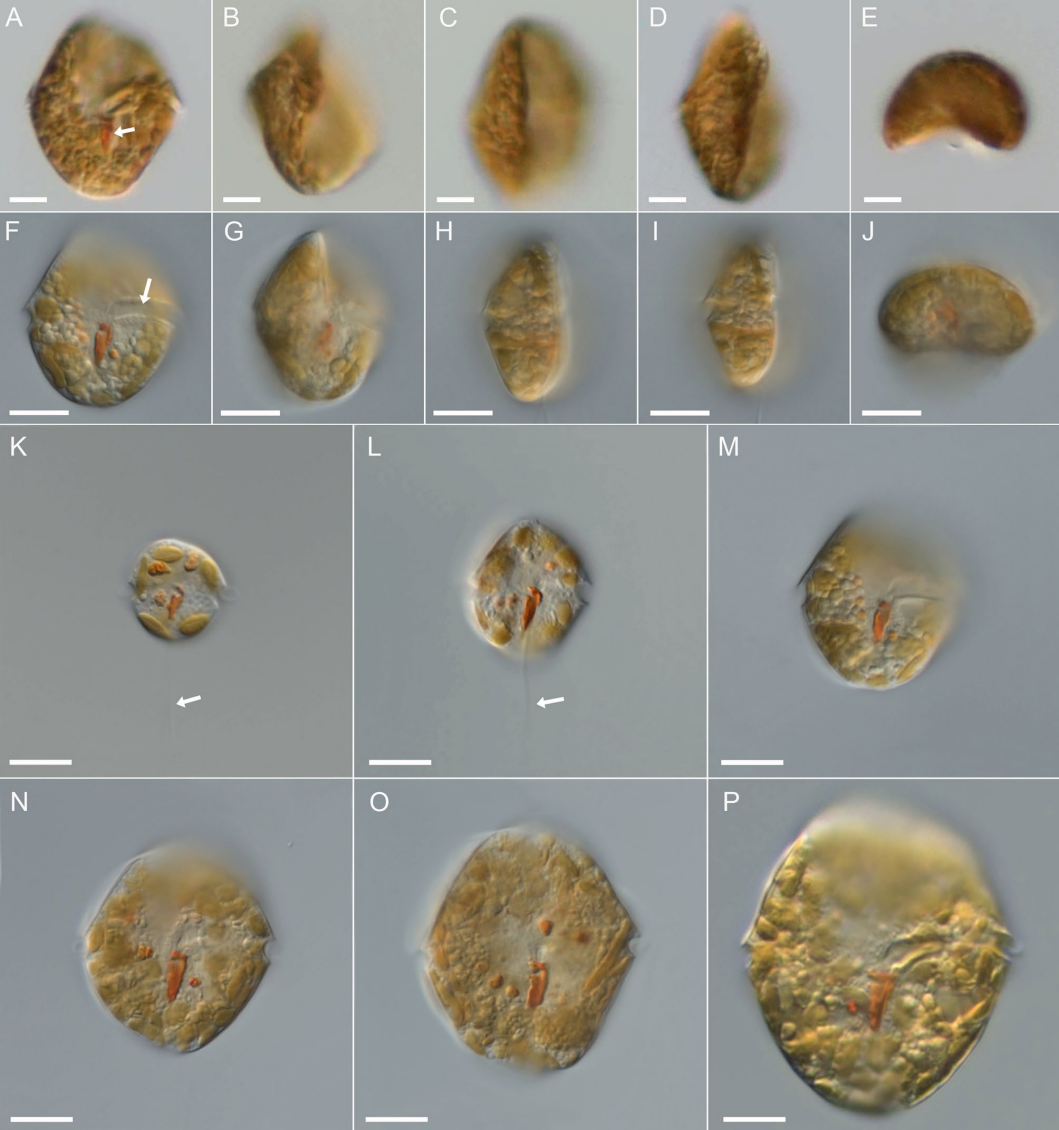
*Kryptoperidinium triquetrum*, strain W4-A6. LM of living cells (**A‒P**). (**A‒E**) The same cell in ventral (**A**), ventral lateral (**B,C**), lateral (**D**) and antapical view (**E**). (**F‒J**) Another cell in ventral (**F**), ventral lateral (**G**), lateral (**H,I**) and antapical view (**J**). (**K‒P**) Cells of different size in ventral view; note the red stigma (white arrow in (**A**)), the cingular groove (white arrow in (**F**)) and the longitudinal flagellum (white arrows in (**K,L**)). Scale bars = 10 µm.

**Figure 2:**
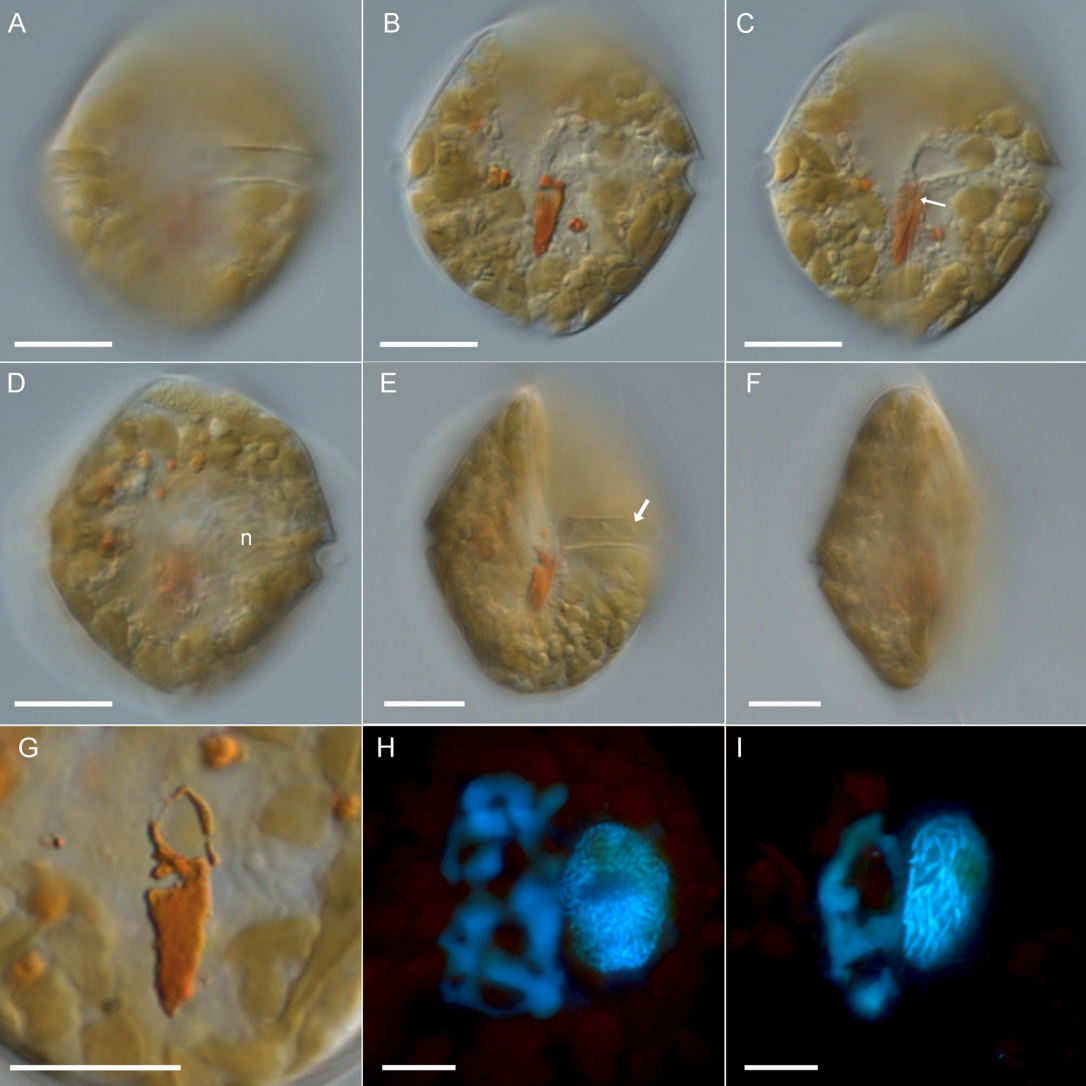
*Kryptoperidinium triquetrum*, strain W4-A6. LM of living cells (**A‒G**) or formaldehyde-fixed cells (**H,I**). (**A‒D**) The same cell in ventral view in different focal planes. Note the red stigma in (**B,C**), the sulcal funnel (white arrow in (**C**)) and the dinophyte nucleus (n) in (**D**). (**E**) Cell in ventral lateral view; note the wavy transverse flagellum in the cingulum (white arrow). (**F**) Cell in lateral view. (**G**) Detailed view of the stigma; note that the cell was squeezed causing a slight deformation of the anterior hook-shaped projection. (**H,I**) Different cells stained with DAPI and viewed with epifluorescence and UV excitation; note the irregularly shaped diatom nucleus (left) and the dinophyte nucleus with condensed chromosomes (right). Scale bars = 10 µm.

Motile cells were predominant (Figs. 1, 2) and had a transverse flagellum (Fig. 2E) and a longitudinal flagellum, which was approximately as long as the cell (Fig. 1K,L). Cells swam with rapid turns in narrow helical paths towards light that in culture flasks under microscopic illumination, they usually gathered dense aggregates on the side facing the light (Video SV01). Exponentially growing cells had intense, orange-brown colour (Figs. 1, 2). Motile cells varied greatly in size, and cell length ranged continuously from 15 to 50 µm (Fig. 1K‒P).

Motile cells were longer than wide, with length/width ratios of about 1.1. In dorsal view, they were slightly variable in outline with an asymmetrically rounded through acute episome and a more symmetric and rounded hyposome (Figs. 1N–P, 2A–D). They had a strong dorso-ventral compression, with convex dorsal and concave ventral surfaces (Figs. 1B–D,G–I, 2E,F). The left and right lateral sides were slightly angled around the longitudinal axis that in lateral view, the cells had a triangular outline, with the width comprising about 40% of the cell length (Figs. 1D,H,I, 2F). The cingulum was narrow (ca. 3 µm in height), excavated and almost median or slightly sub-median in position (Fig. 1F), that the episome—if at all—was only slightly larger than the hyposome. In counter-clockwise direction, the cingular groove ended well before its start (Figs. 1F, 2A) and from many observations of living cells, no displacement was identified. In the sulcal area just below the cingulum, a narrow and covered funnel (arrow in Fig. 2C) for the longitudinal flagellum was present.

A large number of ovoid or elongated, small chloroplasts (ca. 3‒5 µm in length) were present in a peripheral position (Fig. 2A‒E). Light microscopic observation of living cells revealed a large, ovoid dinokaryon located on the cell’s left lateral side in the cingular plane (Fig. 2D), which was frequently difficult to observe because of the obscuring, densely packed chloroplasts. Nuclear staining using DAPI (Fig. 2H,I) clearly showed the presence of two nuclei, namely the large dinophyte nucleus with condensed chromosomes and the endosymbiont nucleus. The latter was highly irregular in shape, more faintly stained, and no chromosomes were discernible. In the central sulcal area just below the cingulum, there was a conspicuous eyespot of intense, red colour (Figs. 1A,F,K–P, 2B,G). The eyespot extended into the hyposome and had a characteristic, rectangular or trapezoid shape with a slightly pointed posterior part and a hook-shaped anterior projection.

**Figure 3:**
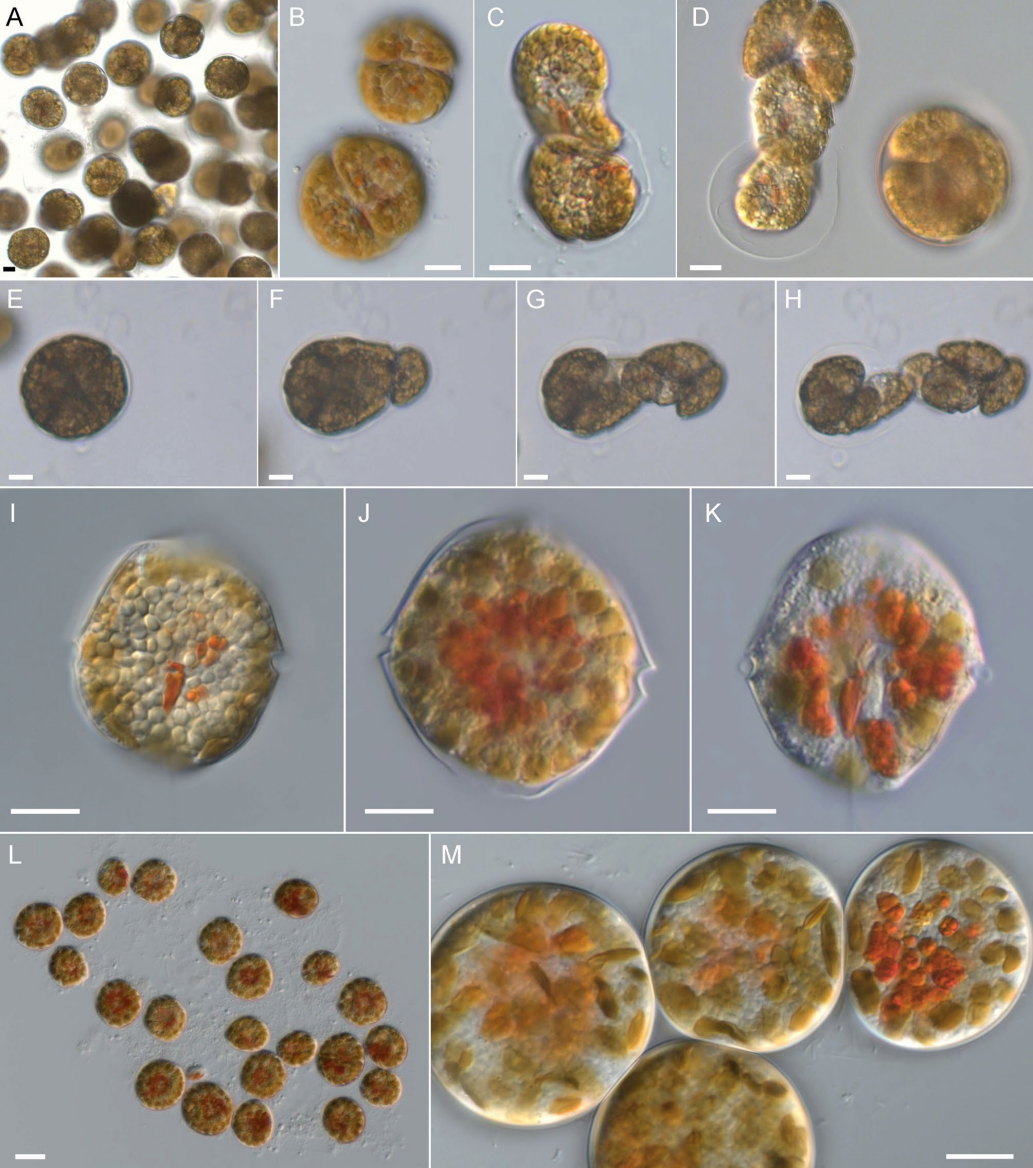
*Kryptoperidinium triquetrum*, strain W4-A6. LM of living cells (**A‒M**). (**A**) Coccoid division stages accumulated at the culture flask bottom. (**B**) Two sporocysts in two-celled (above) or four-celled stage (below). (**C**) Two-celled sporocyst during hatching. (**D**) Four-celled sporocyst (right) and four-celled sporocyst during hatching (left; note the hyaline coat). (**E‒H**) Single frames of an eight-celled sporocyst during hatching. (**I‒K**) Motile cells in stationary phase. (**I**) Cell densely filled with small starch grains. (**J,K**) Cells accumulating reddish globules and with reduced chloroplasts. (**L,M**) Coccoid cells accumulating at the culture flask bottom during stationary phase. Scale bars = 10 µm.

In growing strains, dividing and pre-division cells were easily distinguishable as non-motile and spherical coccoid stages on the bottom of the cultivation vessels (Fig. 3A,B). From these division stages, 2 or 4 daughter cells emerged and left behind a thin, hyaline coat (Fig. 3C,D). On a number of occasions, the formation of 8 daughter cells was also observed (Fig. 3E‒H, Video SV01). In stationary phase, the number of chloroplasts was reduced, and cells were often densely filled with small starch grains (Fig. 3I). In addition, cells in stationary growth phase accumulated numerous reddish globules in their centre (Fig. 3J,K) and eventually formed large clusters of coccoid cells, with no indication of further cell division (Fig. 3L,M).

### Thecal plate pattern

**Figure 4:**
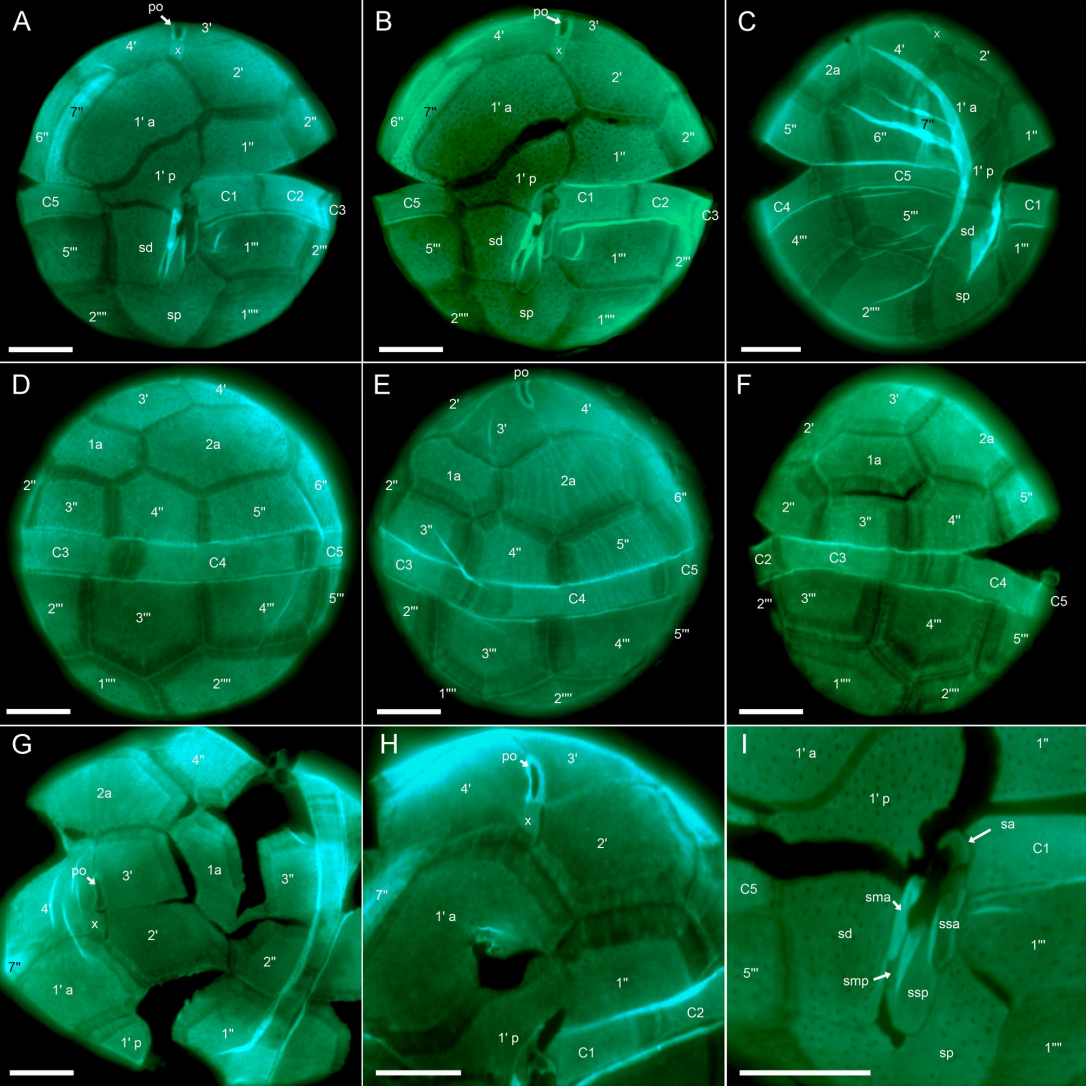
*Kryptoperidinium triquetrum*, strain W4-A6. LM of lugol-fixed cells stained with solophenyl flavine and viewed with epifluorescence and green light excitation. (**A,B**) Cells in ventral view. (**C**) Cell in ventral right-lateral view. (**D‒F**) Cells in dorsal view. (**G,H**) Detailed view of epithecal plates in apical view. (**I**) Detailed view of the sulcal area with sulcal plates. Plate labels according to the Kofoidean system, modified by labelling an anterior part (1′ a) and a posterior part (1′ p) of the first apical plate. Sulcal plate labels: *sa* anterior sulcal plate; *sd* right sulcal plate, *sma* anterior median sulcal plate, *smp* posterior median sulcal plate, *sp* posterior sulcal plate, *ssa* anterior left sulcal plate, *ssp* posterior left sulcal plate. Scale bars = 10 µm.

**Figure 5:**
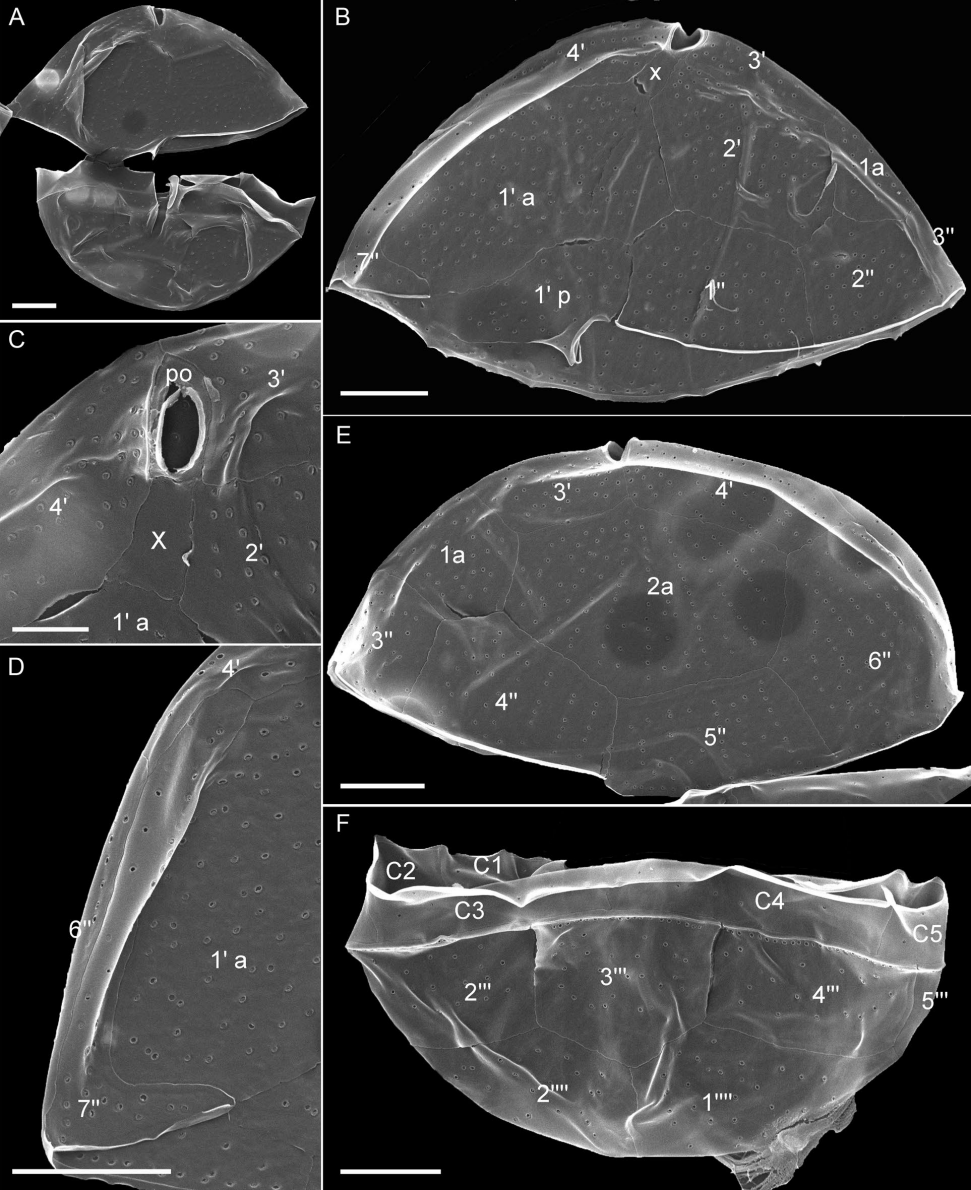
*Kryptoperidinium triquetrum*, strain W4-A6. SEM of thecate cells. (**A**) Cell in ventral view. (**B**) Epitheca in ventral view. (**C**) Detailed view of the apical pore complex and apical plates. (**D**) Detailed view of the narrow last precingular plate 7′′. (**E**) Epitheca in dorsal view. (**F**) Dorsal view of hypothecal and cingular plates. Plate labels according to the Kofoidean system, modified by labelling an anterior part (1′ a) and a posterior part (1′ p) of the first apical plate. Scale bars = 5 µm (**A,B,D‒F**) or 2 µm (**C**).

**Figure 6:**
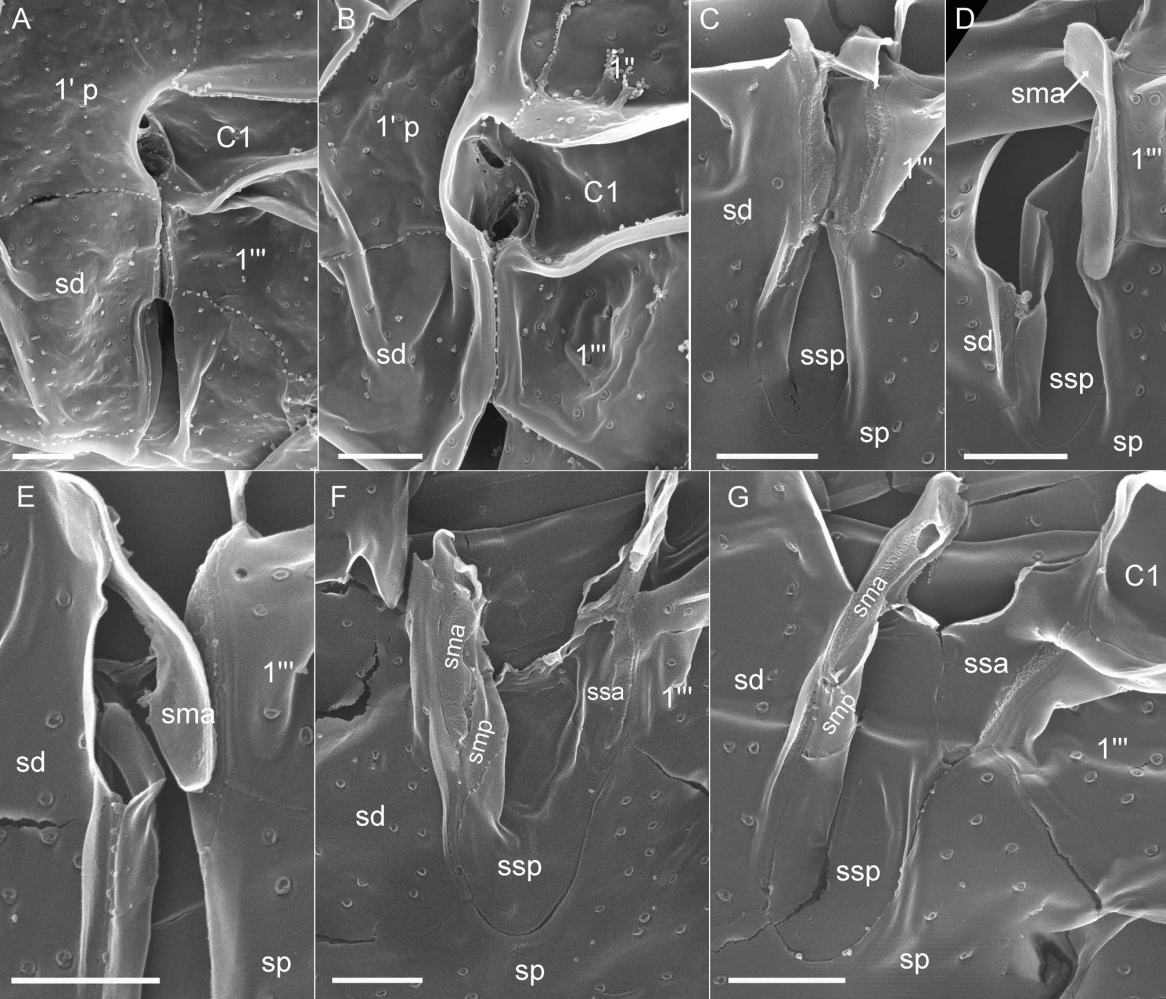
*Kryptoperidinium triquetrum*, strain W4-A6. SEM of thecate cells. (**A‒G**) Detailed view of the sulcal area. Plate labels according to the Kofoidean system, modified by labelling an anterior part (1′ a) and a posterior part (1′ p) of the first apical plate. Sulcal plate labels: *sd* right sulcal plate, *sma* anterior median sulcal plate, *smp* posterior median sulcal plate, *sp* posterior sulcal plate, *ssa* anterior left sulcal plate, *ssp* posterior left sulcal plate. Scale bars = 2 µm.

**Figure 7:**
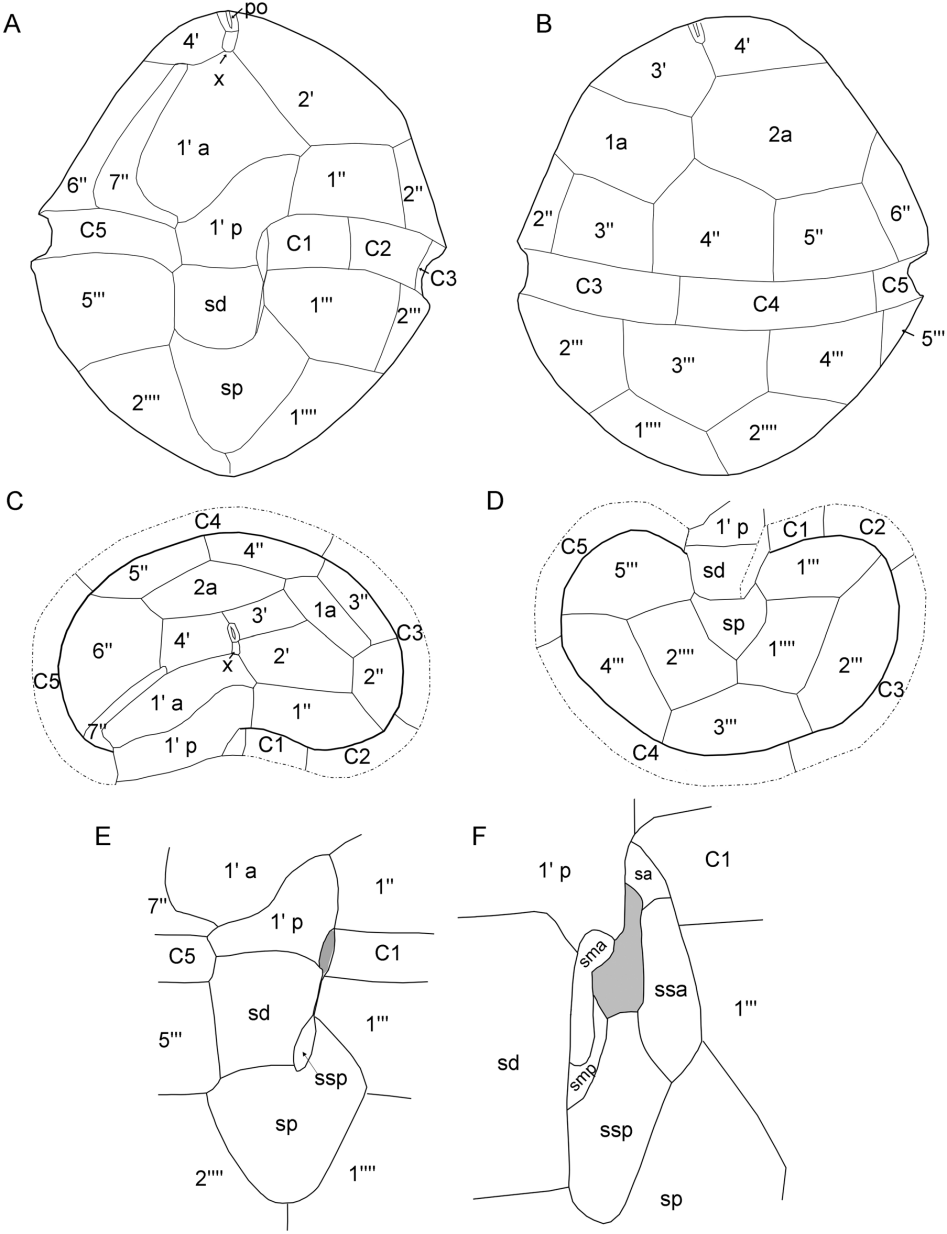
Schematic line drawings of *Kryptoperidinium triquetrum* plate pattern. (**A**) Ventral view. (**B**) Dorsal view. (**C**) Epithecal plates in apical view. (**D**) Hypothecal plates in antapical view. (**E,F**) Sulcal plates in undisturbed conformation (**E**) and detailed view on the small central sulcal plates, when the flagellar canal is artificially open (**F**).

The theca was faintly visible in living cells (Figs. 1, 2), but the plate pattern could be elucidated with epifluorescence microscopy after cellulose staining (Fig. 4). It was confirmed and supplemented by SEM analyses (Figs. 5, 6). Thecal plates were smooth but densely ornamented with small pores, which were mostly scattered over the ventral plates (Fig. 4A,B) though often distinctly arranged in rows on some dorsal plates of the epitheca (Fig. 4E). The plate pattern was identified as po, X, 4′, 2a, 7′′, 5C, 7S, 5′′′, 2′′′′ and is schematically drawn in Fig. 7.

At the apex of the epitheca, there was a slender and elongated pore plate with a slender apical pore opening (Figs. 4A,B,E,H, 5A–C,E). Ventrally to the pore plate, a small X-plate (canal plate) was present, which was rectangular and longer than wide (Figs. 4A,B,G,H, 5B,C). Posteriorly to the X-plate, there was a big plate covering the largest part of the left ventral epitheca and having a characteristic curvature towards its anteriorly adjacent plate. Both plates corresponded to plate 1′, which was subdivided into an anterior part (here labelled as 1′ a) and a posterior part (here labelled as 1′ p). Plate 1′ a abutted the X-plate but not the pore plate. Plate 1′ p was in contact with both terminal cingular plates C1 and C5 and both terminal precingular plates 1′′ and 7′′ (Fig. 4A,I). Plate 2′ was located on the ventral side and had a very narrow joint suture with the pore plate (Figs. 4H, 5B,C). Plate 3′ was the smallest apical plate, and plate 4′ was located on the right-lateral side of the cell. The two anterior intercalary plates had a dorsal position and abutted six other epithecal plates. Plate 2a was mid-dorsal in position and slightly larger than plate 1a (Figs. 4D,E, 5E). Within the precingular plates series, plates 1′′ through 5′′ were of similar height, but the right lateral plate 6′′ was higher than the others (Fig. 4A,B,D,E). Plate 7′′ was conspicuously L- or boot-shaped with a narrow upper part and a broader base abutting C5. This plate always appeared very bright under fluorescent light of stained samples (Fig. 4A‒C), but SEM revealed no obvious difference in plate thickness or surface structure (Fig. 5B,D).

The cingular groove was discontinuous and disconnected ventrally by plate 1′ p (Fig. 4A,B,I). Plates C1 and C2 were of similar size and smaller than the remaining cingular plates (Fig. 4A‒F). The suture between plates C2 and C3 was in lateral position and thus often difficult to observe. In the hypotheca (Fig. 4A‒F), plate 3′′′ was in dorsal position and abutted both antapical plates (Fig. 4D), which were of comparable size (Fig. 4A,B,D). The sulcal area was dominated by two large plates, the right and posterior sulcal plates sd and sp, respectively (Fig. 4A,B). Plate sd was roughly rectangular and abutted posteriorly the right side of the large and asymmetric plate sp. The left anterior side of plate sp was triangular and shared a broad suture with plate 1′′′ (Fig. 4A,B). The small plates in the central sulcal area were difficult to observe by LM, but two tongue-shaped plates (a posterior left sulcal plate: ssp and an anterior left sulcal plate: ssa) were clearly visible. Anteriorly to plate ssa, there was a small and posteriorly curved anterior sulcal plate sa contacting plates C1 and 1′ p (Fig. 4I). On the left side of the large right sulcal plate sd, there was an elongated anterior median sulcal plate sma, which always was brightly stained (Fig. 4A,B,I).

Using SEM (Figs. 5, 6), thecal pore size was estimated as 0.15‒0.20 µm in diameter. A few plates were consistently free of pores, namely the pore plate, the X-plate (Fig. 5C) and all small central sulcal plates (Fig. 6). There was a dense row of pores on postcingular plates below the cingulum with its five cingular plates (Fig. 5F). Moreover, SEM enabled detailed observations of number and arrangement of the small plates in the central sulcus (Fig. 6). In a presumably undisturbed arrangement, plates sd and 1′′′ were in close proximity posteriorly to the flagellar pore region and formed a narrow, closed canal for the longitudinal flagellum (arrow in Figs. 2C, 6A,B). Various SEM views of artificially opened sulcal areas (Fig. 6C‒G) indicated that in fact, this connection of plates sd and 1′′′ was made by two inward-bound, small plates, namely by an anterior median sulcal plate sma on the cell’s right side and an anterior left sulcal plate ssa on the cell’s left side. Both plates had a partly rough surface, as if both plates had been glued together. Plate sma (on the cell’s right side of the sulcal groove) had a characteristically spoon-like shape (Fig. 6D,E,G). Exceptionally, this plate was artificially separated from plate sd and was seen on the left side still closely attached to plate ssa (Fig. 6D). In the central sulcal area, the larger and tongue-like posterior left sulcal plate ssp was visible. Between plates ssp and sma, there was another small and narrow sulcal plate, namely the posterior median sulcal plate smp, which was not clearly visible in LM (Fig. 4I). On the other hand, the small anterior sulcal plate sa (anteriorly of the flagellar pore area) was clearly visible in LM (Fig. 4A,B,I) but was lost or could not be clearly observed in SEM preparations (Fig. 6A,B).

### Molecular phylogenetics

**Figure 8:**
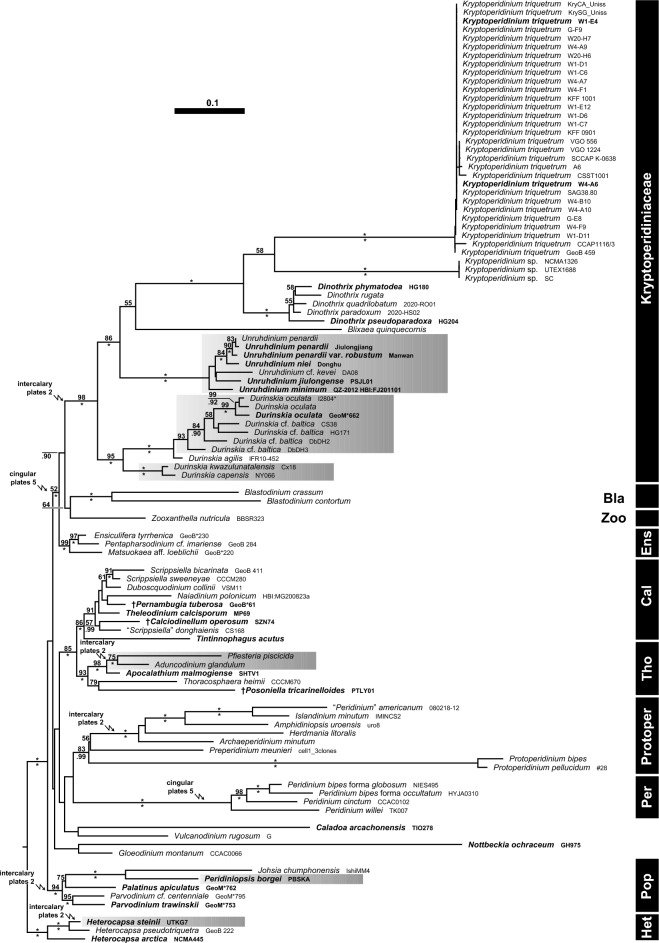
A molecular reference tree recognising major groups of Peridiniales (created using Adobe Illustrator^©^ CS6; https://www.adobe.com/de/products/illustrator.html). Maximum Likelihood (ML) tree of 101 systematically representative peridinialean sequences with a focus on Kryptoperidiniaceae (with strain number information) as inferred from a rRNA nucleotide alignment (1712 parsimony-informative positions). Numbers on branches are ML bootstrap (above) and Bayesian probabilities (below) for the clusters (asterisks indicate maximal support values, values under 50 and .90, respectively, are not shown). Dinophytes exhibiting 6 (instead of 7) precingular plates are highlighted by grey boxes. Evolutionary transformations from six to five cingular plates, and from three to two intercalary plates, are indicated by flash symbols. *Bla* Blastodiniaceae, *Cal* ^†^Calciodinelloideae, *Ens* Ensiculiferaceae, *Het* Heterocapsaceae, *Per* Peridiniaceae, *Pop* Peridiniopsidaceae, *Protoper* Protoperidiniaceae, *Tho* Thoracosphaeroideae, *Zoo* Zooxanthellaceae.

The SSU + ITS + LSU alignment of dinophytes was 1821 + 828 + 2998 bp long and was composed of 457 + 539 + 716 parsimony-informative sites (30%, mean of 16.78 per terminal taxon) and 2758 distinct RAxML alignment patterns. Figure 8 (Supplementary Figure 8) shows the best-scoring ML tree (− ln = 52,477.03), with the majority of nodes showing high if not maximal support. The Kryptoperidiniaceae were monophyletic (98LBS, 1.00BPP) and comprised *Durinskia* (95LBS, 1.00BPP), *Blixaea* (single accession), *Unruhdinium* (100LBS, 1.00BPP), *Dinothrix* (100LBS, 1.00BPP) and *Kryptoperidinium* (58LBS). The latter segregated into two clades, namely *Kryptoperidinium* I containing all strains of the present study and assigned to *K. triquetrum* (100LBS, 1.00BPP) and *Kryptoperidinium* II (100LBS, 1.00BPP; determined as *Kryptoperidinium* sp.). Within *Kryptoperidinium* I, ITS sequence variability was low, but the VGO-strains differed from the other available sequences in four ITS positions (plus two positions in the hypervariable region of the LSU).

**Figure 9:**
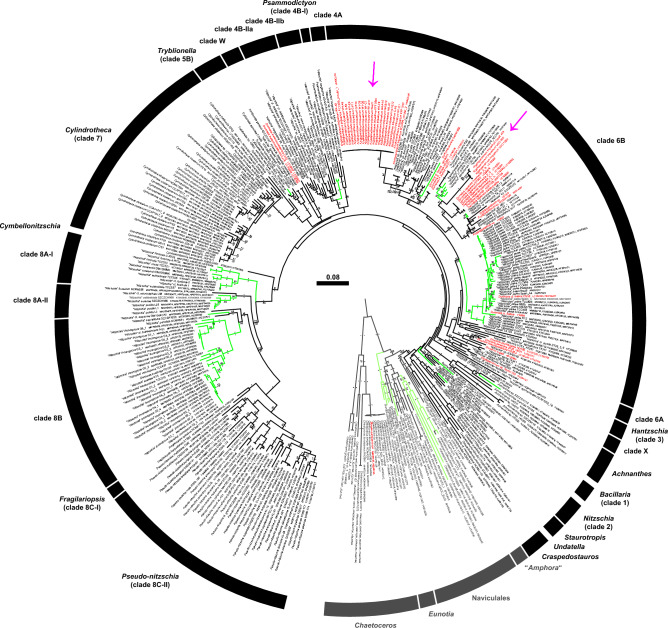
A molecular reference tree recognising major groups of Bacillariaceae (created using Adobe Illustrator^©^ CS6; https://www.adobe.com/de/products/illustrator.html). Maximum Likelihood (ML) tree of 317 bacillariacean sequences (with strain number and GenBank accession number information, outgroup accessions are shaded grey) as inferred from an alignment comprising sequences of the rRNA operon, *psb*A, *rbc*L and *psb*C (3117 parsimony-informative positions). Clade labelling follows previous work^66^. Numbers on branches are ML bootstrap (above) and Bayesian probabilities (below) for the clusters (asterisks indicate maximal support values, values under 50 and .90, respectively, are not shown). Note that endosymbionts of Kryptoperidiniaceae (emphasised by red lettering) are scattered over the tree in a highly polyphyletic pattern, accessions assigned to *Kryptoperidinium* are indicated by pink arrows. Freshwater accessions are highlighted by green branches.

The SSU + ITS + LSU + *psb*A + *rbc*L + *pcb*C alignment of diatoms was 1892 + 1221 + 3356 + 1005 + 1620 + 1377 bp long and was composed of 545 + 797 + 494 + 219 + 603 + 459 parsimony-informative sites (30%, mean of 8.73 per terminal taxon) and 5417 distinct RAxML alignment patterns. Topological inconsistencies between nuclear and plastid loci were rare and—if present—referred to internal branching of, for example, *Chaetoceros*, *Cylindrotheca* and *Pseudo-nitzschia*. Figure 9 (Supplementary Figure 9) shows the best-scoring ML tree (− ln = 140,379.69), with many nodes having high if not maximal statistical support. Although some deeper nodes had only low support, Bacillariaceae (84LBS, 1.00BPP) were monophyletic with respect to the successive close relatives “*Amphora*” (84LBS, 1.00BPP), Naviculales (97LBS, 1.00BPP), *Eunotia* (100LBS, 1.00BPP) and *Chaetoceros* (100LBS, 1.00BPP). Dinophyte endosymbionts did not constitute a monophyletic group, with that of *Blixaea* nesting with *Chaetoceros tenuissimus* (100LBS) and those of *Dinothrix*, *Durinskia* and *Kryptoperidinium* scattered over the tree in a polyphyletic pattern.

Endosymbiont ITS sequences collected in the Baltic Sea were the same as each other but with two exceptions (i.e., W4-A6, W4-F1, from Wismar marina), which differed in a unique 5 bp insertion from the others. In the phylogenetic tree, endosymbionts of *K. triquetrum* were nested in clade 6B (51LBS) with other dinophyte endosymbionts, but comprised two clades, which were only distantly related to each other: Accessions from the Baltic Sea constituted a group (91LBS, 1.00BPP) with free-living species determined as “*Nitzschia*” *lembiformis*, “*Nitzschia*” *pusilla* and “*Nitzschia*” *thermalis*; accessions from the Atlantic Ocean and the Mediterranean Sea comprised a clade (97LBS, 1.00BPP) with predominantly freshwater taxa including “*Nitzschia*” *draveillensis*. The latter clade showed a close relationship (96LBS, 1.00BPP) to sequences retrieved from endosymbionts of *Durinskia capensis* (100LBS, 1.00BPP). Notably, the ITS sequence of strain GeoB 459 was almost identical (> 99% similarity) to an ITS sequence (AY574381) derived from free-living “*Nitzschia*” *pusilla* (89LBS, .91BPP).

## Discussion

### Repeated uptake of endosymbiont partners

The establishment of permanent chloroplast organelles in eukaryotic cells from formerly free-living cooperative partners is a multi-step process of evolution^3,5,6^. In Archaeplastida, the primary endosymbiosis event has resulted in a mutual dependence of the partners, which is absolute^67^—neither the chloroplasts nor the host cells are able to survive without each other under natural conditions^68^. Replication is also synchronised, and there has been an extensive exchange of genetic material between the compartments nucleus and plastid^69^. At the levels of secondary and tertiary endosymbiosis, the amount and maturity of such cooperation are highly diverse: Some species and species groups have already developed a similar dependency as in algae with primary endosymbiosis (e.g., cryptophytes^70^), others are still at the dawn of such a progression^7^.

The present case of *Kryptoperidinium* as integral part of the dinotoms certainly represent an early stage of chloroplast establishment, and some of the multiple steps can be brought into a sequence of evolutionary events: Replication between hosts and diatoms appears already synchronised^53,71,72^, but almost intact cell anatomy of the endosymbionts is retained^23,35,39,57^, and genome reduction is still insignificant^27,73–75^. Nevertheless, it has been suggested that the endosymbionts of Kryptoperidiniaceae are hosted permanently and inherited vertically after a single, ancient engulfment event^65,76,77^. If the chloroplasts are inherited vertically, then endosymbionts would form a monophyletic group in the trees derived from molecular sequence data (like chloroplasts nesting in cyanobacteria^12,13,15^). However, the phylogenetic results clearly reject this hypothesis, and the opposite is the case: The endosymbionts are scattered over the tree, and most of them have closest relatives not among other endosymbionts but among free-living diatoms^7,41^. This conclusion does not only refer to groups of species but even to single species such as *K. triquetrum*, in which there are two distinct and only distantly related groups of endosymbionts in the bacillariacean tree.

The presence of different diatoms in the same host species indicates that tertiary endosymbiosis is not yet a stable system in Kryptoperidiniaceae, and the question arises whether the endosymbiosis is entirely obligate (or some individuals may be able to survive entirely heterotrophically, lacking any endosymbiont). Anyhow, recent work on *Durinskia* shows that endosymbiont establishment even at the species level may reflect different evolutionary stages^42^. One species, namely *Duriskia capensis*, keeps newly phagocytosed diatoms for only two months, whereas other species are able to maintain diatoms for undetermined periods of time. Strains assigned to *Kryptoperidinium* have kept their endosymbiont for more than 30 years in cultivation^78^. Nevertheless, cells of Kryptoperidiniaceae with only one stainable nucleus under light microscopy have been mentioned^36,52,64^, but such reports should be taken with reservation in *Kryptoperidinium* (not least because of the methodological challenges). All strains of *K. triquetrum* studied here are bi-nucleate and for the moment, the presence of the diatom nucleus is therefore considered an invariable trait of the species.

### Distinct ribotypes of endosymbionts in different regions of the world

Plankton communities may actually consist of both wide spread and more restrictedly distributed species and are assembled to a combination of dispersal potential and ecological selection^46,79–81^. In a number of planktonic dinophytes such as *Alexandrium* (Ostreopsidaceae) and *Scrippsiella* (Thoracosphaeraceae), ITS ribotypes show a global distribution^81,82^. Benthic dinophytes do not show a clear signal, with ITS ribotypes of *Coolia* (Ostreopsidaceae) found worldwide^83^, whereas epiphytic *Ostreopsis* (also Ostreopsidaceae) in fact show a correlation between molecular sequence data and distribution, with genetically distinct Atlantic/Mediterranean *versus* Indo-Pacific populations^84^. In the freshwater environment, there may be some morphological differentiation within species, such as in *Peridinium volzii* between specimens from Europe and Eastern Asia^85^. In the present study, *K. triquetrum* does show a spatial distinction based on multiple gatherings, as the Baltic strains have different endosymbionts in this species than strains from other localities. To the best of our knowledge, this is the first report of such a spatial fragmentation in a planktonic species of dinophytes. It is worth noting again here that the endosymbionts of *K. triquetrum* do not constitute a monophyletic group, but have closest relatives among free-living diatoms.

The spatially regular meeting of the prospective partners is one of the prerequisites at the dawn of chloroplast establishment^3–5,7^. Most members of the Bacillariaceae are benthic algae, living on shallow marine sediments (but also as periphyton and epiliton), whereas dinotoms such as *K. triquetrum* are mainly planktonic forms^32,65^. How precisely a planktonic dinophyte would capture a benthic diatom remains a question for future research. It is currently still under debate whether endemism is an important phenomenon in benthic diatoms^86–88^—if restricted distribution patterns do occur, then the presence of different partners in hosts of different geographical origins would explain the present molecular trees of Bacillariaceae with the endosymbionts included.

Kryptoperidiniaceae are an exceptional model for studying the first steps of organelle establishment, as the excessive reduction of the morphological and biochemical components that has occurred in other photosynthetic groups has not yet taken place. However, research only begins to understand the complex interactions and mutual processes that have led to the diversity of photosynthesis in eukaryotes. In the case of the Kryptoperidiniaceae, evolutionary conclusions suffer from weakly supported phylogenies of the endosymbionts, and improved DNA trees of diatoms are needed. Concatenation of sequences^66,89,90^ is still not universally accepted as the method to reach this aim (similar to the situation in dinophytes). The present attempt of this study follows this path (like it is done also in dinophytes^46,91–93^), although the alignment is still very patchy—these gaps need to be filled in future research. To robustly support the results of *Kryptoperidinium* shown here, multiple collections and strains of one species as well as of closely related species and populations are needed regarding both hosts and endosymbionts.

### Divergent thecal interpretations

Based on the observations of multiple strains from various geographic regions the morphology of accessions assigned to *Kryptoperidinium* I is very consistent, and we are confident that the lineage comprises a single species only (with *K. foliaceum* being a later heterotypic synonym of *K. triquetrum*). This conclusion enables a critical assessment of morphological inconsistencies that are found in the literature. With respect to the thecal plate pattern of *Kryptoperidinium* (Table 2), there is general consensus in the number of postcingular (i.e., five) and antapical plates (i.e., two) of the hypotheca (as frequently present in peridiniod dinophytes), but varying numbers of epithecal, cingular and sulcal plates have been encountered. However, the comparison of historical reports is hampered, because phylogenetic analyses indicate the existence of two, only distantly related clades of *Kryptoperidinium*^64^ having similar appearance^43,52–54^. Unfortunately, most previous morphological studies lack corresponding molecular sequence data and hence, it is difficult to distinguish between observational bias and true morphological differences among evolutionarily divergent clades of *Kryptoperidinium*.

**Table 2:** Plate patterns of *Kryptoperidinium* reported in the literature.

Reference	APC	Apical plates	Intercalary plates	Precingular plates	Cingular plates	Sulcal plates	Postcingular plates	Antapical plates	pl. 1′ p	Molecular data
^55^	po	4 (1 r, 2 vap, 1 map)^a^	2 (2 dap)	7 pr^b^	n.a.	n.a.	5	2	‘Sulcal plate’^c^	‒
^94^	po	3^d^	2	7^e^	n.a	n.a.	5	2	Sulcal plate^f^	‒
^95^	po, X	4	2	7	n.a	3	5	2	Sulcal plate^g^	‒
^96^	po	3^h^	2	7	n.a.	n.a.	5	2	n.a.	‒
^97^	n.a	3–4	2	7	n.a.	n.a.	5	2	n.a.	‒
^98^^i^	po, X	4	2	6–7	n.a.	n.a.	5	2	?^j^	‒
^99^	n.a	3	2	7	n.a.	n.a.	5	2	n.a.	‒
^100^	n.a	3–4	2	7	n.a.	n.a.	5	2	n.a.	‒
^101^^k^	po	4	2	7	n.a.	n.a.	5	5	n.a.	‒
^102^^l^	po, X	4	2	7	6	5	5	2	sa	‒
^52^	po, X	4	2	7	4 or 5^m^	5	5	2	sa	Yes^n^
^53^	n.a	3	2	7	4	n.a.	5	2	sa	‒
^43^	po, X	4	2	7	5	> 5	5	2	sa	‒
^54^^o^	po, X	4	2	7	5	5–6^p^	5	2	sa	Yes^q^
This study	po, X	4	2	7	5	7	5	2	1′ p	Yes

^a^Among the 4 apical plates described and drawn, there is a symmetric first apical plate (‘Rautenplatte’ or 1r), which is considered rare. Based on the present observations the existence is doubtful.

^b^In the drawing, the ventral precingular plate refers to our first apical plate leading to six precingular plates. The L-shaped precingular plate 7′′ was probably overlooked.

^c^This plate was not labelled but considered as sulcal plate: “Längsfurche in der Form eines dreieckigen Feldes auf die Epivalva übergreifend” (translated: “sulcus with a triangular-shaped plate extends into the epitheca”).

^d^There are three plates surrounding the APC (i.e., three apical plates), and the large ventral plate (usually considered as plate 1′) is interpreted as precingular plate. The elongated X-plate separating plate 1′ from the pore plate was obviously overlooked.

^e^These seven plates include the ventral plate (1′), that six true precingular plates were observed. However, the L-shaped precingular plate 7′′ might have been overlooked.

^f^“A small triangular plate dividing the ends of the girdle apparently belongs to the ventral area”.

^g^“In der Längsfurche befinden sich drei Platten, … während die dritte obere die nicht geschlossene Äquatorialfurche ergänzt und auch besonders an der linken Seite der Rautenplatte auf die Epivalva übergreift“ (translated: “In the sulcus, there are three plates, … whereas the anterior third plate complete the cingulum and extends on the left side of plate 1′ into the epitheca”).

^h^“Occasionally with four apicals”.

^i^Determined as *Peridinium foliaceum.*

^j^The ventral area between the start and the end of the cingulum is formed by a “fermée par une plaque supplementaire” (translated: “supplementary plate”).

^k^In his doctoral thesis, Takeo Horiguchi presented a plate pattern, for what he determined as “Glenodinium foliaceum” -stage of a *G. foliaceum*‒*Dinothrix paradoxa* complex. These cells, however, differ fundamentally from *K. foliaceum* by a significant cingular displacement, by lack of dorso-ventral compression and by the presence of a symmetrical plate 1′ in mid-ventral position.

^l^Determined as *Peridinium foliaceum.*

^m^Among five strains, they described four strains with four and one strain with five cingular plates.

^n^SSU and ITS data for three of the five studied strains available (i.e., NCMA1326, SC, UTEX1688), which all belong to *Kryptoperidinium* II.

^o^As *Kryptoperidinium* sp.

^p^There is no conclusive information, how this uncertainty is inferred or has to be interpreted. In Fig. 6D, four sulcal plates are labelled (with figure legend), and the presence of two additional sulcal plates is indicated by asterisks.

^q^The two analysed strains cluster with sequences corresponding to type material of *Kryptoperidinium triquetrum.*

The first detailed thecal pattern of *Kryptoperidinium* is based on material collected in the German Baltic Sea^55^, likely representing *K. triquetrum* (as *Kryptoperidinium* II has not been recorded from there so far). Anyhow, one of the schematic drawings of an apical view (later reproduced^97^) differs significantly from all subsequent reports, namely in the symmetric and narrow plate 1′ having a central ventral position. This arrangement was rarely seen^55^ and if so, then this ‘Rautenplatte’ was mostly fused with either plate 2′ (plate 1vap in E. Lindemann’s notation) or with what was considered plate 1′′ (1pr in E. Lindemann’s notation; note that E. Lindemann counted plates clockwise and thus different from the common Kofoidean notation). Such a ‘fusion’ in E. Lindemann’s interpretation then leads to a large, asymmetric and slightly displaced ventral plate corresponding to our plate 1′ a. A symmetric, central, narrow plate 1′ was never observed in the present study and thus, the observation^55^ should be taken with caution. Plates of *Kryptoperidinium* are thin and difficult to study, and it is possible that E. Lindemann erroneously interpreted artificially wrinkled plates dissembling the presence of a central, symmetric ‘Rautenplatte’ as a seeming indication of the close relationship between *Kryptoperidinium* and species of *Peridinium*. In any case, E. Lindemann’s number of epithecal plates is (without a separate, narrow ‘Rautenplatte’) lower by 1 compared to the present (and other) observations, because he probably missed the narrow plate 7′′ (as inferred from his drawings).

One year after E. Lindemann’s survey, three apical and seven precingular plates have been reported^94^. In this case, plate 1′ (in the present interpretation) was considered an element of the precingular plate series, and the correspondingly divergent plate pattern with (four apical and) only six precingular plates may result from neglecting again the narrow plate 7′′. The (mis-)interpretation of an apical as precingular plate has found its way into plate formulas provided in original literature^94^ and also in seminal taxonomic compilations^97,100–103^. They all specify three or four apical plates for *K. triquetrum* and thus create the impression of intraspecific variability regarding the plate numbers of the apical series. Considerable confusion also arose by the report of seven precingular though only three apical plates in cells from the Rio de Vigo estuary^53^. This Baiona strain is unfortunately lost, but another strain (VGO 1124) isolated from the same bloom (Isabel Bravo, *pers. comm.*) as well as strain VGO 556 from the nearby Ulla estuary clearly exhibit the usual plate pattern of *K. triquetrum* with four apical plates (Figs. S15, S16). Thus, the presence of three apical plates^53^ is likely a misinterpretation due to difficulties to observe lateral sutures in this compressed species.

The same difficulty refers to unequivocal detection of lateral sutures of cingular plates and thus likely explains the report of six cingular plates^102^ or of four cingular plates for the Baiona strain^53^. However, five cingular plates are clearly identified in the present material from the type locality as well as in the Spanish strains VGO 556 and VGO 1124, that they appear as correct and invariable number for *K. triquetrum*. This conclusion is also confirmed by other studies^43^, in one case even in combination with molecular data^54^, agreeing with the present sequences gained from the type material. Three strains of *Kryptoperidinium* II may have four cingular plates^52^. It cannot be excluded that the two clades of *Kryptoperidinium* differ by their number of cingular plates, but this needs confirmation by additional analyses of the plate patterns, particularly of strains assigned to *Kryptoperidinium* II.

**Figure 10:**
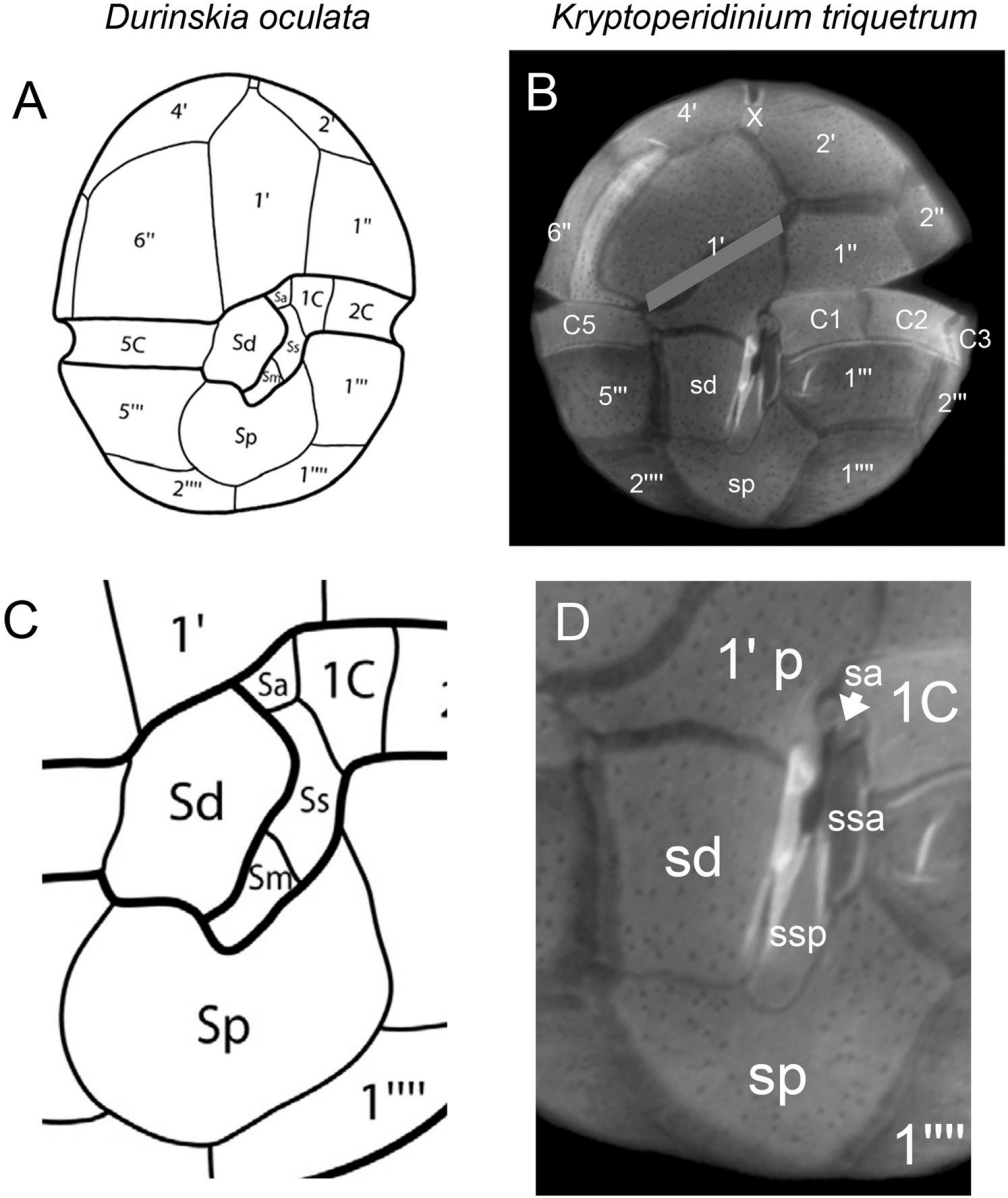
Plate pattern comparison of *Durinskia oculata* (**A,C**) and *Kryptoperidinium triquetrum* (**B,D**) of whole cells in ventral view (**A,B**) and of the sulcal area (**C,D**). (**A,C**) redrawn^32^. In (**B**), a grey bar is hiding the undulating suture indicating that both plates likely belong to a large, symmetric first apical plate. Note that labelling of small sulcal plates in (**C,D**) differ, because *K. triquetrum* has two sulcal plates in median position (Fig. 4I). They are either absent from, or not detected yet (as they might be hidden behind the large Sd plate), for *D. oculata*.

For most species of dinophytes, number and arrangement of plates in the sulcal area are particularly difficult to ascertain. At a first glance, *Kryptoperidinium* appears easy to interpret, having three major sulcal plates forming a vertical row in the central ventral area^95^. The anterior plate is irregularly shaped and partly extends into the epitheca, and the interpretation as a sulcal plate^55^ was followed by all subsequent authors (Table 2). The present detailed analyses of sulcal plates, and the comparison of the ventral plate arrangement with other Kryptoperidiniaceae, allow for an alternative interpretation of this particular thecal element, which is usually labelled as anterior sulcal plate (Fig. 10) (Supplementary Figure 10). Particularly, the ventral view, and the sulcal plate arrangement of *Durinskia oculata*^32^, make an oblique split of an initially symmetric plate 1′ into an anterior (1′ a) and posterior part (1′ p) plausible for *K. triquetrum* (Fig. 10). This interpretation is supported by the unusually undulating course of the suggested split suture. Moreover, a very small and hook-shaped plate in the central sulcal area, adjacent to the area where the flagella emerge, conforms in shape and position with the anterior sulcal plate again of *D. oculata* (Fig. 10). This plate is interpreted here as anterior sulcal plate of *Kryptoperidinium* for the first time and has been already depicted but not labelled or discussed earlier (Figs. 2F ^43^, 6D ^54^). Excluding plate 1′ p from the sulcal series, the present detailed analysis reveals the number of seven sulcal plates but because of the complex three-dimensional structure of the sulcal area with a tubular element in the centre, the small plates smp and sma are hard to detect in LM and are clearly identifiable by SEM only.

## Taxonomic activity

***Kryptoperidinium triquetrum*** **(Ehrenb.) Tillmann, Gottschling, Elbr., Kusber & Hoppenrath.** Phyotaxa 391: 157. 2019, basionym: *Glenodinium triquetrum* Ehrenb., Ber. Bekanntm. Verh. Königl. Preuss. Akad. Wiss. Berlin 1840: 200. 1840. *Heterocapsa triquetra* (Ehrenb.) F. Stein, Der Organismus der Flagellaten nach eigenen Forschungen in systematischer Reihenfolge bearbeitet 3.2: 13. 1883.—Lectotype^61^: [unpubl. illustration] Baltic Sea, off Germany, Mecklenburg-Vorpommern, Wismar, 5 Sep 1840 [non-fossil]: Ch.G. Ehrenberg s.n., the lower of the two cells showing a flagellum present on drawing No. 674 (BHUPM!).—**Epitype, designated here**: [illustration: Fig. 2A‒D] Baltic Sea, off Germany, Mecklenburg-Vorpommern, Wismar (53° 54.57′ N, 11° 26.09′ E), 18 Sep 2019 [non-fossil]: U. Tillmann, M. Gottschling & A. Kremp [U. Tillmann] W4-A6.

Other original elements: a dried mounted specimen comprising several non-fossil individuals from Baltic Sea, off Germany, Mecklenburg-Vorpommern, Wismar, without date [non-fossil]: Ch.G. Ehrenberg s.n. (BHUPM Infusionsthierchen XCIX: 540099-6!^104^; indexed as “Glenodinium triquetrum, Wismar, Hafen”).

** = *Glenodinium foliaceum*** **F.Stein**, Der Organismus der Flagellaten nach eigenen Forschungen in systematischer Reihenfolge bearbeitet 3.2: pl. III 22‒26. 1883. *Heterocapsa foliacea* (F.Stein) Daday, *nom. corr.* (ICN Art. 23.5), Természetrajzi Füzetek 11: [76, ]99. 1888. *Kryptoperidinium foliaceum* (F.Stein) Er.Lindem., Botani-sches Archiv. Zeitschrift für die gesamte Botanik 5: 116‒117, Figs. 12‒20. 1924. *Peridinium foliaceum* (F.Stein) Biecheler, Bull. Biol. France Belgique/Supplément 36: 77[‒81], Figs. 46‒49. 1952.—Lectotype^63^: [illustration] Baltic Sea, off Germany. Mecklenburg-Vorpommern, Wismar, probably late summer 1879^105^ [non-fossil]: F. von Stein, Der Organismus der Flagellaten nach eigenen Forschungen in systematischer Reihenfolge bearbeitet 3.2: pl. III 24!—**Epitype, designated here**: [illustration: Fig. S4K‒L] Baltic Sea, off Germany, Mecklenburg-Vorpommern, Wismar (53° 54.81′ N, 11° 26.07′ E), 18 Sep 2019 [non-fossil]: U. Tillmann, M. Gottschling & A. Kremp [U. Tillmann] W1-E4.

We tried several techniques to prepare physical epitypes (e.g., permanent slides for light microscopy and SEM-stubs—all deposited at B, M and CEDiT under the accession codes B 40 0045589 through B 40 0045562, M-0328661 through M-0328671, CEDiT2022RM152 through CEDiT2022RM158 and CEDiT2023RM161 through CEDiT2023RM164), but none of them were successful to show the characteristic plate pattern. Exceptionally and differently from our previous approaches, we therefore decided to use illustrations here for the designation of epitypes (ICN Art. 40.5). Pictures were taken from cells or their remnants, which were cultivated as strains established from a single cell. Thus, the epitypes do not exhibit DNA intrinsically, but are linked to material with corresponding genetic information. The nomenclatural acts have been registered in PhycoBank under http://phycobank.org/103280 and http://phycobank.org/103281, respectively.

## Methods

### Strains, cell isolation, cultivation

A total of 25 strains of *Kryptoperidinium* were inspected in the course of this study (Table 1, Supplementary Table S1). Of these, 4 strains were provided by the FINMARI culture collection/SYKE Marine Research Centre and Tvärminne Zoological station or from the VGOHAB culture collection of Vigo (Spain), and one strain (GeoB 459) was isolated in 2010 from the Aegean Sea as part of the Mediterranean Sea. Eighteen strains were newly isolated in 2019 from samples collected in the German Baltic Sea off Greifswald (54° 06.01′ N, 13° 23.66′ E; salinity 7.7, water temperature 14.9 °C) and Wismar. In Wismar, two different localities were sampled, one at the Wendorf pier (53° 54.81′ N, 11° 26.07′ E; salinity 12.2, water temperature 14.1 °C) and the other at a small marina (53° 54.57′ N, 11° 26.09′ E; salinity 12.2, water temperature 14.5 °C). Two additional strains were isolated at Wismar marina also in 2020.

At all Baltic localities, both a surface water sample and a phytoplankton net tow sample (20 µm mesh size) were taken, and single cells were isolated by micro-capillary into 96-well plates filled with 0.2 mL filtered water from the sample site. Plates were incubated at 15 °C under a photon flux density of 80 µmol m^−2^ s^−1^ on a 16:8 h light:dark photocycle in a controlled environment growth chamber (Sanyo Biomedica MIR 252; Wood Dale, USA‒IL). Established strains of *Kryptoperidinium* were subsequently grown at the culture conditions described above in a natural seawater medium consisting of sterile filtered (0.2 µm VacuCap filters; Pall Life Sciences; Dreieich, Germany) and diluted North Sea water with a salinity of about 15. Nutrients were added corresponding to 50% of K-medium^106^, slightly modified by replacing the organic phosphorous source with 3.62 µM Na_2_HPO_4_.

For DNA harvest, cells were collected by centrifugation (Eppendorf 5810R; Hamburg, Germany) in 50 mL centrifugation tubes at 3220×*g* for 10 min. Cell pellets were transferred with 0.5 mL lysis buffer (SL1, provided by the NucleoSpin Soil DNA extraction Kit; Macherey–Nagel; Düren, Germany) to 1 mL microtubes and stored frozen (− 20 °C) for subsequent DNA extraction.

### Microscopy

Observation of living or fixed cells (formaldehyde: 1% final concentration, or neutral Lugol-fixed: 1% final concentration) was carried out using an inverted microscope (Axiovert 200 M; Zeiss; Munich, Germany) and a compound microscope (Axiovert 2; Zeiss), both equipped with epifluorescence and differential interference contrast optics. Living cells were recorded using a digital video camera (Gryphax, Jenoptik; Jena, Germany) at full-HD resolution. Single frame micrographs were extracted using Corel Video Studio software (Version X8 pro; Corel; Ottawa, Canada). Images of fixed cells were taken with a digital camera (Axiocam MRc5; Zeiss).

Light microscopic (LM) examination of thecal plates was performed on fixed cells (neutral Lugol) stained with Solophenyl Flavine (Carbosynth, Compton, UK), a fluorescent dye specific to cellulose^107^. Epifluorescence microscopy was used to observe chloroplasts (filter set 09; Zeiss) and to determine the shape and location of the nucleus (UV excitation, filter set 01; Zeiss) after staining of formalin-fixed cells with 4′,6-diamidino-2-phenylindole (DAPI, 0.1 μg mL^−1^ final concentration) for 10 min. Cell length and width were measured at × 1000 microscopic magnification using freshly fixed cells (formaldehyde, 1% final concentration) from dense but healthy and growing strains (based on stereomicroscopic inspection of the living material) at late exponential phase and the Axiovision software (Zeiss).

For scanning electron microscope (SEM), Lugol-fixed cells were collected by gentle filtration on 3 µm pore-size polycarbonate filters and were subsequently processed for SEM (FEI Quanta FEG 200; Eindhoven, the Netherlands) as described previously^108^.

### Molecular phylogenetics

Genomic DNA was extracted following the manufacturers’ instructions of the NucleoSpin Soil DNA extraction Kit (Macherey–Nagel, Düren, Germany) with an additional cell disruption step within the beat tubes; the samples were shaken in a FastPrep FP120 cell disrupter (Qbiogene, Carlsbad, USA‒CA) for 45 s and another 30 s at a speed of 4.0 m s^−1^. For the elution step, 50 μL of the provided elution buffer were spinned through the column, and elution was subsequently repeated with another 50 μL to increase the DNA yield. For the *Kryptoperidinium* host and for the endosymbiont, various regions of the ribosomal RNA (rRNA) were amplified using several primer sets (specific to dinophytes and their endosymbionts, respectively: Table S2) and temperature conditions (Table S3). Each reaction contained 16.3 μL of ultra-pure H_2_O, 2.0 μL of HotMaster Taq buffer (5Prime; Hamburg, Germany), 0.2 μL of each primer (10 μM), 0.2 μL of dNTPs (10 μM), 0.1 μL of Taq Polymerase (Quantabio; Beverly, USA‒MA) and 1.0 μL of extracted DNA template (10 ng μL^−1^) to a final reaction volume of 20 μL. Afterwards, PCRs were conducted in a Nexus Gradient Mastercycler (Eppendorf), and PCR amplicons were inspected on a 1% agarose gel (in TE buffer, 70 mV, 30 min) to verify the expected length. If needed, nested PCR was performed with primer pairs indicated in Table S2. Chloroplast loci were amplified and sequences as described earlier^41^.

Amplicon purification followed the instructions of the NucleoSpin Gel and PCR clean-up kit (Macherey–Nagel), and PCR products were sequenced directly in both directions on an ABI PRISM 3730XL (Applied Biosystems; Waltham, USA‒MA) using the ABI Big-Dye dye-terminator technique (Applied Biosystems) accordingly to the manufacturer’s recommendations. Raw sequence data were processed using the CLC Genomics Workbench 12 (Qiagen; Hilden, Germany). Sequences were edited and assembled using Sequencher™v5.1 (Gene Codes; Ann Arbor, USA‒MI). For visual comparison of the edited sequences, the alignment editor ′Se-Al′ (http://tree.bio.ed.ac.uk/software/seal/) was used.

To compute a dinophyte reference tree inferred from a concatenated rRNA alignment^46,49^, we compiled a systematically representative set comprising 101 peridinialean dinophytes including 56 Kryptoperidiniaceae (Table S1). To compute a reference tree of Bacillariaceae inferred from a concatenated alignment comprising sequences of the rRNA operon, *psb*A, *rbc*L and *psb*C we used a previous alignment^41^ and enriched the matrix with other relevant sequences^66^, also identified based on Blast searches^109^ of the newly gained sequences from the endosymbionts. To build the alignment, separate matrices of the rRNA operon and the genes were constructed, aligned using ‘MAFFT’ v6.502a^110^, and the ‒qinsi option to take into account the secondary structure of rRNA, and concatenated afterwards. The aligned matrices are available in the Supplementary Information.

Phylogenetic analyses were carried out using Maximum Likelihood (ML) and Bayesian approaches, as described previously^91^, using the resources available from the CIPRES Science Gateway^111^. Briefly, the Bayesian analysis was performed using ‘MrBayes’ v3.2.7a^112^ (freely available at http://mrbayes.sourceforge.net/download.php) under the GTR + Γ substitution model and the random-addition-sequence method with 10 replicates. We ran two independent analyses of four chains (one cold and three heated) with 20,000,000 generations, sampled every 1000th cycle, with an appropriate burn-in (10%) inferred from evaluation of the trace files using Tracer v1.7.1^113^. For the ML calculations, the MPI version of ‘RAxML’ v8.2.4^114^ (freely available at http://www.exelixis-lab.org/) was applied using the GTR + Γ substitution model under the CAT approximation. We determined the best-scoring ML tree and performed 1000 non-parametric bootstrap replicates (rapid analysis) in a single step. The phylogenetic inferences were run in partitions under GTR (MrBayes) or in one block (RAxML, as it does not allow for empty sequences within partitions). Statistical support values (LBS: ML bootstrap support; BPP: Bayesian posterior probabilities) were drawn on the resulting, best-scoring tree.

## Supplementary Information

Supplementary Video 1.

Supplementary Information 1.

Supplementary Information 2.

Supplementary Information 3.

Supplementary Figure 1.

Supplementary Figure 2.

Supplementary Figure 3.

Supplementary Figure 4.

Supplementary Figure 5.

Supplementary Figure 6.

Supplementary Figure 7.

Supplementary Figure 8.

Supplementary Figure 9.

Supplementary Figure 10.

## Data Availability

The sequence data generated during the current study are available in the GenBank repository (https://www.ncbi.nlm.nih.gov/nuccore). For corresponding accessions numbers, one may refer to the extensive voucher list (Table S1) in the Supplementary Information.
