# Supplementary material for: Spatial fragmentation in the distribution of diatom endosymbionts from the taxonomically clarified dinophyte Kryptoperidinium triquetrum (= Kryptoperidinium foliaceum, Peridiniales)
Source: Sci Rep. 2023 May 26;13:8593. doi: 10.1038/s41598-023-32949-y (PMC10219988; doi:10.1038/s41598-023-32949-y)
Supplement: Supplementary file 4 — Supplementary Information 3. [file 41598_2023_32949_MOESM4_ESM.pdf]

Spatial fragmentation in the distribution of diatom endosymbionts

from the taxonomically clarified dinophyte

Kryptoperidinium triquetrum

(= *Kryptoperidinium foliaceum*, Peridiniales)

Urban Tillmann, Stephan Wietkamp, Juliane Kretschmann, Juliana Chacón, Marc Gottschling

Supplementary information

Tables

Table S1: Voucher list. All names are given under the rules of the ICN, with the author standard forms[1]. Abbreviation: n.inf., no information. If 'holotype' or 'epitype' is noted for a species name, then it refers to material, from which the type was prepared.

Species name with author	Strain No.	Locality	Date	Collector(s) [isolator]	GenBankNo(s)	Reference
Aduncodinium glandulum (Herdman) N.S.Kang, H.J.Jeong & Moestrup	n.inf.	western North Pacific, East China Sea, off South Korea: Gyeongsangnam-do, Masan Bay (35°11'N, 128°35'E)	2013	N.S. Kang s.n.	LK934662 (SSU+ITS+LSU)	2
Amphidiniopsis uroensis Toriumi, Yoshimatsu & J.D.Dodge	uro8	western North Pacific, off Japan: Kōchi (33°19'N, 134°10'E)	Apr 18, 2014	A. Yamaguchi s.n.	LC191235 (SSU), LC191250 (LSU)	3
Apocalathium malmogiense (G.Sjöstedt) Craveiro, Daugbjerg, Moestrup & Calado (reference material)	SHTV1	Baltic Sea, off Finland: Uusimaa, Raseborg, Tvärminne (59°50'N, 23°15'E)	2002	A. Kremp s.n.	KF751923 (SSU+ITS+LSU)	4–8
Archaeperidinium minutum (Kof.) Jørg.	n.inf.	eastern North Pacific, off Canada: British Columbia, Victoria	Mar 21, 2006	n.inf.	AB564309 (SSU), AB564310 (LSU)	9
Blastodinium contortum Chatton [isolated from *Paracalanus* cf. *parvus* (Claus, 1863)]	n.inf.	eastern North Pacific, off USA–CA: Gulf of California, station 3 (24°14'N, 110°20'W)	Jun 11, 2008	n.inf.	FJ228701 (SSU+ITS+LSU)	10
Blastodinium crassum Chatton [isolated from *Paracalanus* cf. *parvus* (Claus, 1863)]	n.inf.	eastern North Pacific, off USA–CA: Gulf of California, station 2 (24°13'N, 110°20'W)	Jun 10, 2008	n.inf.	FJ228702 (SSU+ITS+LSU)	10
Blixaea quinquecornis (T.H.Abé) Gottschling	n.inf.	western North Pacific, Sea of Japan, off Japan: Hokkaidō, Shiribeshi, Otaru (43°10'N, 141°01'E)	Sep 9, 2005	n.inf.	AB246744 (SSU), AB246746 (esSSU), AB246745 (rbcL)	11

*Caladoa arcachonensis Z.*Luo, K.N.Mert. & H.Gu (holotype)	TIO278	eastern North Atlantic, Bay of Biscay, off France, Arcachon Bay (44°38'N, 1°04'W)	Apr, 2016	n.inf.	MK012071 (SSU), MK012081 (ITS), MK012076 (LSU)	12
†*Calciodinellum operosum* Deflandre (reference material)	SZN74	Mediterranean Sea, Tyrrhenian Sea, off Italy: Campania, Naples (40°43'N, 14°10'W)	n.inf.	M. Montresor	KF751922 (SSU+ITS+LSU)	5, 8, 13–16
Dinothrix paradoxa Pascher	2020-HS02	western North Pacific, off Japan: Kanagawa, Miura (35°12'N, 139°36'E)	n.inf.	H. Sakai	LC583318 (SSU)	17
Dinothrix phymatodea Norico Yamada & T.Horig. (holotype)	HG180	western North Pacific, East China Sea, off Japan: Okinawa, Shimajiri, Hanashiro (26°07'N, 127°45'E)	n.inf.	n.inf.	LC054946 (SSU), LC192339 (esSSU), LC192328 (rbcL)	18–19
Dinothrix pseudoparadoxa Norico Yamada & T.Horig. (holotype)	HG204	western Indian Ocean, off South Africa: KwaZulu-Natal, Marina Beach (30°57'S, 30°18'E)	n.inf.	n.inf.	LC054947 (SSU), LC192340 (esSSU), LC192329 (rbcL)	18–19
Dinothrix quadrilobata (T.Horig. & Pienaar) R.Onuma & T.Horig.	2020-RO01	western North Pacific, East China Sea, off Japan: Okinawa, Ishigaki (30°45'N, 131°00'E)		R. Onuma	LC583319 (SSU)	17
Dinothrix rugata (Tam. & T.Horig.) Norico Yamada & T.Horig.	n.inf.	western North Pacific, Mirconesia, off Palau: Mecherchar Island	May 22, 2002	n.inf.	AB195668 (SSU), AB195669 (rbcL)	20
Duboscquodinium collinii Grassé [isolated from *Eutintinnus fraknoii* (Daday, 1887)]	VSM11	western Mediterranean Sea, off France: Alpes-Maritimes, Nice, Villefranche-sur-Mer (43°41'N, 7°19'E)	Sep 10, 2009	n.inf.	HM483399 (SSU+ITS+LSU)	21
Durinskia agilis (Kof. & Swezy) Saburova, Chomérat & Hoppenrath	IFR10-452	Persian Gulf, off Kuwait: Hawalli, Ras Salmiya	Nov 28, 2010	n.inf.	JF514515 (SSU)	22
Durinskia cf. *baltica* (Levander) Carty & El.R.Cox	CS38 (≡ UTEX1563)	USA–CA: Salton Sea	Oct, 1965	[A.R. Loeblich III.] s.n.	AF231803 (SSU), LC192343 (esSSU), NC_014287 (atpB, psaA, psaB, psbA, psbC, rbcL)	19, 23–24

Durinskia cf. *baltica* (Levander) Carty & El.R.Cox	DbDH2	western North Pacific, Yellow Sea, off South Korea: Seoul, Dongho	Mar 11, 2013	n.inf.	KT371442 (LSU)	25
Durinskia cf. *baltica* (Levander) Carty & El.R.Cox	DbDH3	western North Pacific, Yellow Sea, off South Korea: Seoul, Dongho	Jun 13, 2013	n.inf.	KT371448 (LSU)	25
Durinskia cf. *baltica* (Levander) Carty & El.R.Cox	HG171	western North Pacific, East China Sea, off Japan: Okinawa, Tokashiki	n.inf.	n.inf.	LC054925 (SSU), LC192341 (esSSU), LC192335 (rbcL)	18–19
Durinskia cf. *baltica* (Levander) Carty & El.R.Cox	HG265	western North Pacific, East China Sea, off Japan: Okinawa, Odo	n.inf.	n.inf.	LC054926 (SSU), LC192336 (rbcL)	18–19
Durinskia cf. *baltica* (Levander) Carty & El.R.Cox	n.inf.	Japan, Kyūshū, Kagoshima, Amami-Ōshima	Jul 12, 1998	n.inf.	AB195670 (rbcL)	20
Durinskia cf. *baltica* (Levander) Carty & El.R.Cox	n.inf.	n.inf.	n.inf.	n.inf.	Y10566 (esSSU)	26
Durinskia capensis Pienaar, Sakai & T.Horig.	Kommetjie 2-A	eastern South Atlantic, off South Africa: Western Cape, Kommetjie	n.inf.	N. Yamada s.n.	LC192320 (SSU)	19
Durinskia capensis Pienaar, Sakai & T.Horig.	Kommetjie 2-B	eastern South Atlantic, off South Africa: Western Cape, Kommetjie	n.inf.	N. Yamada s.n.	LC192322 (SSU)	19
Durinskia capensis Pienaar, Sakai & T.Horig.	Kommetjie 6-A	eastern South Atlantic, off South Africa: Western Cape, Kommetjie	n.inf.	N. Yamada s.n.	LC192324 (SSU)	19
Durinskia capensis Pienaar, Sakai & T.Horig.	Kommetjie 6-B	eastern South Atlantic, off South Africa: Western Cape, Kommetjie	n.inf.	N. Yamada s.n.	LC192323 (SSU)	19
Durinskia capensis Pienaar, Sakai & T.Horig.	NY066	eastern South Atlantic, off South Africa: Western Cape, Kommetjie	n.inf.	R.J. Anderson s.n.	LC385879 (SSU), LC385878 (rbcL)	27
Durinskia capensis Pienaar, Sakai & T.Horig.	Saldanha Bay	eastern South Atlantic, off South Africa: Western Cape, Kommetjie	n.inf.	T. Horiguchi s.n.	LC192321 (SSU)	19

Durinskia kwazulunatalensis Norico Yamada, Sym & T.Horig.	Cx18	western Indian Ocean, off South Africa: KwaZulu-Natal, Marina Beach	n.inf.	n.inf.	LC054928 (SSU), LC192338 (esSSU), LC192325 (rbcL)	18–19
Durinskia kwazulunatalensis Norico Yamada, Sym & T.Horig. (holotype)	Cx22	western Indian Ocean, off South Africa: KwaZulu-Natal, Marina Beach	n.inf.	n.inf.	LC054929 (SSU), LC192337 (esSSU), LC192327 (rbcL)	18–19
Durinskia oculata (F.Stein) Gert Hansen & Flaim (epitype)	GeoM*662 (≡ CCAC6039B, CCCM6005)	Czech Republic: Prague, Hlavní město Praha, Vltava (50°08'N, 14°23'E)	Sep, 2015	J. Kretschmann & M. Gottschling [J. Kretschmann] D043	KY693722 (SSU+ITS+LSU), KY693725 (LSUd8d10), KY693716 (esSSU), KY693717 (esITS), KY693719 (rbcL)	28–29
Durinskia oculata (F.Stein) Gert Hansen & Flaim	I2804*	China: Shandong, Qingdao (36°21'N, 120°10'E)	Oct, 2010	n.inf.	HQ588943 (ITS), HQ588944 (LSU)	30
Durinskia oculata (F.Stein) Gert Hansen & Flaim	n.inf.	China: Xiantao (30°21'N, 113°32'E)	Nov, 2009	n.inf.	GU999528 (SSU), GU999529 (LSU)	30
Ensiculifera tyrrhenica (Balech) Zhun Li, K.N.Mert., Gottschling, H.Gu & H.H.Shin	GeoB*230	Mediterranean Sea, Ionian Sea, off Italy: Gulf of Taranto (40°07'N, 17°19'E)	Oct 26, 2002	D. Saracino [M. Kirsch] s.n.	HQ845329 (SSU+ITS+LSU)	4, 31–32
Gloeodinium montanum Klebs	CCAC0066	Germany: Hessen, Marburg, Nordeck	n.inf.	n.inf.	EF058238 (SSU), EF058258 (LSU)	6, 33
Herdmania litoralis J.D.Dodge	n.inf.	eastern North Pacific, off Canada, Boundary Bay: British Columbia, Centennial Beach	Mar 19, 2009	n.inf.	AB564300 (SSU), AB564306 (LSU)	9
Heterocapsa arctica T.Horig. (holotype)	NCMA445 (≡ NCMA35)	North Atlantic, Baffin Bay (76°15'N, 82°33'W)	Jun 3, 1986 [Jul 28, 1989]	R. Selvin s.n.	KF925338 (SSU), JQ972677 (ITS), AY571372 (LSU)	34–37, Preston & Gilg (unpubl. 2014)
Heterocapsa pseudotriquetra Iwataki, Gert Hansen & Fukuyo	GeoB 222	eastern North Atlantic, off Canary Islands (24°25'N, 17°11'W)	2003	[Meteor 58] [M. Kirsch]	AY499509 (ITS), MF423367 (LSU), MF423369 (LSUd8d10)	4, 38
Heterocapsa steinii Tillmann, Gottschling, Hoppenrath, Kusber & Elbr. (epitype)	UTKG7 (ITS clones 7, 9→14, LSU clones 14→15)	Baltic Sea, off Germany: Schleswig-Holstein, Kiel (54°19'N, 10°09'E)	Aug 7, 2013	A. Tillmann [U. Tillmann] s.n.	MF423350 (SSU), MF423353 (ITS), MF423362 (LSU)	38
Islandinium minutum (Harland & P.C.Reid) Head	IMINCS2	Arctic Ocean (75°22'N, 176°19'E)	Aug 31, 2015	n.inf.	KY129807 (rRNA)	39

Johsia chumphonensis Z.Luo, Na Wang, K.N.Mert. & H.Gu	IshiMM4	western North Pacific, East China Sea, off Japan: Okinawa, Ishigaki, Nosoko (24°29'N, 124°14'E)	Jul 26, 2010	n.inf.	AB999980 (SSU), AB999984 (ITS), AB999988 (LSU)	Prabowo et al. (2017 unpubl.)
Kryptoperidinium sp.	NCMA1326	eastern North Pacific, off USA–CA: San Diego, La Jolla	1984	n.inf.	EF492508 (SSU), FJ823570 (ITS), NC_014267 (atpB, psaA, psaB, psbA, psbC, rbcL)	23, 36, 40–44
Kryptoperidinium sp.	SC (ribotype B)	western North Atlantic, off USA–SC	n.inf.	n.inf.	EF492508 (SSU), FJ823570 (ITS)	40
Kryptoperidinium sp.	UTEX1688 (≡ CCCM974, CS37)	western North Atlantic, Caribbean Sea, off USA–PR: Lajas, La Parguera (18°03'N, 67°04'W)	1965	[P.R. Burkholder] s.n.	EF492508 (SSU), FJ823570 (ITS), Y10567 (esSSU), U31876 (rbcL)	23, 26, 36, 40, 44–46, Yu et al. (2006 unpubl.)
Kryptoperidinium sp.	Xmm11S5	western North Pacific, East China Sea, South China Sea, off China: Guangdong, Xuwen	n.inf.	n.inf.	KU561157 (esSSU)	47
Kryptoperidinium triquetrum (Ehrenb.) Tillmann, Gottschling, Elbr., Kusber & Hoppenrath	A6	Caspian Sea	n.inf.	n.inf.	KY921623 (LSU)	48
Kryptoperidinium triquetrum (Ehrenb.) Tillmann, Gottschling, Elbr., Kusber & Hoppenrath	CCAP1116/3	western North Atlantic, off USA–VA: York River	n.inf.	Ott s.n.	MMETSP0118 (SSU)	37
Kryptoperidinium triquetrum (Ehrenb.) Tillmann, Gottschling, Elbr., Kusber & Hoppenrath	CSST1001	Caspian Sea	n.inf.	n.inf.	KY921622 (LSU)	48
Kryptoperidinium triquetrum (Ehrenb.) Tillmann, Gottschling, Elbr., Kusber & Hoppenrath	GE08	Baltic Sea, off Germany: Mecklenburg-Vorpommern, Greifswald (54°06'N, 13°23'E)	Sep 18, 2019	U. Tillmann, M. Gottschling & A. Kremp [U. Tillmann] s.n.	OP256594 (SSU+ITS+LSU), OP256609 (esSSU+esITS+esLSU), OP264876 (psbA), OP264877 (rbcL)	this study
Kryptoperidinium triquetrum (Ehrenb.) Tillmann, Gottschling, Elbr., Kusber & Hoppenrath	GE10	Baltic Sea, off Germany: Mecklenburg-Vorpommern, Greifswald (54°06'N, 13°23'E)	Sep 18, 2019	U. Tillmann, M. Gottschling & A. Kremp [U. Tillmann] s.n.	OP256617 (esSSU+esITS+esLSU)	this study

Kryptoperidinium triquetrum (Ehrenb.) Tillmann, Gottschling, Elbr., Kusber & Hoppenrath	GeoB 459 (≡ CCAC4765B, CCCM327)	Mediterranean Sea, Aegean Sea, off Greece: Peloponnese, Argolis, Nafplio, Nea Kios (37°35'N, 22°45'E)	Mar, 2010	C. Zinßmeister & S. Söhner [M. Kirsch] GRI00027	KY693721 (SSU+ITS+LSU), KY693724 (LSUd8d10), MF440333 (esSSU), KY693718 (esITS), KY693720 (rbcL)	28–29
Kryptoperidinium triquetrum (Ehrenb.) Tillmann, Gottschling, Elbr., Kusber & Hoppenrath	GF09	Baltic Sea, off Germany: Mecklenburg-Vorpommern, Greifswald (54°06'N, 13°23'E)	Sep 18, 2019	U. Tillmann, M. Gottschling & A. Kremp [U. Tillmann] s.n.	OP256595 (ITS), OP256618 (esSSU+esITS+esLSU)	this study
Kryptoperidinium triquetrum (Ehrenb.) Tillmann, Gottschling, Elbr., Kusber & Hoppenrath	GF11	Baltic Sea, off Germany: Mecklenburg-Vorpommern, Greifswald (54°06'N, 13°23'E)	Sep 18, 2019	U. Tillmann, M. Gottschling & A. Kremp [U. Tillmann] s.n.	OP256616 (esITS+esLSU)	this study
Kryptoperidinium triquetrum (Ehrenb.) Tillmann, Gottschling, Elbr., Kusber & Hoppenrath	KFF0901	Baltic Sea, off Finland: Föglö, Aland	2009	[A. Kremp] s.n.	OP256597 (ITS), OP256607 (esSSU+esITS+esLSU), OP264874 (psbA), OP264875 (rbcL)	this study
Kryptoperidinium triquetrum (Ehrenb.) Tillmann, Gottschling, Elbr., Kusber & Hoppenrath	KFF1001	Baltic Sea, off Finland	2010	[P. Hakanen] s.n.	LT906378 (SSU), OP256596 (ITS), OP256608 (esSSU+esITS+esLSU)	Mikkonen (unpubl. 2017), this study
Kryptoperidinium triquetrum (Ehrenb.) Tillmann, Gottschling, Elbr., Kusber & Hoppenrath	KryCA_Uniss	Italy: Sardinia, Sassari, Alghero, Calich	Apr, 2014	C.T. Satta	MN963958 (SSU), MN963988 (ITS), MN963959 (LSU)	49
Kryptoperidinium triquetrum (Ehrenb.) Tillmann, Gottschling, Elbr., Kusber & Hoppenrath	KrySG_Uniss	Italy: Sardinia, Oristano, Santa Giusta	Aug, 2015	C.T. Satta	MN963957 (SSU), MN963989 (ITS), MN963960 (LSU)	49
Kryptoperidinium triquetrum (Ehrenb.) Tillmann, Gottschling, Elbr., Kusber & Hoppenrath	PCC499	n.inf.	n.inf.	n.inf.	KM062179 (psbA)	50
Kryptoperidinium triquetrum (Ehrenb.) Tillmann, Gottschling, Elbr., Kusber & Hoppenrath	PCC499	n.inf.	n.inf.	n.inf.	KM062180 (psbA)	50
Kryptoperidinium triquetrum (Ehrenb.) Tillmann, Gottschling, Elbr., Kusber & Hoppenrath	SAG38.80	western North Atlantic, off USA–MD: Assateague Island Wildlife Refuge	n.inf.	n.inf.	EF058256 (LSU)	51

Kryptoperidinium triquetrum (Ehrenb.) Tillmann, Gottschling, Elbr., Kusber & Hoppenrath	SCCAP K-0638	western Mediterranean Sea, off Spain	n.inf.	Y. Pazos s.n.	EF052684 (LSU)	52
Kryptoperidinium triquetrum (Ehrenb.) Tillmann, Gottschling, Elbr., Kusber & Hoppenrath	VGO556	Spain: Galicia, Pontevedra, Ulla estuary, Ría de Arousa	Jun 1, 2002	n.inf.	OP256598 (ITS+LSU), OP256710 (esSSU), OP264866 (psbA), OP264867 (rbcL)	this study
Kryptoperidinium triquetrum (Ehrenb.) Tillmann, Gottschling, Elbr., Kusber & Hoppenrath	VGO1124	Spain: Galicia, Pontevedra, Baiona	n.inf.	n.inf.	OP256599 (ITS+LSU), OP256711 (esSSU), OP264868 (psbA), OP264869 (rbcL)	this study
Kryptoperidinium triquetrum (Ehrenb.) Tillmann, Gottschling, Elbr., Kusber & Hoppenrath	W1C06	Baltic Sea, off Germany: Mecklenburg-Vorpommern, Wismar (53°55'N, 11°26'E)	Sep 18, 2019	U. Tillmann, M. Gottschling & A. Kremp [U. Tillmann] s.n.	OP256586 (ITS), OP256615 (esSSU+esITS+esLSU)	this study
Kryptoperidinium triquetrum (Ehrenb.) Tillmann, Gottschling, Elbr., Kusber & Hoppenrath	W1C07	Baltic Sea, off Germany: Mecklenburg-Vorpommern, Wismar (53°55'N, 11°26'E)	Sep 18, 2019	U. Tillmann, M. Gottschling & A. Kremp [U. Tillmann] s.n.	OP256592 (ITS), OP256614 (esSSU+esITS+esLSU)	this study
Kryptoperidinium triquetrum (Ehrenb.) Tillmann, Gottschling, Elbr., Kusber & Hoppenrath	W1D01	Baltic Sea, off Germany: Mecklenburg-Vorpommern, Wismar (53°55'N, 11°26'E)	Sep 18, 2019	U. Tillmann, M. Gottschling & A. Kremp [U. Tillmann] s.n.	OP256585 (ITS), OP256606 (esSSU+esITS+esLSU)	this study
Kryptoperidinium triquetrum (Ehrenb.) Tillmann, Gottschling, Elbr., Kusber & Hoppenrath	W1D06	Baltic Sea, off Germany: Mecklenburg-Vorpommern, Wismar (53°55'N, 11°26'E)	Sep 18, 2019	U. Tillmann, M. Gottschling & A. Kremp [U. Tillmann] s.n.	OP256591 (ITS), OP256613 (esSSU+esITS+esLSU)	this study
Kryptoperidinium triquetrum (Ehrenb.) Tillmann, Gottschling, Elbr., Kusber & Hoppenrath	W1D11	Baltic Sea, off Germany: Mecklenburg-Vorpommern, Wismar (53°55'N, 11°26'E)	Sep 18, 2019	U. Tillmann, M. Gottschling & A. Kremp [U. Tillmann] s.n.	OP256578 (SSU+ITS+LSU), OP256709 (esSSU)	this study
Kryptoperidinium triquetrum (Ehrenb.) Tillmann, Gottschling, Elbr., Kusber & Hoppenrath (epitype of *Glenodinium foliaceum* F.Stein)	W1E04 (≡ CCAC9297B)	Baltic Sea, off Germany: Mecklenburg-Vorpommern, Wismar (53°55'N, 11°26'E)	Sep 18, 2019	U. Tillmann, M. Gottschling & A. Kremp [U. Tillmann] s.n.	OP256580 (SSU+ITS+LSU), OP256601 (esSSU+esITS), OP264872 (psbA), OP264873 (rbcL)	this study

Kryptoperidinium triquetrum (Ehrenb.) Tillmann, Gottschling, Elbr., Kusber & Hoppenrath	W1E12	Baltic Sea, off Germany: Mecklenburg-Vorpommern, Wismar (53°55'N, 11°26'E)	Sep 18, 2019	U. Tillmann, M. Gottschling & A. Kremp [U. Tillmann] s.n.	OP256593 (ITS), OP256612 (esSSU+esITS+esLSU)	this study
Kryptoperidinium triquetrum (Ehrenb.) Tillmann, Gottschling, Elbr., Kusber & Hoppenrath (epitype)	W4A06 (≡ CCAC9296B)	Baltic Sea, off Germany: Mecklenburg-Vorpommern, Wismar (53°55'N, 11°26'E)	Sep 18, 2019	U. Tillmann, M. Gottschling & A. Kremp [U. Tillmann] s.n.	OP256581 (ITS+LSU), OP256600 (esSSU+esITS), OP264870 (psbA), OP264871 (rbcL)	this study
Kryptoperidinium triquetrum (Ehrenb.) Tillmann, Gottschling, Elbr., Kusber & Hoppenrath	W4A07	Baltic Sea, off Germany: Mecklenburg-Vorpommern, Wismar (53°55'N, 11°26'E)	Sep 18, 2019	U. Tillmann, M. Gottschling & A. Kremp [U. Tillmann] s.n.	OP256589 (ITS), OP256611 (esSSU+esITS+esLSU)	this study
Kryptoperidinium triquetrum (Ehrenb.) Tillmann, Gottschling, Elbr., Kusber & Hoppenrath	W4A09	Baltic Sea, off Germany: Mecklenburg-Vorpommern, Wismar (53°55'N, 11°26'E)	Sep 18, 2019	U. Tillmann, M. Gottschling & A. Kremp [U. Tillmann] s.n.	OP256584 (ITS), OP256610 (esSSU+esITS+esLSU)	this study
Kryptoperidinium triquetrum (Ehrenb.) Tillmann, Gottschling, Elbr., Kusber & Hoppenrath	W4A10	Baltic Sea, off Germany: Mecklenburg-Vorpommern, Wismar (53°55'N, 11°26'E)	Sep 18, 2019	U. Tillmann, M. Gottschling & A. Kremp [U. Tillmann] s.n.	OP256590 (ITS), OP256605 (esSSU+esITS+esLSU)	this study
Kryptoperidinium triquetrum (Ehrenb.) Tillmann, Gottschling, Elbr., Kusber & Hoppenrath	W4B10	Baltic Sea, off Germany: Mecklenburg-Vorpommern, Wismar (53°55'N, 11°26'E)	Sep 18, 2019	U. Tillmann, M. Gottschling & A. Kremp [U. Tillmann] s.n.	OP256583 (ITS), OP256603 (esSSU+esITS+esLSU)	this study
Kryptoperidinium triquetrum (Ehrenb.) Tillmann, Gottschling, Elbr., Kusber & Hoppenrath	W4F01	Baltic Sea, off Germany: Mecklenburg-Vorpommern, Wismar (53°55'N, 11°26'E)	Sep 18, 2019	U. Tillmann, M. Gottschling & A. Kremp [U. Tillmann] s.n.	OP256587 (ITS), OP256602 (esSSU+esITS+esLSU)	this study
Kryptoperidinium triquetrum (Ehrenb.) Tillmann, Gottschling, Elbr., Kusber & Hoppenrath	W4F09	Baltic Sea, off Germany: Mecklenburg-Vorpommern, Wismar (53°55'N, 11°26'E)	Sep 18, 2019	U. Tillmann, M. Gottschling & A. Kremp [U. Tillmann] s.n.	OP256579 (SSU+ITS+LSU), OP256604 (esSSU+esITS+esLSU)	this study

Kryptoperidinium triquetrum (Ehrenb.) Tillmann, Gottschling, Elbr., Kusber & Hoppenrath	W20H6	Baltic Sea, off Germany: Mecklenburg-Vorpommern, Wismar (53°55'N, 11°26'E)	Sep 23, 2020	U. Tillmann [U. Tillmann] s.n.	OP256582 (ITS)	this study
Kryptoperidinium triquetrum (Ehrenb.) Tillmann, Gottschling, Elbr., Kusber & Hoppenrath	W20H7	Baltic Sea, off Germany: Mecklenburg-Vorpommern, Wismar (53°55'N, 11°26'E)	Sep 23, 2020	U. Tillmann [U. Tillmann] s.n.	OP256588 (ITS)	this study
Naiadinium polonicum (Wołosz.) Carty (reference material)	HBI:MG200823a	n.inf.	n.inf.	n.inf.	JQ639764 (SSU), JQ639772 (ITS), JQ639754 (LSU)	Zhang et al. (unpubl. 2012)
Nottbeckia ochracea (Levander) Gert Hansen, Daugbjerg & Moestrup (reference material)	GH957	Finland: Uusimaa, Raseborg, Tvärminne, Brännskär	Jun 19, 2011	n.inf.	MG754078 (SSU), MG754079 (LSU)	53
Palatinus apiculatus (Ehrenb.) Craveiro, Calado, Daugbjerg & Moestrup (epitype)	GeoM*762 (≡ CCAC6788B)	Germany: Berlin, Mitte, Tiergarten (52°31'N, 13°21'E)	Mar 28, 2016	M. Gottschling [J. Kretschmann] D047	KY996787 (SSU+ITS+LSU), MG255412 (LSUd8d10)	54
Parvodinium cf. *centenniale* (Playfair) Carty	GeoM*795 (≡ CCAC6789B)	Poland: Lesser Poland, Tatra, Toporowy Staw Niżni (49°17'N, 20°02'E)	Aug 4, 2016	J. Kretschmann & P.M. Owsianny [J. Kretschmann] PL069	MG255428 (SSU+ITS+LSU), MG255420 (LSUd8d10)	55
Parvodinium trawinskii Kretschmann, Owsianny, Zerdoner & Gottschling (holotype)	GeoM*753 (≡ CCAC6787B)	Poland: Lesser Poland, Tatra, Długi Staw Gąsienicowy (49°14'N, 20°01'E, 1784m)	Sep 22, 2015	P.M. Owsianny, K. Trawiński & G. Marciniak [J. Kretschmann] PL019	MG255427 (SSU+ITS+LSU), MG255419 (LSUd8d10)	55
Peridiniopsis borgei Lemmerm. (reference material)	PBSKA	Sweden: Skåne, St. Kalkbrottsdammen (55°31'N, 12°55'E)	2005	n.inf.	EF058241 (SSU), EU445295 (ITS), EF058261 (LSU), FJ236464 (LSU), EF417339 (cyb)	51, 56–57
"*Peridinium*" *americanum* Gran & Braarud	080218-12	western North Pacific, East China Sea, off Japan: Kyūshū, Nagasaki, Sasebo	n.inf.	n.inf.	AB716911 (SSU), AB716925 (LSU)	58
Peridinium bipes forma *globosum* Er.Lindem.	NIES495 (≡ LOND9)	Japan: Fukushima, Lake Onogawa	Jul 30, 1985	[T. Sawaguchi s.n.]	GU046392 (SSU+ITS+LSU)	59–60
Peridinium bipes forma *occultatum* (Er.Lindem.) M.Lefèvre	HYJA0310	eastern Indian Ocean, off South Korea: Juam	n.inf.	n.inf.	GU046390 (SSU+ITS+LSU)	59–60
Peridinium cinctum (O.F.Müll.) Ehrenb.	CCAC0102 (≡ M1576/1)	Germany: Lower Saxony, Wittmund, Spiekeroog	1998	[D. Hille s.n.]	EF058244 (SSU), KF751925 (SSU+ITS+LSU), MF423370 (LSUd8d10)	4, 8, 16, 51, 61

Peridinium willei Huitf.-Kaas	TK007	Japan: Hokkaidō, Kiritappu-shitsugen	n.inf.	n.inf.	AB232669 (SSU+ITS+LSU)	62
†*Pernambugia tuberosa* (Kamptner) Janofske & Karwath (reference material)	GeoB*61 (≡ CCAC4752B, CCAP1141/1)	western South Atlantic (11°32'S, 28°35'W, −100m)	Feb 27, 1997	[Meteor 38/1] [M. Kirsch] 4321-9	KR362907 (SSU), JN982372 (ITS+LSU), MF423371 (LSUd8d10)	4, 8, 16, 31, 61
Pfiesteria piscicida Steid. & J.M.Burkh.	n.inf.	USA–MD: Chicamacomico River	1997	K.A. Steidinger & J.M. Burkholder s.n.	AY112746 (rRNA)	63
†*Posoniella tricarinelloides* (G.Versteegh) Streng, Banasová, Reháková & H.Willems (reference material)	PTLY01	western North Pacific, Yellow Sea, off China: Jiangsu, Lianyungang (34°49'N, 119°32E, –15m)	May 9, 2011	H. Gu s.n.	KC511790 (SSU), KC511792 (ITS), KC511794 (LSU)	64
Preperidinium meunieri (Pavill.) Elbr.	cell1_3clones	western North Atlantic, off USA–MA: Salt Pond	n.inf.	n.inf.	EF152930 (LSU)	65
Protoperidinium bipes (Paulsen) Balech	n.inf.	western North Pacific, Sea of Japan, off Japan: Hokkaidō, Ishikari	n.inf.	n.inf.	AB284159 (SSU), AB284160 (LSU)	66
Protoperidinium pellucidum Bergh	#28	western North Pacific, Sea of Japan, off Japan: Hokkaidō, Shiribeshi, Otaru (43°10'N, 141°01'E)	Oct 31, 2002	n.inf.	AB181903 (SSU), AB255862 (LSU)	67–68
Scrippsiella bicarinata Zinssmeister, S.Soehner, S.Meier & Gottschling	GeoB 411 (≡ CCAC5106B, CCCM324)	Mediterranean Sea, Tyrrhenian Sea, off Italy: Formia (41°15'N, 13°36'E)	Apr, 2009	M. Gottschling, C. Zinßmeister, S. Söhner [M. Kirsch] ITA00044	MH497031 (SSU), KF751927 (SSU+ITS+LSU)	8, 16, 55
"*Scrippsiella*" *donghaienis* H.Gu	CS168	eastern Indian Ocean, off Australia (33°S, 138°E)	1983	J.L. Stauber s.n.	JN982374 (ITS+LSU), MH497032 (LSUd8d10), JN982415 (cyb), GQ501314 (coI)	4, 36, 69
Scrippsiella sweeneyae Balech	CCCM280	n.inf.	n.inf.	A. Chan s.n.	HQ845331 (SSU+ITS+LSU), EU840175 (cyb)	4–5, 32
Theleodinium calcisporum Craveiro, Pandeirada, Daugbjerg, Moestrup & Calado (holotype)	MP69	Portugal: Aveiro, Ílhavo, Gafanha da Boavista (40°36'N, 8°42'W)	Mar 2, 2011	n.inf.	KC699492 (SSU+ITS+LSU)	70
Thoracosphaera heimii (Lohmann) Kamptner	CCCM670 (≡ NCMA1069)	western North Atlantic, Gulf of Mexico	Apr 23, 1980	L. Brand s.n.	HQ845327 (SSU+ITS+LSU)	32, 45

Tintinnophagus acutus Coats (isolated from *Tintinnopsis cylindrica* Daday, 1887) (holotype)	n.inf.	western North Atlantic, off USA–MD: Rhode River, Chesapeake Bay (38°53'N, 76°33'W)	n.inf.	n.inf.	HM483397 (SSU+ITS+LSU)	21
Unruhdinium jiulongense (H.Gu) Gottschling (holotype)	PSJL01	China: Fujian, Zhangzhou, Jiulongjiang River, Xipi (25°07'N, 117°32'E)	Dec, 2012	n.inf.	KM217384 (SSU), KY594913 (ITS), KY594912 (LSU), KM217383 (esSSU)	71–72
Unruhdinium cf. *kevei* (Grigorszky & Vagas) Gottschling	DA08	Japan: Hokkaidō, Shikotsu Lake	n.inf.	n.inf.	LC054935 (SSU)	18
Unruhdinium minimum (Qi Zhang, G.X.Liu & Z.Y.Hu) Gottschling (holotype)	QZ-2012 HBI:FJ201101	China: Fujian, Zhangzhou, Jiulongjiang River	n.inf.	n.inf.	JQ639767 (SSU), JQ639770 (ITS), JQ639752 (LSU)	73
Unruhdinium niei (G.X.Liu & Z.Y.Hu) Gottschling (holotype)	Donghu	China: Hubei, Wuhan, Donghu Lake (30°33'N, 114°23'E)	Mar 18, 2004	G.-X. Liu s.n.	HM596542 (SSU), HM596550 (ITS), HM596555 (LSU)	74
Unruhdinium penardii (Lemmerm.) Gottschling (reference material)	Jiulongjiang	China: Fujian, Zhangzhou, Jiulongjiang River (24°35'N, 117°41'E)	Feb, 2009	n.inf.	HM596543 (SSU), HM596551 (ITS), HM596556 (LSU), HM596547 (esSSU)	74
Unruhdinium penardii (Lemmerm.) Gottschling	n.inf.	Japan: Shiga, Lake Biwa	Mar 30, 2005	n.inf.	AB353771 (SSU), AB353773 (esSSU), AB353775 (rbcL)	75
Unruhdinium penardii var. *robustum* (Qi Zhang, G.X.Liu & Z.Y.Hu) Gottschling (holotype)	Manwan	China: Yunnan, Manwan, Luodi River (24°44'N, 100°21'E)	Apr 14, 2008	G.-X. Liu s.n.	HM596554 (ITS), HM596558 (LSU), HM596549 (esSSU)	74
Vulcanodinium rugosum Nézan & Chomérat	G	France	n.inf.	n.inf.	MG826115 (SSU), MG826367 (ITS), MG826107 (LSU)	76
Zooxanthella nutricula K.Brandt (isolated from *Thalassicolla nucleata* Huxley, 1851)	BBSR323	western North Atlantic, Sargasso Sea, off UK, the Bermudas: 3-5 miles SE of Bermuda	n.nf.	n.nf.	U52356 (SSU), KC511788 (ITS+LSU)	77–78

Table S2: Primer sets used for PCR amplification and sequencing.

Target	Primer	Direction	Sequence 5´–3´	Reference
Host LSU (D1–D2)	D1R	forward	ACC CGC TGA ATT TAA GCA TA	79
Host LSU (D1–D2)	D2C	reverse	CCT TGG TCC GTG TTT CAA GA	79
Host LSU (D3)	28_14838	forward	GCT ACT ACC ACC AAG ATC TGC	80
Host LSU (D3)	28S_2	reverse	TGA AAA GGA CTT TAA AAG AGA	79
Host ITS	ITS1	forward	TCC GTA GGT GAA CCT GCG G	81
Host ITS	ITS4	reverse	TCC TCC GCT TAT TGA TAT GC	82
Host ITS	ITSA	forward	CCA AGC TTC TAG ATC GTA ACA AGG (ACT)TC CGT AGG T	83
Host ITS	ITSB	reverse	CCT GCA GTC GAC A(GT)A TGC TTA A(AG)T TCA GC(AG) GG	83
Host SSU	1F	forward	AAC CTG GTT GAT CCT GCC AGT	84–85
Host SSU	528F	forward	GCG GTA ATT CCA GCT CCA A	84–85
Host SSU	1055F	forward	GGT GGT GCA TGG CCG TTC TT	84–85
Host SSU	536R	reverse	AAT TAC CGC GGC (GT)GC TGG CA	84–85
Host SSU	1055R	reverse	ACG GCC ATG CAC CAC CAC CCA T	84–85
Host SSU	1528R	reverse	TGA TCC TTC TGC AGG TTC ACC TAC	84–85
Endosymbiont ITS	Ns7m	forward	GGC AAT AAC AGG TCT GT	86
Endosymbiont ITS	LR1850	reverse	CCT CAC GGT ACT TGT TC	86
Endosymbiont ITS (nested)	SR12cf	forward	TAG AGG AAG GAG AAG TCG TAA	62
Endosymbiont ITS (nested)	25F1R	reverse	ATA TGC TTA AAT TCA GCG G	62
Endosymbiont ITS (nested)	1645F	forward	CTT ATC ATT TAG AGG AAG GTG AAG TCG	28
Endosymbiont ITS (nested)	28SR	reverse	CCG CTT CAC TCG CCG TTA CT	28

Endosymbiont SSU	EukA	forward	ACC TGG TTG ATC CTG CCA GT	85
Endosymbiont SSU	EukB	reverse	TGA TCC TTC TGC AGG TTC ACC TAC	85
Endosymbiont SSU (nested)	D1800R	reverse	GCT TGA TCC TTC TGC AGG T	28
Endosymbiont SSU (nested)	KrypendoSSU	forward	TTG CGC ACC AAG GTA ATG ATT AA	this study
Endosymbiont SSU	DiaR	reverse	CCA TTC AAT CGG TAG GTG C	74
Endosymbiont SSU	1055R	reverse	ACG GCC ATG CAC CAC CAC CCA T	87
Endosymbiont SSU	1010R	reverse	ACG GTA TCT GAT CGT CTT CGA TCC C	this study
Endosymbiont SSU	930F	forward	AAG ACG GAC TAC TGC GAA AGC A	this study
Endosymbiont SSU	1055F	forward	GGT GGT GCA TGG CCG TTC TT	84
Endosymbiont SSU	1209F	forward	CAG GTC TGT GAT GCC C	88

Table S3: Primer combinations and PCR conditions.

Target	rRNA region	Primer	Cycling Conditions		Cycles
**Host**	LSU D1–D2	D1R	Initial denaturation:	94°C / 120 sec	30 x
		D2C	Denaturation:	94°C / 30 sec	
			Annealing:	55°C / 30 sec	
		(=Sequencing primers)	Elongation:	68°C / 120 sec	
			Final extension:	68°C / 600 sec	
			Hold:	10°C	
**Host**	LSU D3	28_14838	Initial denaturation:	95°C / 180 sec	30 x
		28S_2	Denaturation:	95°C / 30 sec	
			Annealing:	52°C / 60 sec	
		(=Sequencing primers)	Elongation:	68°C / 60 sec	
			Final extension:	68°C / 600 sec	
			Hold:	10°C	
**Host**	ITS	ITS1	Initial denaturation:	94°C / 240 sec	10 x
		ITS4	Denaturation:	94°C / 50 sec	
			Annealing:	58°C / 40 sec	
		ITSA	Elongation:	70°C / 60 sec	
		ITSB	Denaturation:	94°C / 45 sec	30 x
			Annealing:	50°C / 45 sec	
		(=Sequencing primers)	Elongation:	70°C / 60 sec	
			Final extension:	70°C / 300 sec	
			Hold:	10°C	
**Host**	SSU	1F	Initial denaturation:	94°C / 240 sec	30 x
		1528R	Denaturation:	94°C / 120 sec	
			Annealing:	55°C / 120 sec	
			Elongation:	68°C / 180 sec	
			Final extension:	68°C / 600 sec	
			Hold:	10°C	

**Host**	SSU	1F<br>528F<br>1055F<br>536R<br>1055R<br>1528R	Initial denaturation:<br>Denaturation:<br>Annealing:<br>Elongation:<br>Hold:	96°C / 60 sec<br>96°C / 10 sec<br>50°C / 5 sec<br>60°C / 240 sec<br>10°C	25 x
**Endosymbiont**	ITS	Ns7m<br>LR1850	Initial denaturation:<br>Denaturation:<br>Annealing:<br>Elongation:<br>Final extension:<br>Hold:	94°C / 300 sec<br>94°C / 50 sec<br>52°C / 60 sec<br>72°C / 90 sec<br>72°C / 600 sec<br>10°C	32 x
**Endosymbiont**	Nested in ITS product	SR12cf<br>25F1R<br><br>(=Sequencing primers A)	Initial denaturation:<br>Denaturation:<br>Annealing:<br>Elongation:<br>Final extension:<br>Hold:	94°C / 120 sec<br>94°C / 35 sec<br>50°C / 35 sec<br>72°C / 60 sec<br>72°C / 300 sec<br>10°C	39 x
**Endosymbiont**	Nested in ITS product	1645F<br>28SR<br><br>(=Sequencing primers B)	Initial denaturation:<br>Denaturation:<br>Annealing:<br>Elongation:<br>Final extension:<br>Hold:	95°C / 120 sec<br>95°C / 10 sec<br>67°C / 30 sec<br>72°C / 90 sec<br>72°C / 600 sec<br>10°C	34 x

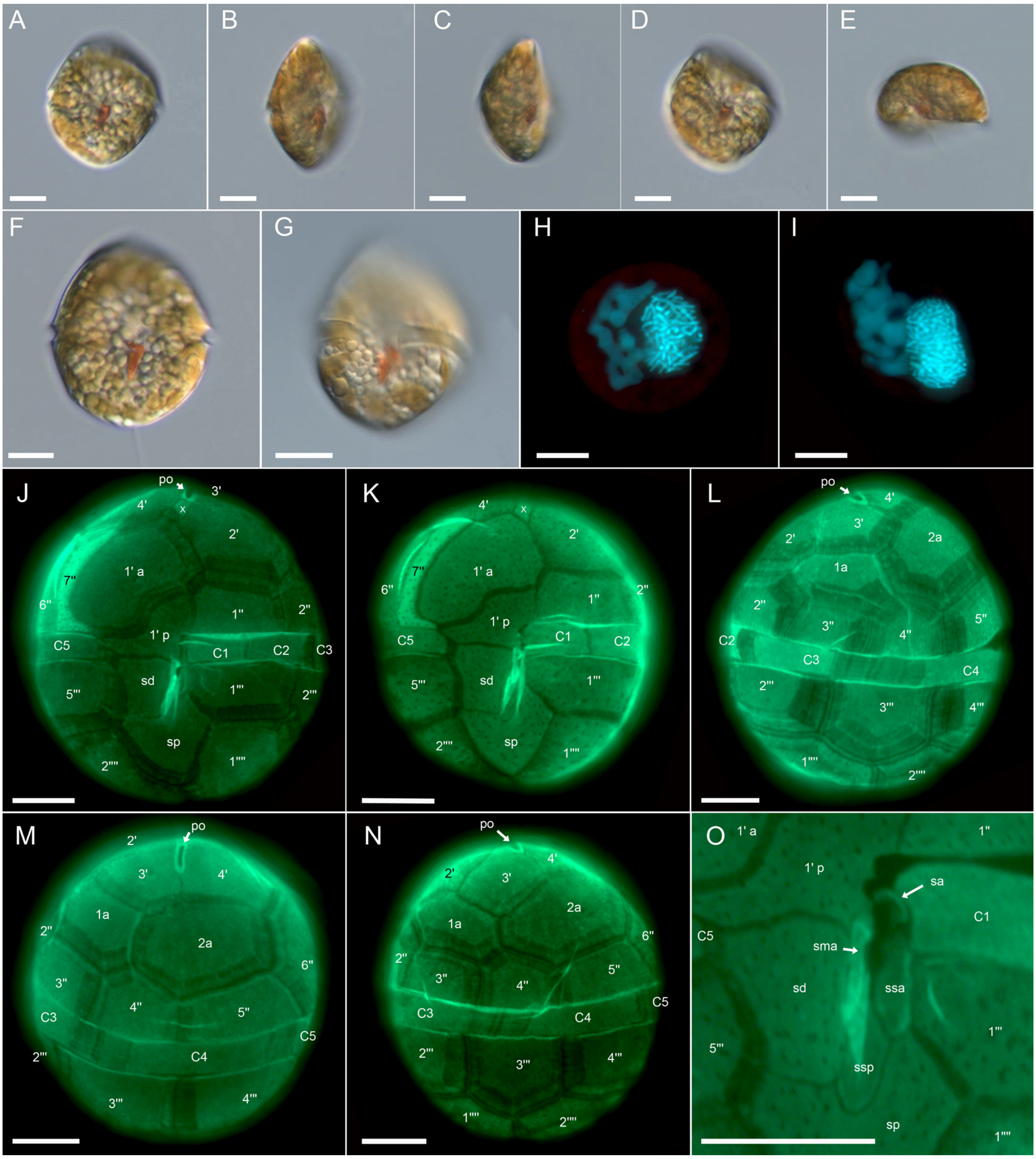

Figure S1: ***Kryptoperidinium triquetrum*, strain W4-A10**. Light microscopy of living cells (A–G), formaldehyde-fixed cells (H–I) or lugol-fixed cells (J–O). (A–E) The same cells in ventral (A), lateral (B–C), ventral-antapical (D) and antapical view (E). (J–O) Light microscopy of cells stained with solophenyl flavine and viewed with epifluorescence and green light excitation. (J–K) Cells in ventral view. (L) Cell in left-lateral view. (M–N) Cells in dorsal view. (O) Detailed view of the sulcal area with sulcal plates. Plate labels according to the Kofoidean system, modified by labelling an anterior part (1′ a) and a posterior part (1′ p) of the first apical plate. Sulcal plate labels: sa = anterior sulcal plate; sd = right sulcal plate; sma = anterior median sulcal plate; smp = posterior median sulcal plate; sp = posterior sulcal plate; ssa = anterior left sulcal plate; ssp = posterior left sulcal plate. Scale bars = 10 µm.

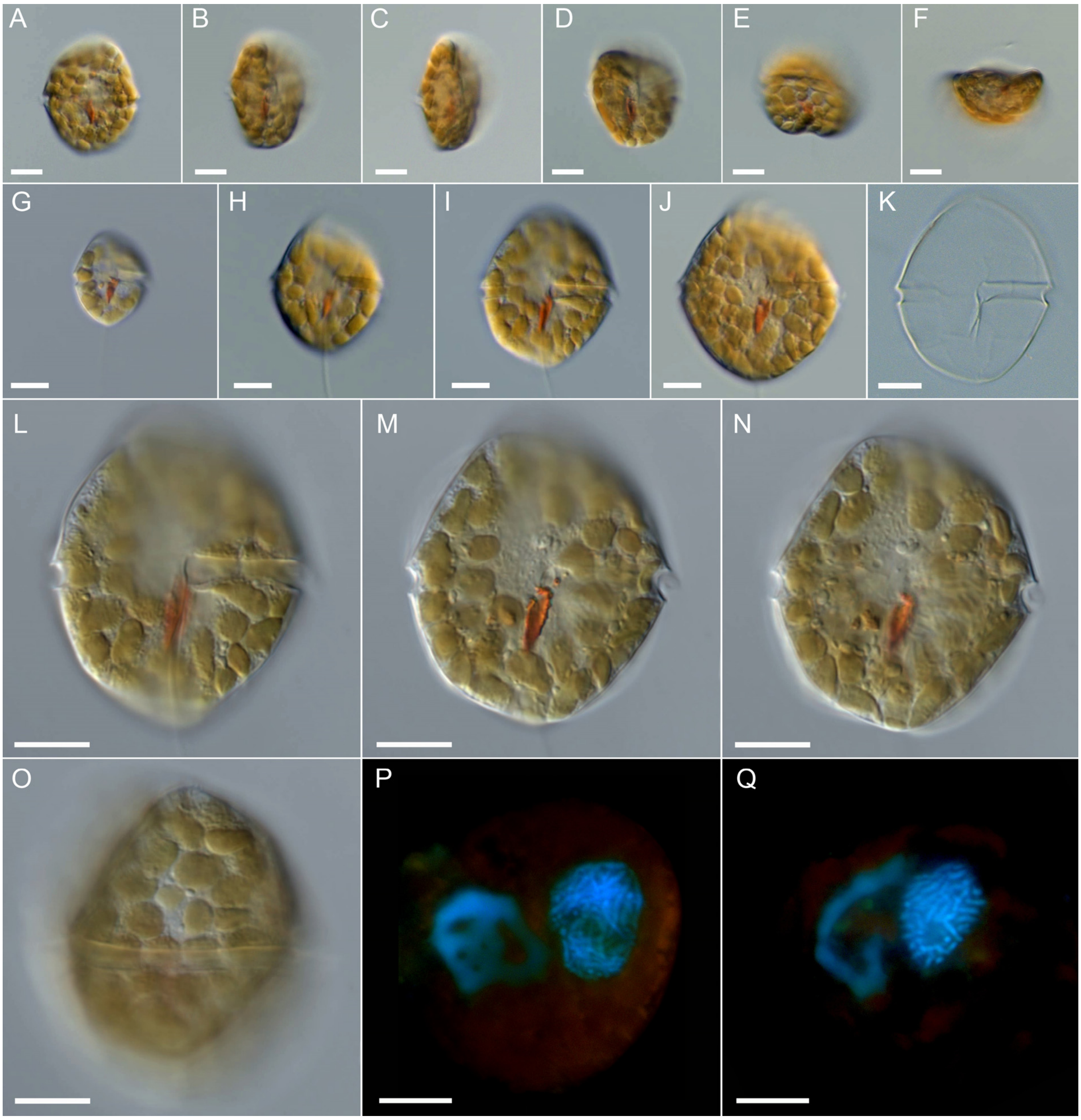

Figure S2: ***Kryptoperidinium triquetrum*, strain W4-F9**. Light microscopy of living cells (A–O) or formaldehyde-fixed cells (P–Q). (A–F) The same cell in ventral (A), lateral (B–C), ventral-antapical (D), dorsal-antapical (E) and antapical view (F). (G–J) Cells of different size in ventral view. (K) Empty theca in ventral view. (L–O) The same cell in ventral view in different focal planes (L–M) or in dorsal view (O). (P–Q) Different cells stained with DAPI and viewed with epifluorescence and UV excitation, note the irregularly shaped diatom nucleus (left) and the dinophyte nucleus with condensed chromosomes (right). Scale bars = 10 µm.

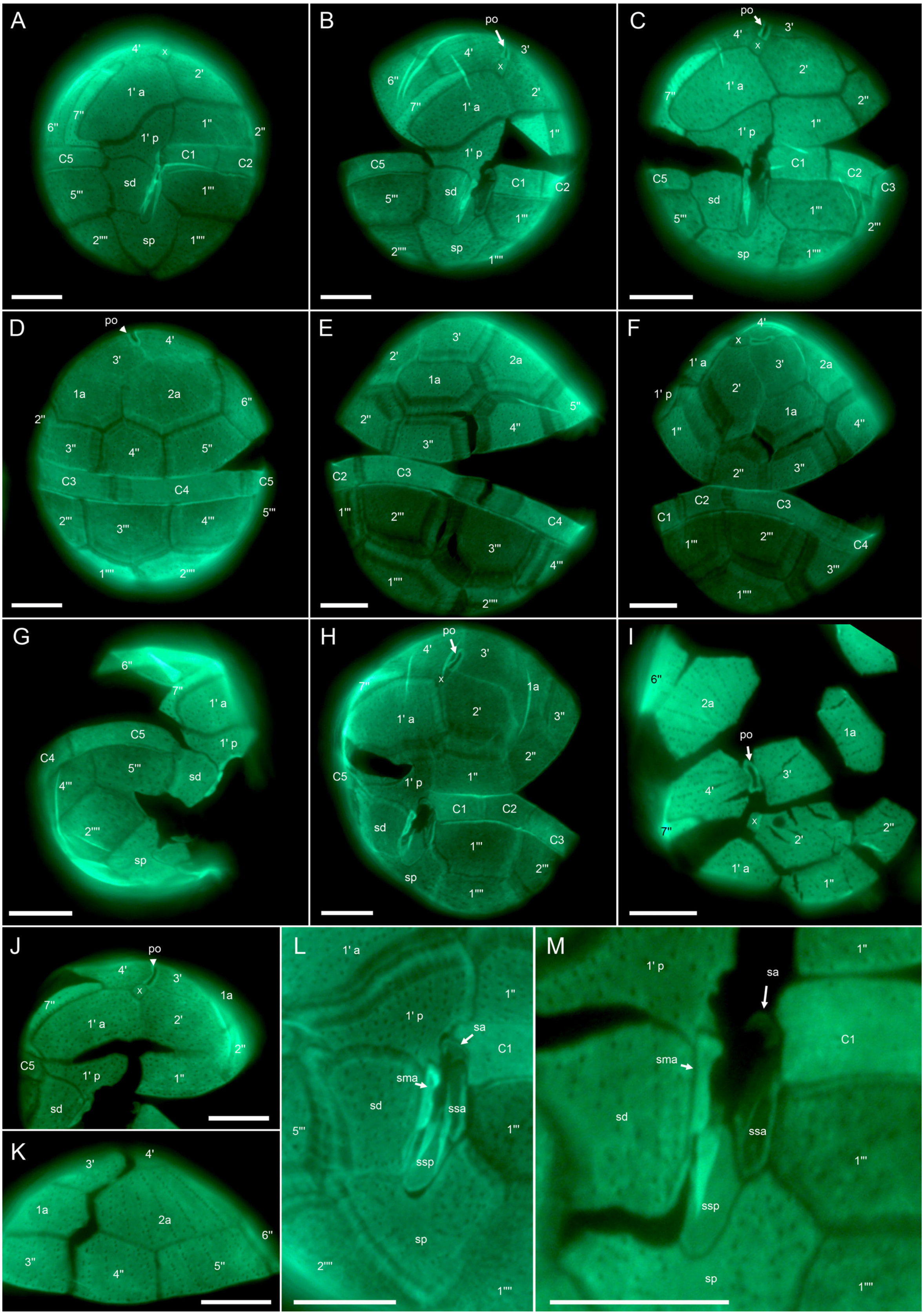

Figure S3: ***Kryptoperidinium triquetrum*****, strain W4-F9**. Light microscopy of lugol-fixed cells stained with solophenyl flavine and viewed with epifluorescence and green light excitation. (A–C) Cells in ventral view. (D–E) Cells in dorsal view. (F) Cell in left-lateral view. (G) Cell in right-lateral view. (H) Cell in ventral apical view. (I–J) Detailed view of epithecal plates in apical view. (K) Epitheca in dorsal view, note the arrangement of thecal pores in lines. (L–M) Detailed view of the sulcal area with sulcal plates. Plate labels according to the Kofoidean system, modified by labelling an anterior part (1′ a) and a posterior part (1′ p) of the first apical plate. Sulcal plate labels: sa = anterior sulcal plate; sd = right sulcal plate; sma = anterior median sulcal plate; smp = posterior median sulcal plate; sp = posterior sulcal plate; ssa = anterior left sulcal plate; ssp = posterior left sulcal plate. Scale bars = 10 µm.

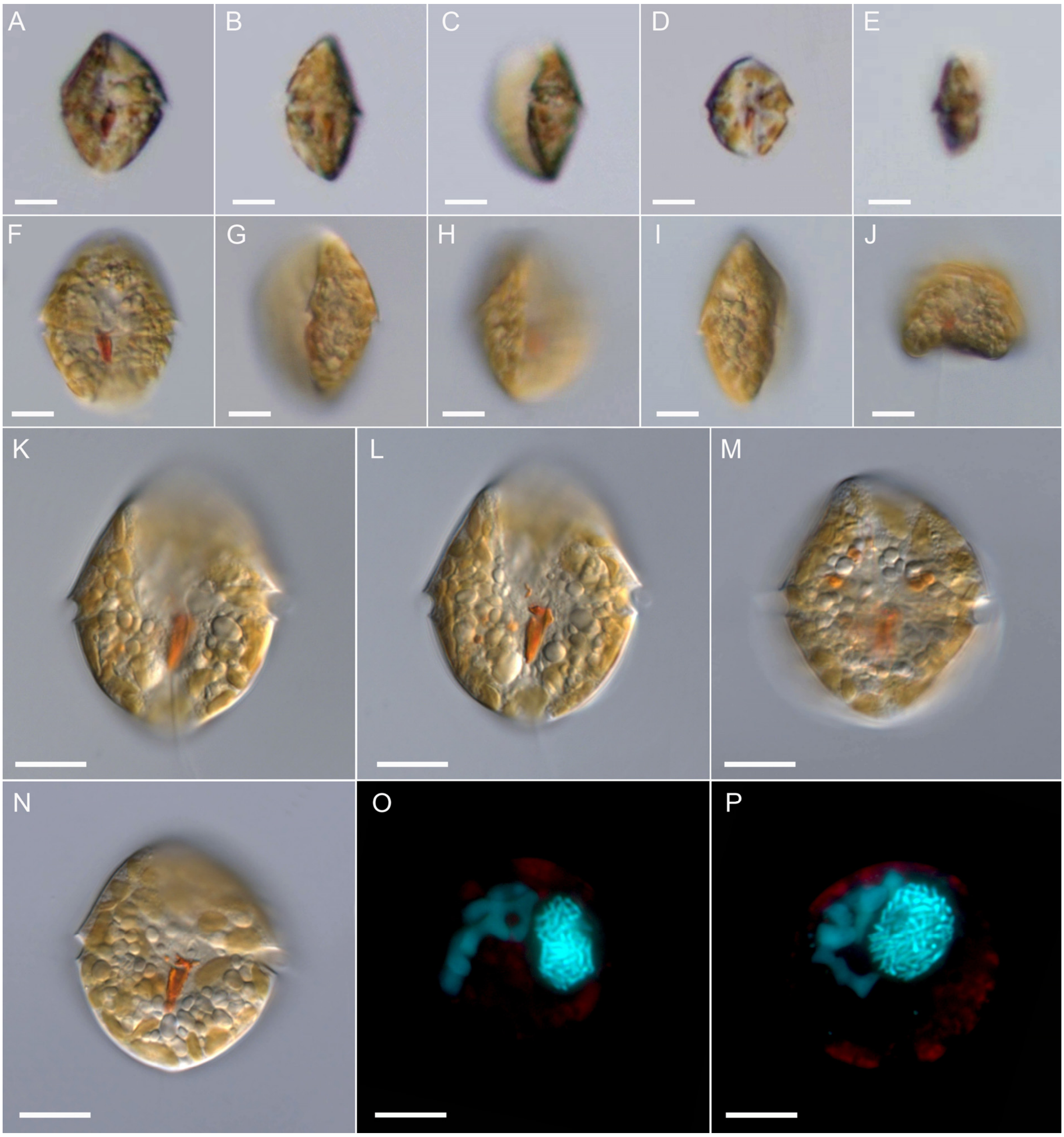

Figure S4: ***Kryptoperidinium triquetrum*, strain W1-E4**. Light microscopy of living cells (A–N) or formaldehyde-fixed cells (O–P). (A–C) The same cell in ventral (A) and lateral view (B–C). (D–E) The same cell in ventral (D) and lateral view (E). (F–J) The same cell in ventral (F), left-lateral (G), right-lateral (H–I) and antapical view (J). (K–L) The same cell in ventral view in different focal planes. (M–N) Cells in ventral view. (O–P) Different cells stained with DAPI and viewed with epifluorescence and UV excitation, note the irregularly shaped diatom nucleus (left) and the dinophyte nucleus with condensed chromosomes (right). Scale bars = 10 µm.

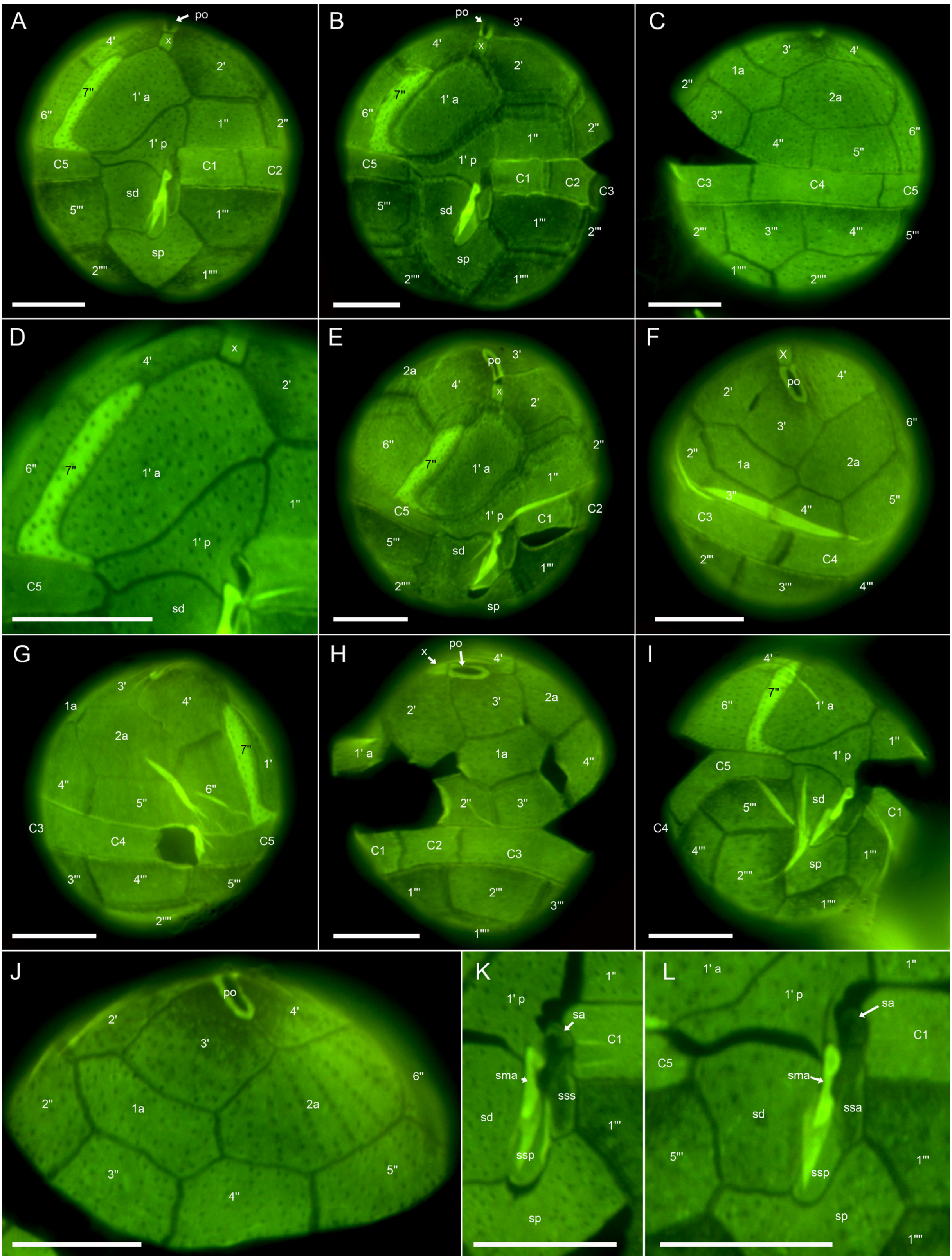

Figure S5: ***Kryptoperidinium triquetrum*, strain W1-E4**. Light microscopy of lugol-fixed cells stained with solophenyl flavine and viewed with epifluorescence and green light excitation. (A–B) Cells in ventral view. (C) Cell in dorsal view. (D) Detailed ventral view of plate 7′′. (E) Cell in ventral apical view. (F) Cell in dorsal apical view. (G) Cell in right-lateral view. (H) Cell in left-lateral view. (I) Cell in ventral antapical view. (J) Epitheca in dorsal view, note the arrangement of thecal pores in lines. (K–L) Detailed view of the sulcal area with sulcal plates. Plate labels according to the Kofoidean system, modified by labelling an anterior part (1′ a) and a posterior part (1′ p) of the first apical plate. Sulcal plate labels: sa = anterior sulcal plate; sd = right sulcal plate; sma = anterior median sulcal plate; smp = posterior median sulcal plate; sp = posterior sulcal plate; ssa = anterior left sulcal plate; ssp = posterior left sulcal plate. Scale bars = 10 µm.

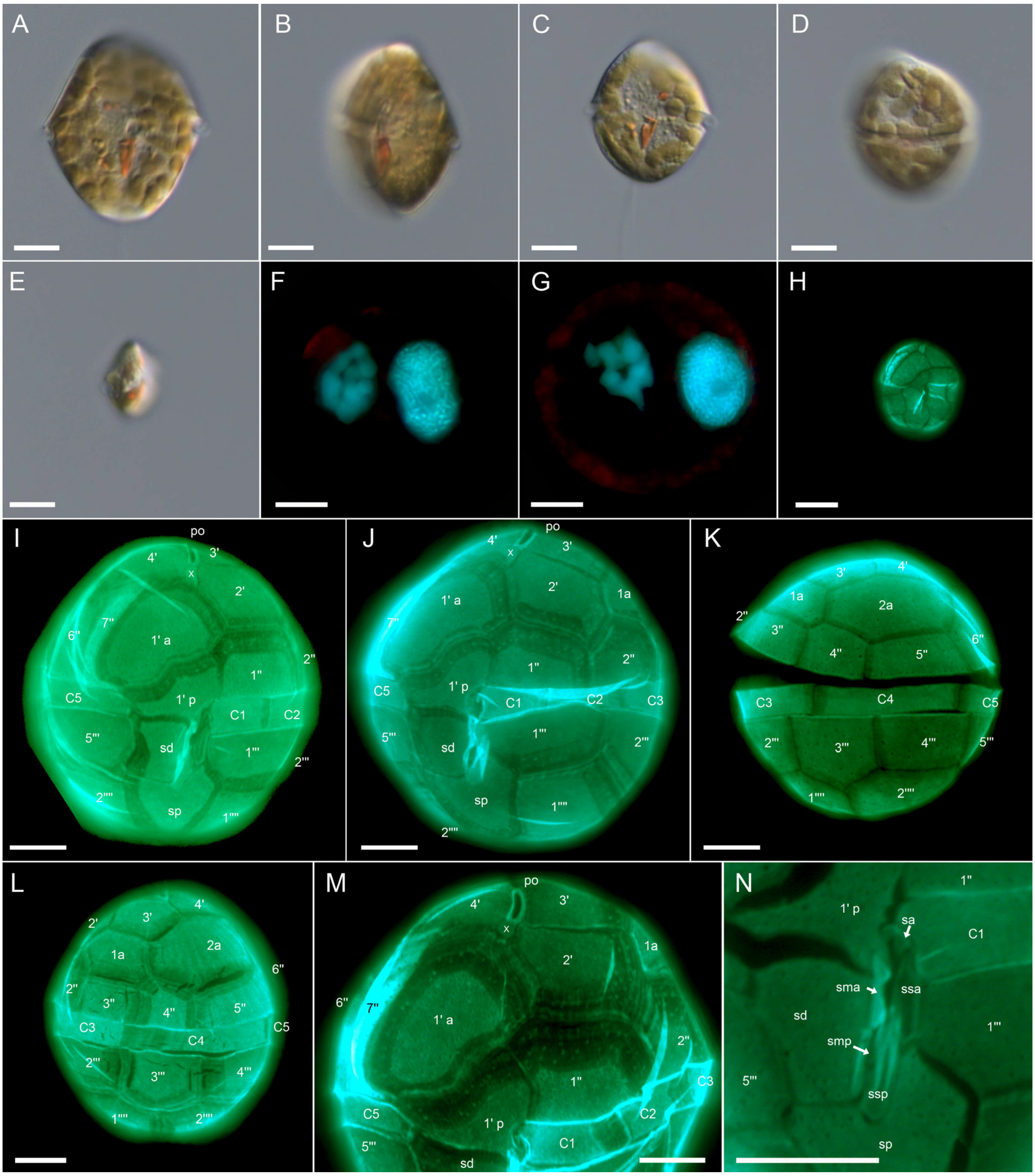

Figure S6: *Kryptoperidinium triquetrum*, strain W1-D6. Light microscopy of living cells (A–E), formaldehyde-fixed cells (F–G) or lugol-fixed cells (H–N). (A–B) The same cell in ventral (A) or lateral view (B). (C–D) The same cells in ventral (C) or dorsal view (D). (E) A small cell in lateral view. (F–G) Different cells stained with DAPI and viewed with epifluorescence and UV excitation, note the irregularly shaped diatom nucleus (left) and the dinophyte nucleus with condensed chromosomes (right). (H–N) Light microscopy of cells stained with solophenyl flavine and viewed with epifluorescence and green light excitation. (H) Small cell in ventral view. (I–J) Cells in ventral view. (K–L) Cells in dorsal view. (M) Epithecal plates in ventral apical view. (N) Detailed view of the sulcal area with sulcal plates. Plate labels according to the Kofoidean system, modified by labelling an anterior part (1′ a) and a posterior part (1′ p) of the first apical plate. Sulcal plate labels: sa = anterior sulcal plate; sd = right sulcal plate; sma = anterior median sulcal plate; smp = posterior median sulcal plate; sp = posterior sulcal plate; ssa = anterior left sulcal plate; ssp = posterior left sulcal plate. Scale bars = 10 µm.

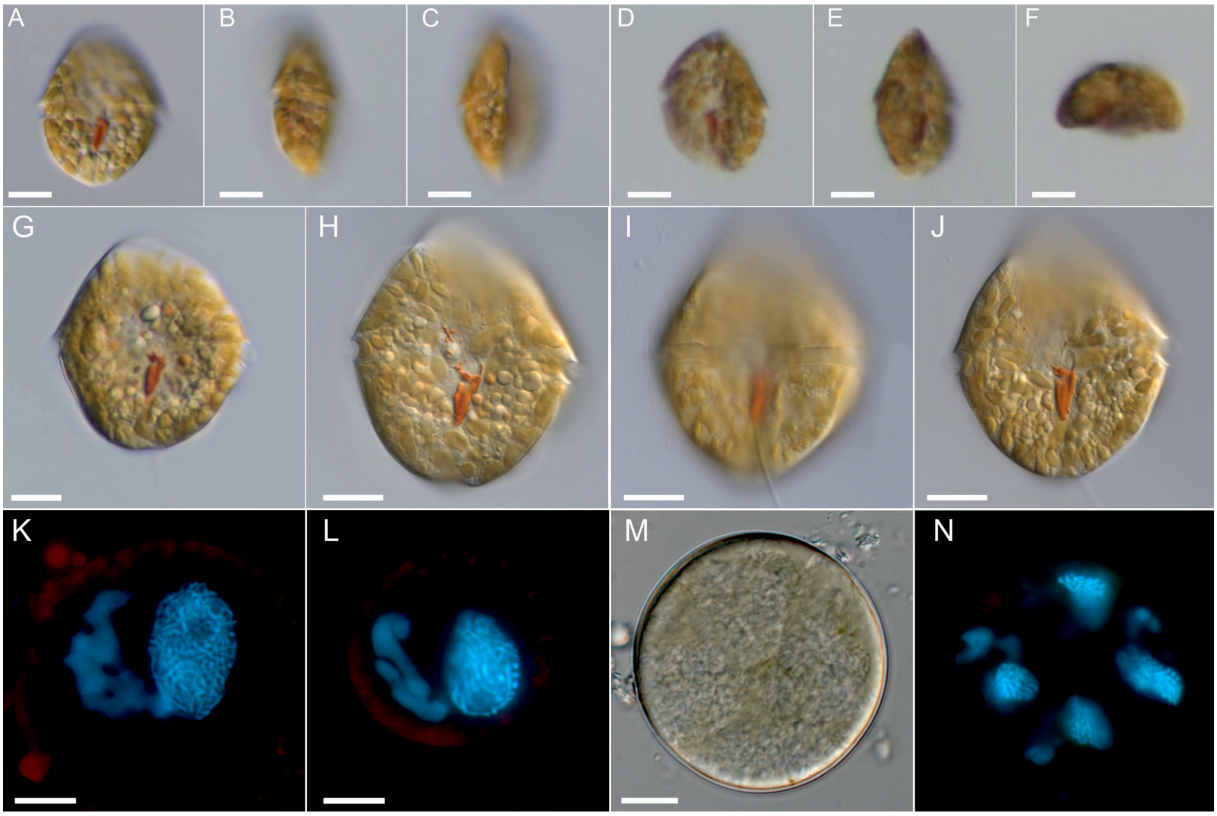

Figure S7: ***Kryptoperidinium triquetrum*, strain W1-D11**. Light microscopy of living cells (A–J) or formaldehyde-fixed cells (K–N). (A–C) The same cell in ventral (A) and lateral view (B–C). (D–F) The same cell in ventral lateral (D), lateral (E) or antapical (F) view. (G–H) Cells in ventral view. (I–J) The same cell in ventral view in two different focal planes. (K–L) Different cells stained with DAPI and viewed with epifluorescence and UV excitation, note the irregularly shaped diatom nucleus (left) and the dinophyte nucleus with condensed chromosomes (right). (M–N) A DAPI-stained squeezed (flat) 4-cell coccoid division stage in LM (M) and epifluorescence and UV excitation (N). Scale bars = 10 µm.

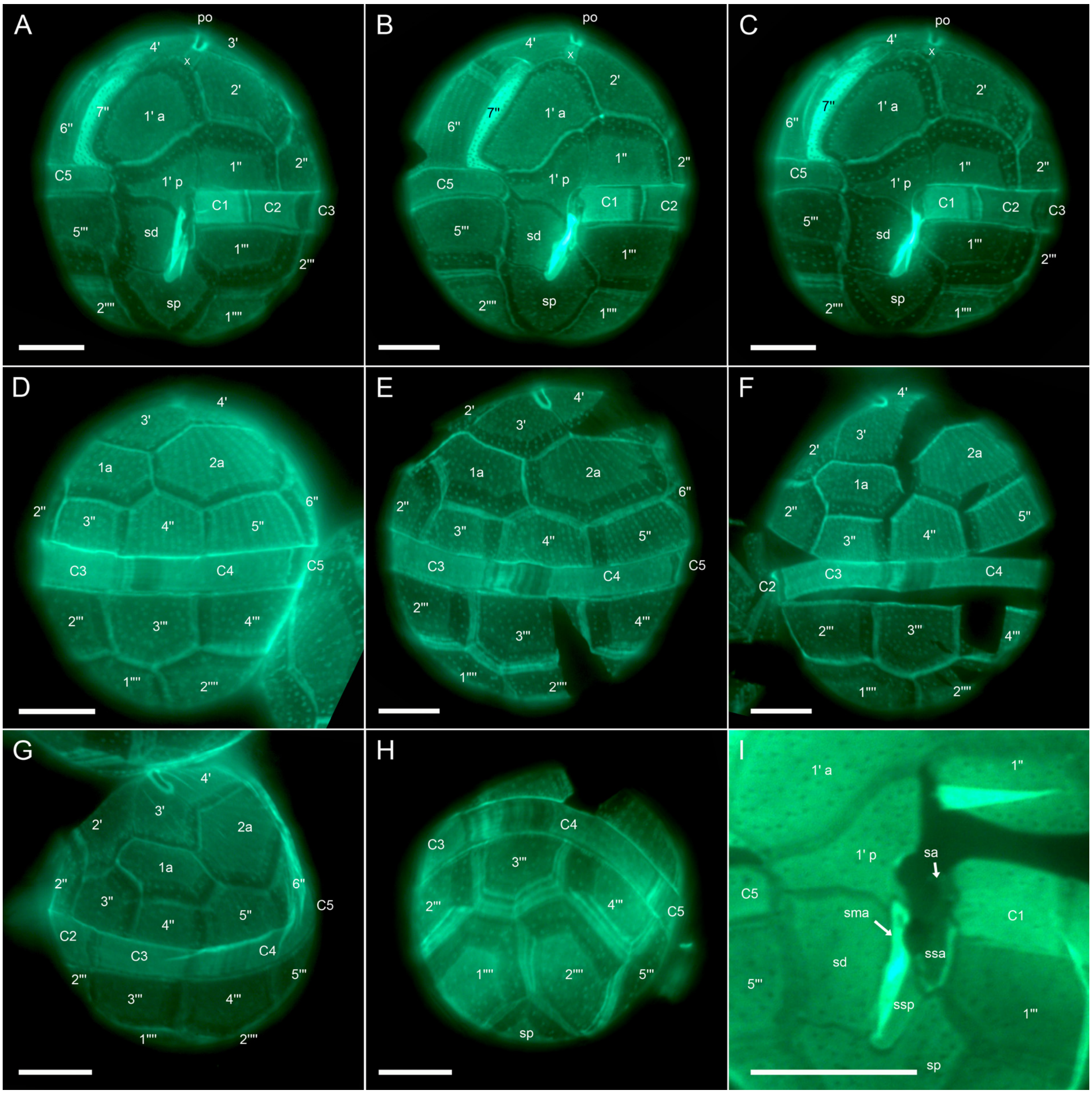

Figure S8: ***Kryptoperidinium triquetrum*, strain W1-D11**. Light microscopy of lugol-fixed cells stained with solophenyl flavine and viewed with epifluorescence and green light excitation. (A–C) Cells in ventral view. (D–E) Cells in dorsal view. (F–G) Cells in left-lateral view. (H) Cell in dorsal antapical view. (I) Detailed view of the sulcal area with sulcal plates. Plate labels according to the Kofoidean system, modified by labelling an anterior part (1′ a) and a posterior part (1′ p) of the first apical plate. Sulcal plate labels: sa = anterior sulcal plate; sd = right sulcal plate; sma = anterior median sulcal plate; smp = posterior median sulcal plate; sp = posterior sulcal plate; ssa = anterior left sulcal plate; ssp = posterior left sulcal plate. Scale bars = 10 µm.

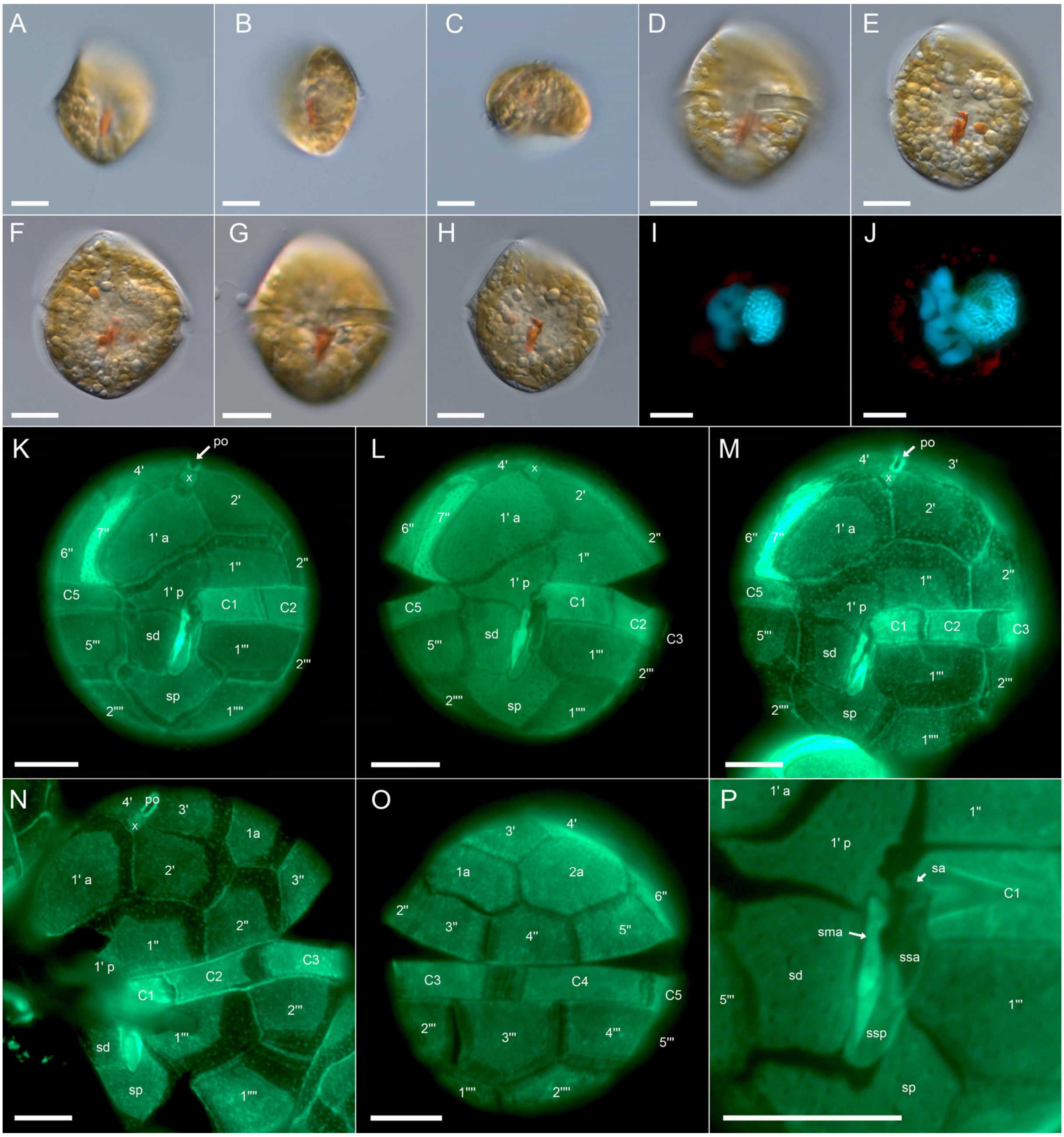

Figure S9: ***Kryptoperidinium triquetrum*, strain G-E8**. Light microscopy of living cells (A–H), formaldehyde-fixed cells (I–J) or lugol-fixed cells (K–P). (A–C) The same cell in ventral-lateral (A), lateral (B) and antapical view (C). (D–E) The same cell in ventral view with two different focal planes. (F–H) Cells in ventral view. (I–J) Different cells stained with DAPI and viewed with epifluorescence and UV excitation, note the irregularly shaped diatom nucleus (left) and the dinophyte nucleus with condensed chromosomes (right). (K–P) Light microscopy of cells stained with solophenyl flavine and viewed with epifluorescence and green light excitation. (K–M) Cells in ventral view. (N) Cell in left-lateral view. (O) Cell in dorsal view. (P) Detailed view of the sulcal area with sulcal plates. Plate labels according to the Kofoidean system, modified by labelling an anterior part (1′ a) and a posterior part (1′ p) of the first apical plate. Sulcal plate labels: sa = anterior sulcal plate; sd = right sulcal plate; sma = anterior median sulcal plate; smp = posterior median sulcal plate; sp = posterior sulcal plate; ssa = anterior left sulcal plate; ssp = posterior left sulcal plate. Scale bars = 10 µm.

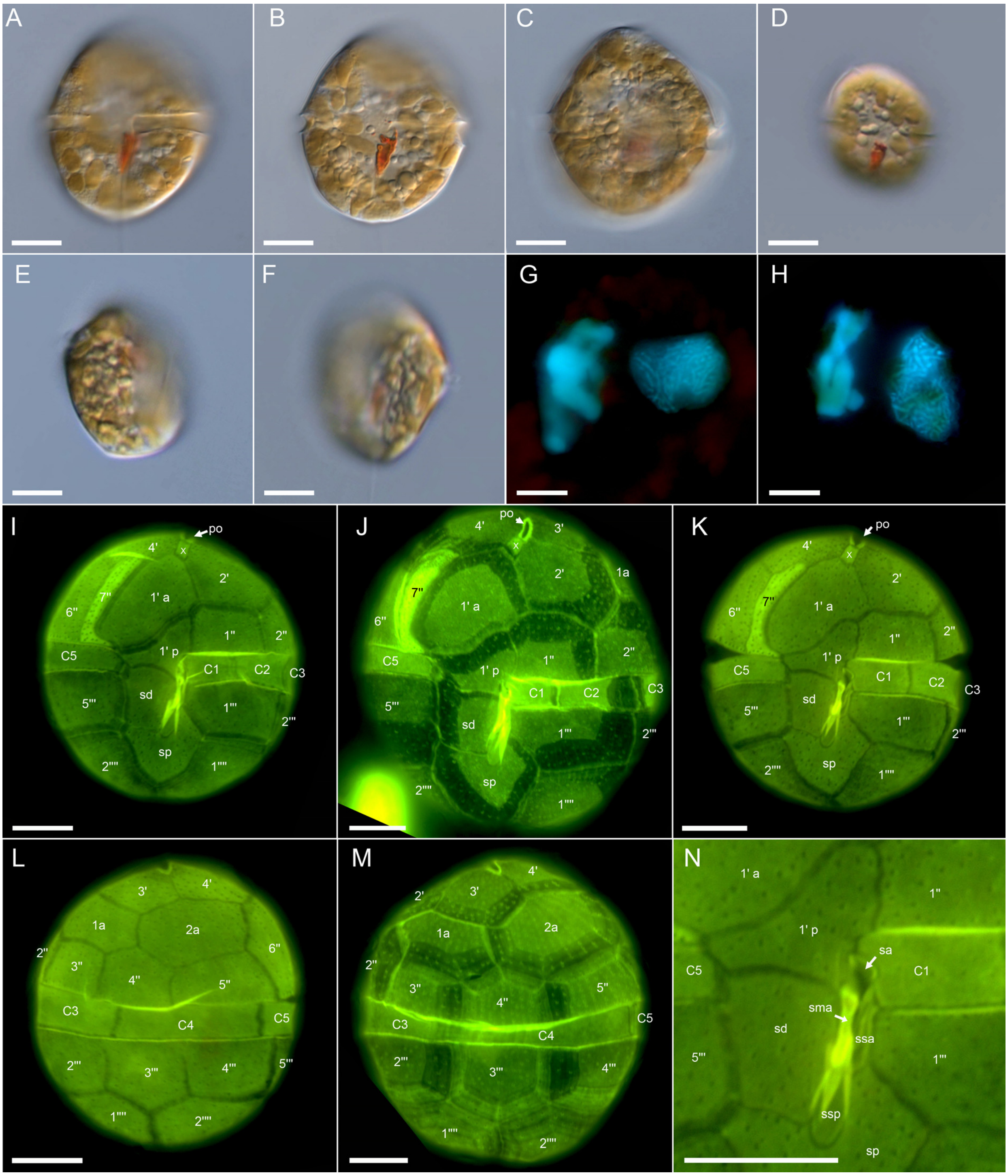

Figure S10: ***Kryptoperidinium triquetrum*, strain G-F9**. Light microscopy of living cells (A–F), formaldehyde-fixed cells (G–H) or lugol-fixed cells (I–N). (A–C) The same cell in ventral view with different focal planes. (D–F) Different cells in ventral (D) or lateral view (E–F). (G–H) Different cells stained with DAPI and viewed with epifluorescence and UV excitation, note the irregularly shaped diatom nucleus (left) and the dinophyte nucleus with condensed chromosomes (right). (I–N) Light microscopy of cells stained with solophenyl flavine and viewed with epifluorescence and green light excitation. (I–K) Cells in ventral view. (L–M) Cells in dorsal view. (N) Detailed view of the sulcal area with sulcal plates. Plate labels according to the Kofoidean system, modified by labelling an anterior part (1′ a) and a posterior part (1′ p) of the first apical plate. Sulcal plate labels: sa = anterior sulcal plate; sd = right sulcal plate; sma = anterior median sulcal plate; smp = posterior median sulcal plate; sp = posterior sulcal plate; ssa = anterior left sulcal plate; ssp = posterior left sulcal plate. Scale bars = 10 µm.

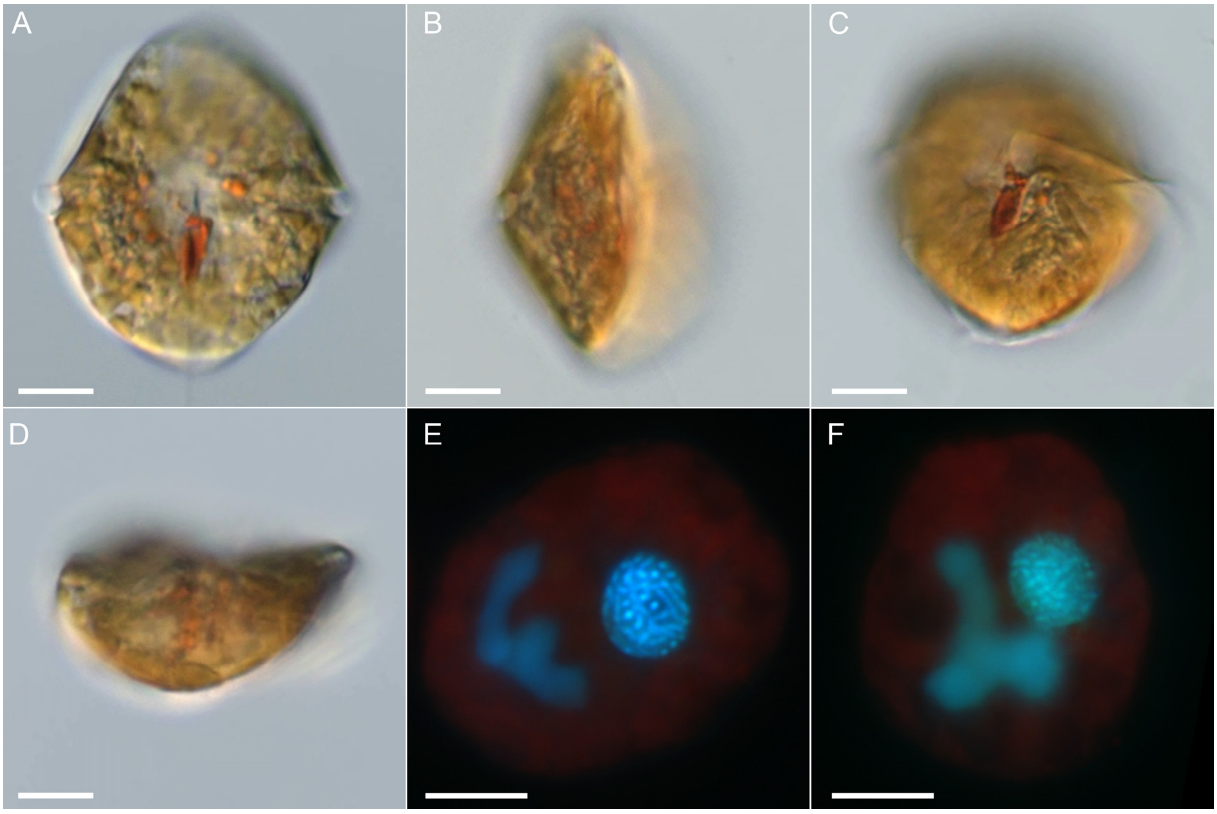

Figure S11: ***Kryptoperidinium triquetrum*, strain KFF 0901**. Light microscopy of living cells (A–D) or formaldehyde-fixed cells (E–F). (A–B) The same cells in ventral (A) and lateral view (B). (C–D) Different cells in ventral antapical (C) or antapical view (D). (E–F) Different cells stained with DAPI and viewed with epifluorescence and UV excitation, note the irregularly shaped diatom nucleus (left) and the dinophyte nucleus with condensed chromosomes (right). Scale bars = 10 µm.

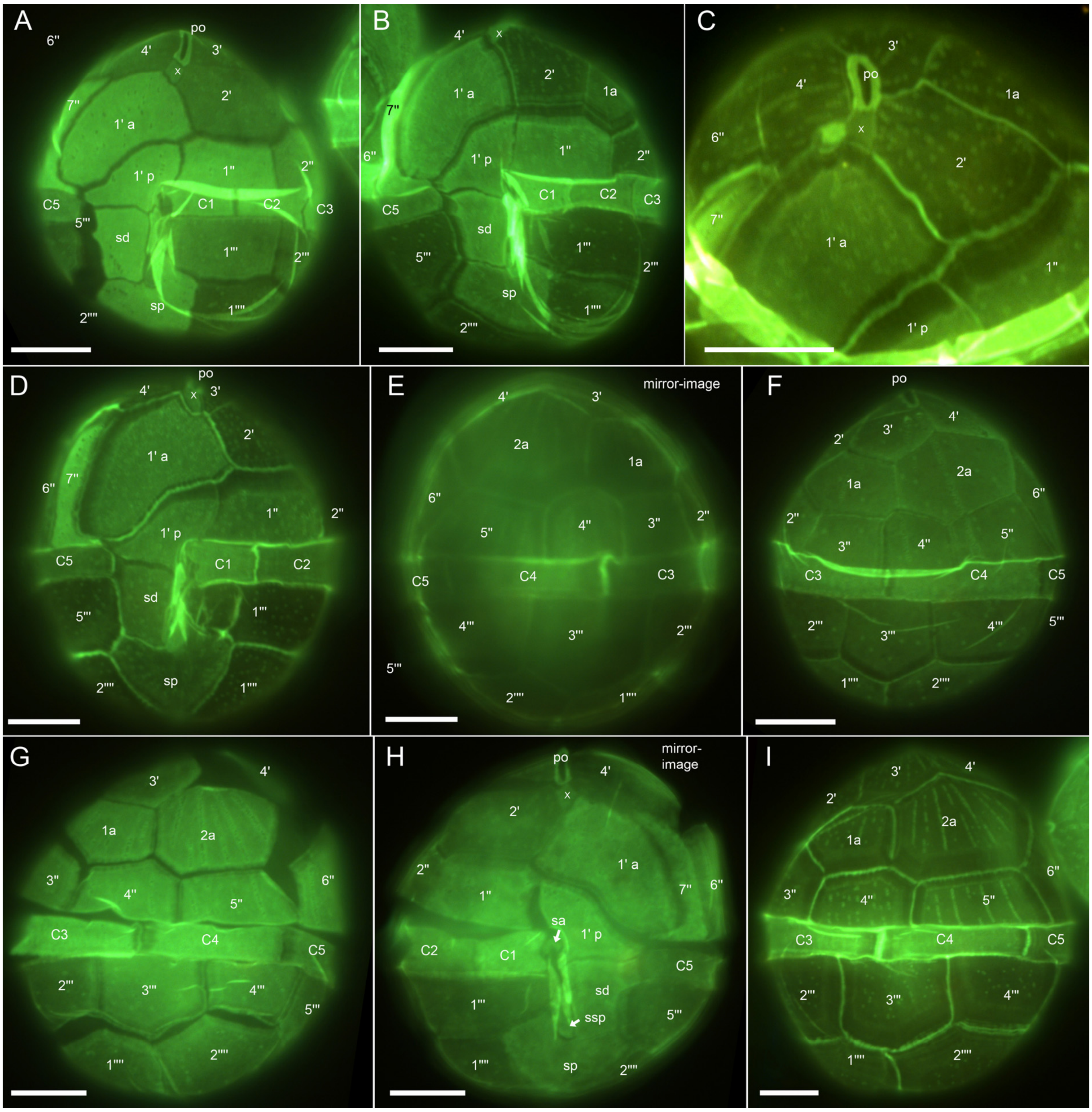

Figure S12: ***Kryptoperidinium triquetrum*, strain KFF 0901**. Light microscopy of lugol-fixed cells stained with solophenyl flavine and viewed with epifluorescence and green light excitation. (A–B) Cells in ventral view. (C) Detailed view of epithecal plates in apical view. (D–E) The same cell with focus on ventral (D) or dorsal plates (E), note that plate arrangement in E appears as mirror-imaged. (F) Cell in dorsal view. (G–H) The same cell with focus on dorsal (G) or ventral plates (H), note that plate arrangement in H appears as mirror-imaged. (I) Cell in dorsal view. Plate labels according to the Kofoidean system, modified by labelling an anterior part (1′ a) and a posterior part (1′ p) of the first apical plate. Sulcal plate labels: sa = anterior sulcal plate; sd = right sulcal plate; sma = anterior median sulcal plate; smp = posterior median sulcal plate; sp = posterior sulcal plate; ssa = anterior left sulcal plate; ssp = posterior left sulcal plate. Scale bars = 10 µm.

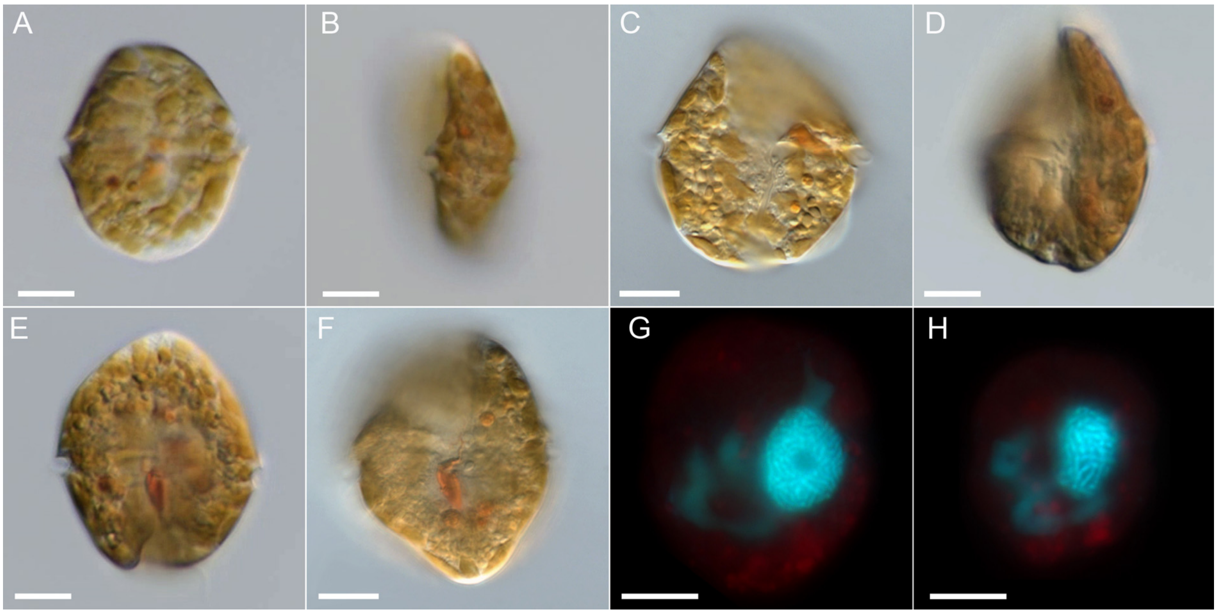

Figure S13: ***Kryptoperidinium triquetrum*, strain KFF 1001**. Light microscopy of living cells (A–F) or formaldehyde-fixed cells (G–H). (A–B) The same cell in ventral (A) and lateral view (B). (C–F) Different cells in ventral (C), lateral (D), ventral (E) and ventral lateral view (F). (G–H) Different cells stained with DAPI and viewed with epifluorescence and UV excitation, note the irregularly shaped diatom nucleus (left) and the dinophyte nucleus with condensed chromosomes (right). Scale bars = 10 µm.

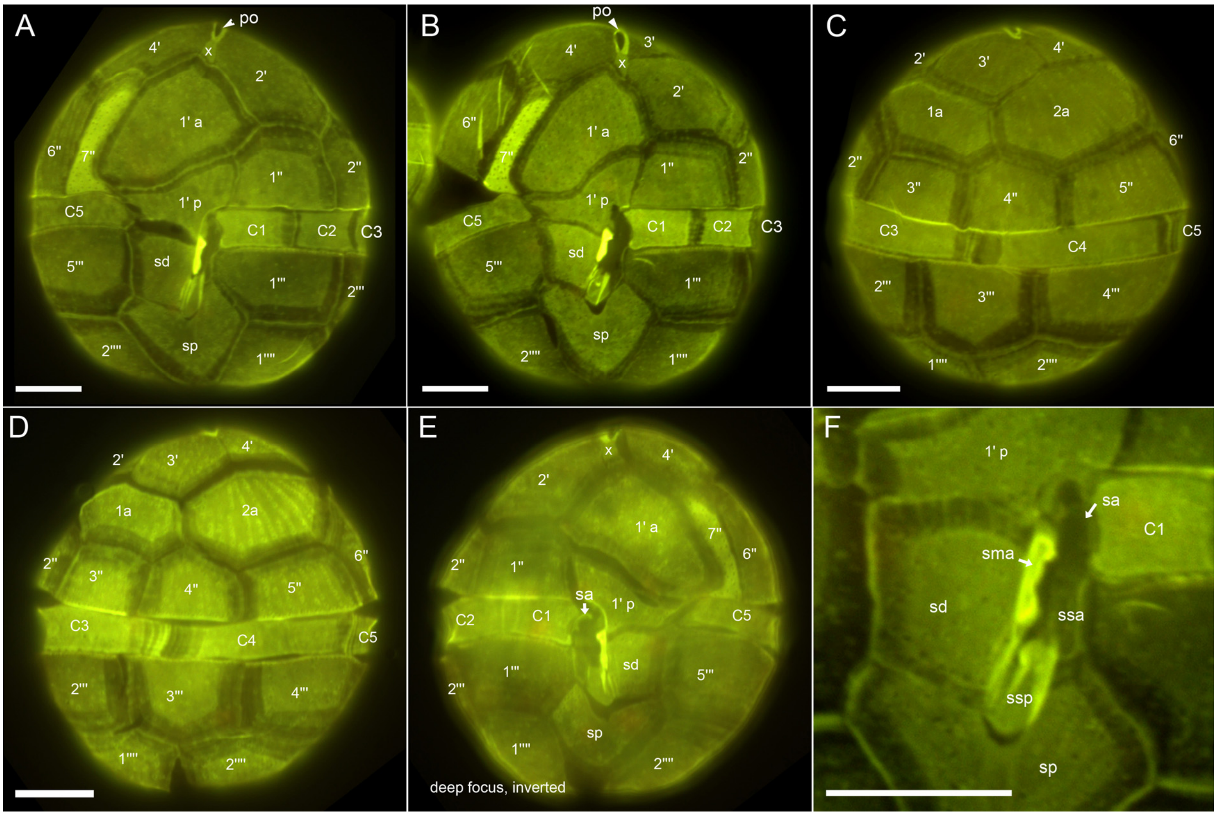

Figure S14: ***Kryptoperidinium triquetrum*, strain KFF 1001**. Light microscopy of lugol-fixed cells stained with solophenyl flavine and viewed with epifluorescence and green light excitation. (A–B) Cells in ventral view. (C) Cell in dorsal view. (D–E) The same cell with focus on dorsal (D) or ventral plates (E), note that plate arrangement in E appears as mirror-imaged. (F) Detailed view of the sulcal area with sulcal plates. Plate labels according to the Kofoidean system, modified by labelling an anterior part (1′ a) and a posterior part (1′ p) of the first apical plate. Sulcal plate labels: sa = anterior sulcal plate; sd = right sulcal plate; sma = anterior median sulcal plate; smp = posterior median sulcal plate; sp = posterior sulcal plate; ssa = anterior left sulcal plate; ssp = posterior left sulcal plate. Scale bars = 10 µm.

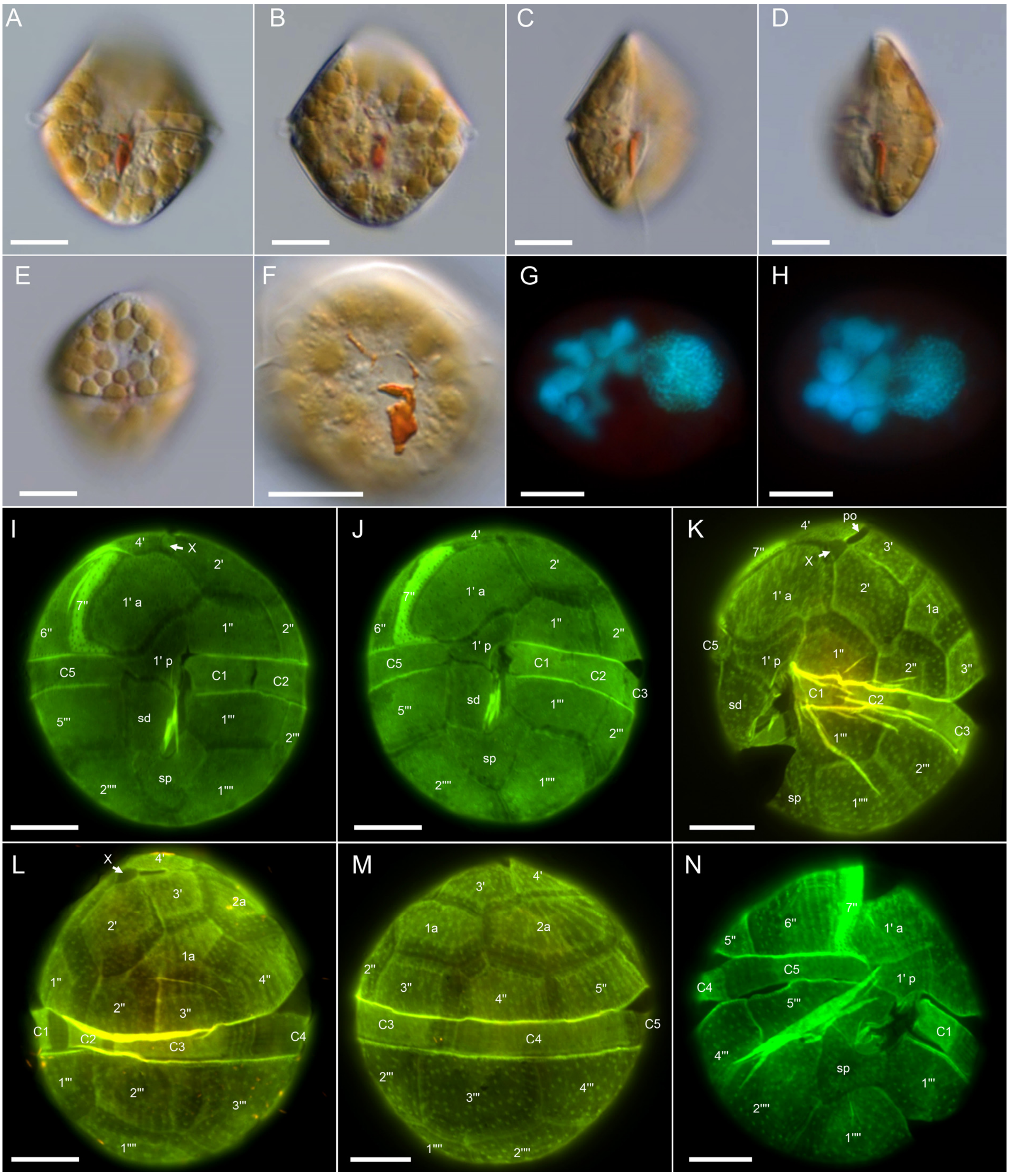

Figure S15: ***Kryptoperidinium triquetrum*, strain VGO 556**. Light microscopy of living cells (A–F), formaldehyde-fixed cells (G–H) or lugol-fixed cells (I–N). (A–E) Different cells in ventral (A–B), lateral (C–D) and dorsal view (E). (F) Detailed view of the stigma. (G–H) Different cells stained with DAPI and viewed with epifluorescence and UV excitation, note the irregularly shaped diatom nucleus (left) and the dinophyte nucleus with condensed chromosomes (right). (I–N) Light microscopy of cells stained with solophenyl flavine and viewed with epifluorescence and green light excitation. (I–K) Cells in ventral view. (L) Cell in left-lateral view. (M) Cell in dorsal view. (N) Cell in ventral antapical view. Plate labels according to the Kofoidean system, modified by labelling an anterior part (1′ a) and a posterior part (1′ p) of the first apical plate. Sulcal plate labels: sa = anterior sulcal plate; sd = right sulcal plate; sma = anterior median sulcal plate; smp = posterior median sulcal plate; sp = posterior sulcal plate; ssa = anterior left sulcal plate; ssp = posterior left sulcal plate. Scale bars = 10 µm.

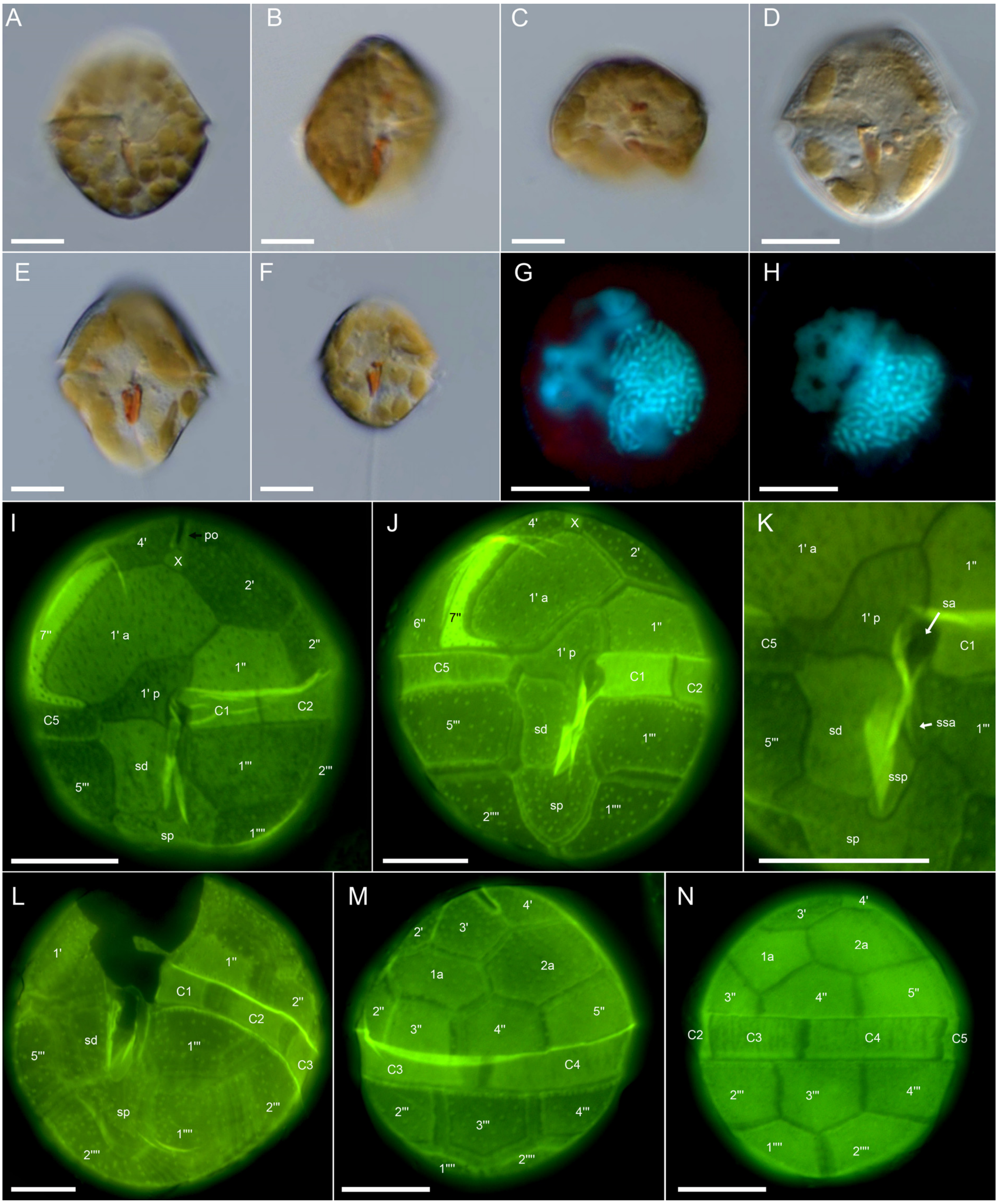

Figure S16: ***Kryptoperidinium triquetrum*, strain VGO 1124**. Light microscopy of living cells (A–F), formaldehyde-fixed cells (G–H) or lugol-fixed cells (I–N). (A–F) Different cells in ventral (A, D–F), lateral (B) and apical view (C). (G–H) Different cells stained with DAPI and viewed with epifluorescence and UV excitation, note the irregularly shaped diatom nucleus (left) and the dinophyte nucleus with condensed chromosomes (right). (I–N) Light microscopy of cells stained with solophenyl flavine and viewed with epifluorescence and green light excitation. (I–J) Cells in ventral view. (K) Detailed view of the sulcal area with sulcal plates. (L) Cell in ventral antapical view. (M–N) Cells in dorsal view. Plate labels according to the Kofoidean system, modified by labelling an anterior part (1′ a) and a posterior part (1′ p) of the first apical plate. Sulcal plate labels: sa = anterior sulcal plate; sd = right sulcal plate; sma = anterior median sulcal plate; smp = posterior median sulcal plate; sp = posterior sulcal plate; ssa = anterior left sulcal plate; ssp = posterior left sulcal plate. Scale bars = 10 µm.

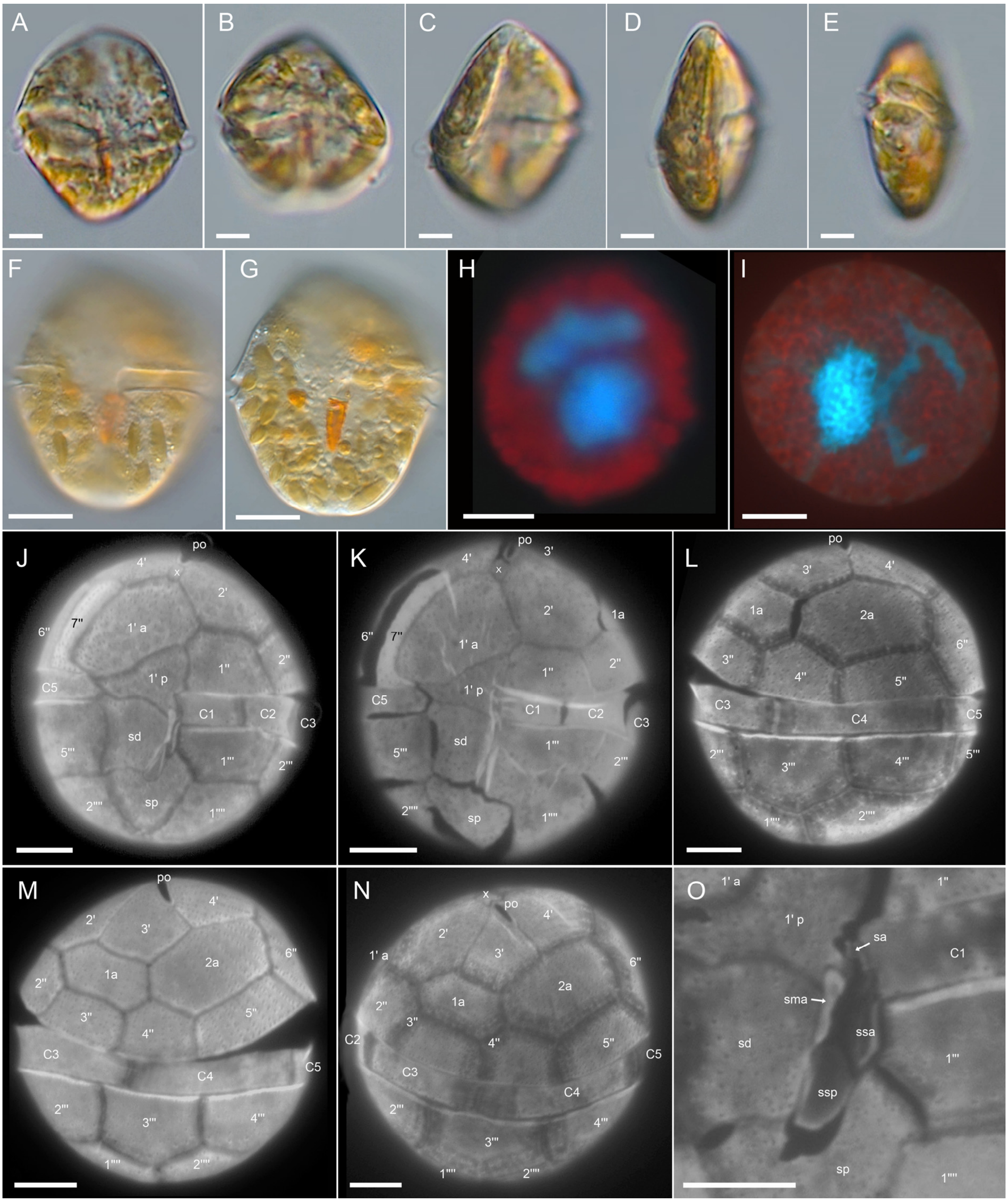

Figure 17: ***Kryptoperidinium triquetrum*, strain GeoB 459**. Light microscopy of living cells (A–G) or formaldehyde-fixed cells (H–I). (A–E) The same cell in ventral (A), ventral antapical (B), ventral right-lateral (C), right-lateral (D) and left-lateral view (E). (F–G) The same cell in ventral view in different focal planes. (H–I) Different cells stained with DAPI and viewed with epifluorescence and UV excitation, note the irregularly shaped diatom nucleus (left) and the dinophyte nucleus with condensed chromosomes (right). (J–O) Light microscopy of cells stained with calcofluor white and viewed with epifluorescence and UV light excitation. (J–K) Cells in ventral view. (L–N) Cells in dorsal view. (O) Detailed view of the sulcal area with sulcal plates. Plate labels according to the Kofoidean system, modified by labelling an anterior part (1′ a) and a posterior part (1′ p) of the first apical plate. Sulcal plate labels: sa = anterior sulcal plate; sd = right sulcal plate; sma = anterior median sulcal plate; smp = posterior median sulcal plate; sp = posterior sulcal plate; ssa = anterior left sulcal plate; ssp = posterior left sulcal plate. Scale bars = 10 µm.

References

1 Brummitt, R. K. & Powell, C. E. *Authors of plant names: A list of authors of scientific names of plants, with recommended standard forms of their names, including abbreviations*. (Royal Botanic Gardens, 1992).

2 Kang, N. S. *et al.* *Aduncodinium* gen. nov and *A. glandula* comb. nov (Dinophyceae, Pfiesteriaceae), from coastal waters off Korea: Morphology and molecular characterization. *Harmful Algae* **41**, 25–37 (2015).

3 Yamaguchi, A., Yoshimatsu, S., Hoppenrath, M., Wakeman, K. C. & Kawai, H. Molecular phylogeny of the benthic dinoflagellate genus *Amphidiniopsis* and its relationships with the family Protoperidiniaceae. *Protist* **167**, 568–583 (2016).

4 Gottschling, M. *et al.* Phylogeny of calcareous dinoflagellates as inferred from ITS and ribosomal sequence data. *Molecular Phylogenetics and Evolution* **36**, 444–455 (2005).

5 Kremp, A., Elbrächter, M., Schweikert, M., Wolny, J. L. & Gottschling, M. *Woloszynskia halophila* (Biecheler) comb. nov.: A bloom-forming cold-water dinoflagellate co-occurring with *Scrippsiella hangoei* (Dinophyceae) in the Baltic Sea. *Journal of Phycology* **41**, 629–642 (2005).

6 Logares, R. *et al.* Phenotypically different microalgal morphospecies with identical ribosomal DNA: A case of rapid adaptive evolution? *Microbial Ecology* **53**, 549–561 (2007).

7 Logares, R. *et al.* Recent evolutionary diversification of a protist lineage. *Environmental Microbiology* **10**, 1231–1243 (2008).

8 Gottschling, M. & Söhner, S. An updated list of generic names in the Thoracosphaeraceae. *Microorganisms* **1**, 122–136 (2013).

9 Yamaguchi, A., Hoppenrath, M., Pospelova, V., Horiguchi, T. & Leander, B. S. Molecular phylogeny of the marine sand-dwelling dinoflagellate *Herdmania litoralis* and an emended description of the closely related planktonic genus *Archaeperidinium* Jörgensen. *European Journal of Phycology* **46**, 98–112 (2011).

10 Coats, D. W. *et al.* Prevalence and phylogeny of parasitic dinoflagellates (genus *Blastodinium*) infecting copepods in the Gulf of California. *CICIMAR Oceánides* **23**, 67–77 (2008).

11 Horiguchi, T. & Takano, Y. Serial replacement of a diatom endosymbiont in the marine dinoflagellate *Peridinium quinquecorne* (Peridiniales, Dinophyceae). *Phycological Research* **54**, 193–200 (2006).

12 Luo, Z. *et al.* Morphology, ultrastructure and molecular phylogeny of cyst-producing *Caladoa arcachonensis* gen. et sp. nov. (Peridiniales, Dinophyceae) from France and Indonesia. *European Journal of Phycology* **54**, 235–248 (2019).

13 D´Onofrio, G., Marino, D., Bianco, L., Busico, E. & Montresor, M. Toward an assessment on the taxonomy of dinoflagellates that produce calcareous cysts (Calciodinelloideae, Dinophyceae): A morphological and molecular approach. *Journal of Phycology* **35**, 1063–1078 (1999).

14 Montresor, M., Sgrosso, S., Procaccini, G. & Kooistra, W. H. C. F. Intraspecific diversity in *Scrippsiella trochoidea* (Dinophyceae): Evidence for cryptic species. *Phycologia* **42**, 56–70 (2003).

15 Gottschling, M. & Plötner, J. Secondary structure models of the nuclear Internal Transcribed Spacer regions and 5.8S rRNA in Calciodinelloideae (Peridiniaceae) and other dinoflagellates. *Nucleic Acids Research* **32**, 307–315 (2004).

16 Zinßmeister, C. *et al.* Same but different: Two novel bicarinate species of extant calcareous dinophytes (Thoracosphaeraceae, Peridiniales) from the Mediterranean Sea. *Journal of Phycology* **48**, 1107–1118 (2012).

17 Yamada, N., Sakai, H., Onuma, R., Kroth, P. G. & Horiguchi, T. Five non-motile dinotom dinoflagellates of the genus *Dinothrix*. *Frontiers in Plant Science* **11**, 591050. (2020).

18 Yamada, N., Tanaka, A. & Horiguchi, T. Pigment compositions are linked to the habitat types in dinoflagellates. *Journal of Plant Research* **128**, 923–932 (2015).

19 Yamada, N., Sym, S. D. & Horiguchi, T. Identification of highly divergent diatom-derived chloroplasts in dinoflagellates, including a description of *Durinskia kwazulunatalensis* sp. nov. (Peridiniales, Dinophyceae). *Molecular Biology and Evolution* **34**, 1335–1351 (2017).

20 Tamura, M., Shimada, S. & Horiguchi, T. *Galeidinium rugatum* gen. et sp. nov. (Dinophyceae), a new coccoid dinoflagellate with a diatom endosymbiont. *Journal of Phycology* **41**, 658–671 (2005).

21 Coats, D. W., Kim, S., Bachvaroff, T. R., Handy, S. M. & Delwiche, C. F. *Tintinnophagus acutus* n. g., n. sp. (Phylum Dinoflagellata), an ectoparasite of the ciliate *Tintinnopsis cylindrica* Daday 1887, and its relationship to *Duboscquodinium collini* Grassé 1952. *Journal of Eukaryotic Microbiology* **57**, 468–482 (2010).

22 Saburova, M., Chomérat, N. & Hoppenrath, M. Morphology and SSU rDNA phylogeny of *Durinskia agilis* (Kofoid & Swezy) comb. nov. (Peridiniales, Dinophyceae), a thecate, marine, sand-dwelling dinoflagellate formerly classified within *Gymnodinium*. *Phycologia* **51**, 287–302 (2012).

23 Inagaki, Y., Dacks, J. B., Doolittle, W. F., Watanabe, K. I. & Ohama, T . Evolutionary relationship between dinoflagellates bearing obligate diatom endosymbionts: Insight into tertiary endosymbiosis. *International Journal of Systematic and Evolutionary Microbiology* **50**, 2075–2081 (2000).

24 Imanian, B., Pombert, J.-F. & Keeling, P. J. The complete plastid genomes of the two 'dinotoms' *Durinskia baltica* and *Kryptoperidinium foliaceum*. *PLoS One* **5**, e10711 (2011).

25 Kim, S., Yoon, J. & Park, M. G. 서해안 동호 사질 조간대에 서식하는 저서성 와편모류의 출현양상 및 분자계통학적 특성. *The Sea, Journal of the Korean Society of Oceanography* **20**, 141–150 (2015).

26 Chesnick, J. M., Kooistra, W. H. C. F., Wellbrock, U. & Medlin, L. K. Ribosomal RNA analysis indicates a benthic pennate diatom ancestry for the endosymbionts of the dinoflagellates *Peridinium foliaceum* and *Peridinium balticum* (Pyrrhophyta). *Journal of Eukaryotic Microbiology* **44**, 314–320 (1997).

27 Yamada, N. *et al.* Discovery of a kleptoplastic 'dinotom' dinoflagellate and the unique nuclear dynamics of converting kleptoplastids to permanent plastids. *Scientific Reports* **9**, 10474 (2019).

28 Kretschmann, J., Žerdoner Čalasan, A. & Gottschling, M. Molecular phylogenetics of dinophytes harbouring diatoms as endosymbionts (Kryptoperidiniaceae, Peridiniales), with evolutionary interpretations and a focus on the identity of *Durinskia oculata* from Prague. *Molecular Phylogenetics and Evolution* **118**, 392–402 (2018).

29 Žerdoner Čalasan, A., Kretschmann, J. & Gottschling, M. Absence of co-phylogeny indicates repeated diatom capture in dinophytes hosting a tertiary endosymbiont. *Organisms Diversity & Evolution* **18**, 29–38 (2018).

30 Zhang, Q., Liu, G.-X. & Hu, Z.-Y. *Durinskia baltica* (Dinophyceae), a newly recorded species and genus from China, and its systematics. *Journal of Systematics and Evolution* **49**, 476–485 (2011).

31 Gottschling, M., Renner, S. S., Meier, K. J. S., Willems, H. & Keupp, H. Timing deep divergence events in calcareous dinoflagellates. *Journal of Phycology* **44**, 429–438 (2008).

32 Gottschling, M. *et al.* Delimitation of the Thoracosphaeraceae (Dinophyceae), including the calcareous dinoflagellates, based on large amounts of ribosomal RNA sequence data. *Protist* **163**, 15–24 (2012).

33 Moestrup, Ø. & Daugbjerg, N. in *Unravelling the algae, the past, present, and future of algal systematics Systematics Association Special Volume No. 75* (eds J. Brodie & J. Lewis) 215–230 (CRC Press, 2007).

34 Yoshida, T. *et al.* Sequence analysis of 5.8S rDNA and the Internal Transcribed Spacer region in dinoflagellate *Heterocapsa* species (Dinophyceae) and development of selective PCR primers for the bivalve killer *Heterocapsa circularisquama*. *Microbes and Environments* **18**, 216–222 (2003).

35 Hansen, G. & Daugbjerg, N. Ultrastructure of *Gyrodinium spirale*, the type species of *Gyrodinium* (Dinophyceae), including a phylogeny of *G. dominans*, *G. rubrum* and *G. spirale* deduced from partial LSU rDNA sequences. *Protist* **155**, 271–294 (2004).
36 Stern, R. F. *et al.* Evaluating the ribosomal Internal Transcribed Spacer (ITS) as a candidate dinoflagellate barcode marker. *PLoS One* **7**, e42780 (2012).
37 Keeling, P. J. *et al.* The Marine Microbial Eukaryote Transcriptome Sequencing Project (MMETSP): Illuminating the functional diversity of eukaryotic life in the oceans through transcriptome sequencing. *PLoS Biology* **12**, e1001889 (2014).
38 Tillmann, U., Hoppenrath, M., Gottschling, M., Kusber, W.-H. & Elbrächter, M. Plate pattern clarification of the marine dinophyte *Heterocapsa triquetra sensu* Stein (Dinophyceae) collected at the Kiel Fjord (Germany). *Journal of Phycology* **53**, 1305–1324 (2017).
39 Potvin, É. *et al.* *Islandinium minutum* subsp. *barbatum* subsp. nov. (Dinoflagellata), a new organic-walled dinoflagellate cyst from the Western Arctic: Morphology, phylogenetic position based on SSU rDNA and LSU rDNA, and distribution. *Journal of Eukaryotic Microbiology* **65**, 750–772 (2018).
40 Kempton, J. *et al.* *Kryptoperidinium foliaceum* blooms in South Carolina: A multi-analytical approach to identification. *Harmful Algae* **1**, 383–392 (2002).
41 Yoon, H. S., Hackett, J. D. & Bhattacharya, D. A single origin of the peridinin- and fucoxanthin-containg plastids in dinoflagellates through tertiary endosymbiosis. *Proceedings of the National Academy of Sciences of the USA* **99**, 11724–11729 (2002).
42 Yoon, H. S. *et al.* Tertiary endosymbiosis driven genome evolution in dinoflagellate algae. *Molecular Biology and Evolution* **22**, 1299–1308 (2005).
43 Imanian, B., Pombert, J. F. & Keeling, P. J. The complete plastid genomes of the two 'dinotoms' *Durinskia baltica* and *Kryptoperidinium foliaceum*. *PLoS One* **5**, e10711 (2010).
44 Leblond, J. D. *et al.* A data mining approach to dinoflagellate clustering according to sterol composition: Correlations with evolutionary history. *International Journal of Data Mining and Bioinformatics* **4**, 431–451 (2010).
45 Saldarriaga Echavarría, J. F., Taylor, F. J. R., Keeling, P. J. & Cavalier-Smith, T . Dinoflagellate nuclear SSU rRNA phylogeny suggests multiple plastid losses and replacements. *Journal of Molecular Evolution* **53**, 204–213 (2001).
46 Chesnick, J. M., Morden, C. W. & Schmieg, A. M. Identity of the endosymbiont of *Peridinium foliaceum* (Pyrrophyta): Analysis of the *rbcLS* operon. *Journal of Phycology* **32**, 850–857 (1996).
47 黄永梅 *et al.* 湛江沿海海洋微藻及其多糖、脂类和蛋白藻株多样性研究. *水生生物学报* **41**, 1080–1090 (2017).
48 Lewis, J., Taylor, J. D., Neale, K. & Leroy, S. A. G. Expanding known dinoflagellate distributions: Investigations of slurry cultures from Caspian Sea sediment. *Botanica Marina* **61**, 21–31 (2018).
49 Satta, C. T. *et al.* Ecological, morphological and molecular characterization of *Kryptoperidinium* sp. (Dinophyceae) from two Mediterranean coastal shallow lagoons. *Harmful Algae* **97**, 101855 (2020).
50 Richardson, E., Dorrell, R. G. & Howe, C. J. Genome-wide transcript profiling reveals the coevolution of plastid gene sequences and transcript processing pathways in the fucoxanthin dinoflagellate *Karlodinium veneficum*. *Molecular Biology and Evolution* **31**, 2376–2386, doi:10.1093/molbev/msu189 (2014).
51 Logares, R., Shalchian-Tabrizi, K., Boltovskoy, A. & Rengefors, K. Extensive dinoflagellate phylogenies indicate infrequent marine-freshwater transitions. *Molecular Phylogenetics and Evolution* **45**, 887–903 (2007).
52 Hansen, G., Daugbjerg, N. & Henriksen, P. *Baldinia anauniensis* gen. et sp. nov.: A ‘new’ dinoflagellate from Lake Tovel, N. Italy. *Phycologia* **46**, 86–108 (2007).
53 Hansen, G., Daugbjerg, N. & Moestrup, Ø. The rainwater rock-pool dinoflagellate *Nottbeckia ochracea* gen. et comb. nov (syn.: *Hemidinium ochraceum*) - A fine-structural and molecular study with emphasis on the motile stage. *Protist* **169**, 280–306 (2018).

54 Kretschmann, J., Žerdoner Čalasan, A., Kusber, W.-H. & Gottschling, M. Still curling after all these years: *Glenodinium apiculatum* Ehrenb. (Peridiniales, Dinophyceae) repeatedly found at its type locality in Berlin (Germany). *Systematics and Biodiversity* **16**, 200–209 (2018).
55 Kretschmann, J., Owsianny, P. M., Žerdoner Čalasan, A. & Gottschling, M. The hot spot in a cold environment: Puzzling *Parvodinium* (Peridiniopsidaceae, Peridiniales) from the Polish Tatra Mountains. *Protist* **169**, 206–230 (2018).
56 Logares, R., Boltovskoy, A., Bensch, S., Laybourn-Parry, J. & Rengefors, K. Genetic diversity patterns in five protist species occurring in lakes. *Protist* **160**, 301–317 (2009).
57 Craveiro, S. C., Calado, A. J., Daugbjerg, N. & Moestrup, Ø. Ultrastructure and LSU rDNA-based revision of *Peridinium* group Palatinum (Dinophyceae) with the description of *Palatinus* gen. nov. *Journal of Phycology* **45**, 1175–1194 (2009).
58 Matsuoka, K. & Kawami, H. in *Biological and geological perspectives of dinoflagellates Micropalaeontological Society special publications* (eds J. Lewis, F. Marret, & L. Bradley) 275-284 (Micropalaeontological Society by the Geological Society, 2013).
59 Ki, J.-S., Park, M.-H. & Han, M.-S. Discriminative power of nuclear rDNA sequences for the DNA taxonomy of the dinoflagellate genus *Peridinium* (Dinophyceae). *Journal of Phycology* **47**, 426–435 (2011).
60 Ki, J.-S. & Han, M.-S. Rapid molecular identification of the harmful freshwater dinoflagellate *Peridinium* in various life stages using genus-specific single-cell PCR. *Journal of Applied Phycology* **19**, 467–470 (2007).
61 Gottschling, M., Kretschmann, J. & Žerdoner Čalasan, A. Description of Peridiniopsidaceae, fam. nov. (Peridiniales, Dinophyceae). *Phytotaxa* **299**, 293–296 (2017).
62 Takano, Y. & Horiguchi, T. Acquiring scanning electron microscopical, light microscopical and multiple gene sequence data from a single dinoflagellate cell. *Journal of Phycology* **42**, 251–256 (2006).
63 Saito, K., Drgon, T., Robledo, J. A. F., Krupatkina, D. N. & Vasta, G. R. Characterization of the rRNA locus of *Pfiesteria piscicida* and development of standard and quantitative PCR-based detection assays targeted to the nontranscribed spacer. *Applied and Environmental Microbiology* **68**, 5394–5407 (2002).
64 Gu, H. *et al.* Waking the dead: Morphological and molecular characterization of extant †*Posoniella tricarinelloides* (Thoracosphaeraceae, Dinophyceae). *Protist* **164**, 583–597 (2013).
65 Gribble, K. E. & Anderson, D. M. High intraindividual, intraspecific, and interspecific variability in large-subunit ribosomal DNA in the heterotrophic dinoflagellates *Protoperidinium*, *Diplopsalis*, and *Preperidinium* (Dinophyceae). *Phycologia* **46**, 315–324 (2007).
66 Yamaguchi, A., Kawamura, H. & Horiguchi, T. The phylogenetic position of an unusual *Protoperidinium* species, *P. bipes* (Peridiniales, Dinophyceae), based on small and large subunit ribosomal RNA gene sequences. *Phycologia* **46**, 270–276 (2007).
67 Yamaguchi, A. & Horiguchi, T. Molecular phylogenetic study of the heterotrophic dinoflagellate genus *Protoperidinium* (Dinophyceae) inferred from small subunit rRNA gene sequences. *Phycological Research* **53**, 30–42 (2005).
68 Yamaguchi, A., Kawamura, H. & Horiguchi, T. A further phylogenetic study of the heterotrophic dinoflagellate genus, *Protoperidinium* (Dinophyceae) based on small and large subunit ribosomal RNA gene sequences. *Phycological Research* **54**, 317–329 (2006).
69 Žerdoner Čalasan, A., Kretschmann, J. & Gottschling, M. They are young, and they are many: Dating freshwater lineages in unicellular dinophytes. *Environmental Microbiology* **21**, 4125–4135 (2019).
70 Craveiro, S. C., Pandeirada, M. S., Daugbjerg, N., Moestrup, Ø. & Calado, A. J. Ultrastructure and phylogeny of *Theleodinium calcisporum* gen. et sp. nov., a freshwater dinoflagellate that produces calcareous cysts. *Phycologia* **52**, 488–507 (2013).

71 You, X., Luo, Z., Su, Y., Gu, L. & Gu, H. *Peridiniopsis jiulongensis*, a new freshwater dinoflagellate with a diatom endosymbiont from China. *Nova Hedwigia* **101**, 313–326 (2015).

72 Gottschling, M., Žerdoner Čalasan, A., Kretschmann, J. & Gu, H. Two new generic names for dinophytes harbouring a diatom as an endosymbiont, *Blixaea* and *Unruhdinium* (Kryptoperidiniaceae, Peridiniales). *Phytotaxa* **306**, 296–300 (2017).

73 Zhang, Q., Liu, G.-X. & Hu, Z.-Y. Description of a new freshwater bloom-forming dinoflagellate with a diatom endosymbiont, *Peridiniopsis minima* sp. nov. (Peridiniales, Dinophyceae) from China. *Algological Studies* **145**, 119–133 (2014).

74 Zhang, Q., Liu, G.-X. & Hu, Z.-Y. Morphological differences and molecular phylogeny of freshwater blooming species, *Peridiniopsis* spp. (Dinophyceae) from China. *European Journal of Protistology* **47**, 149–160 (2011).

75 Takano, Y., Hansen, G., Fujita, D. & Horiguchi, T. Serial replacement of diatom endosymbionts in two freshwater dinoflagellates, *Peridiniopsis* spp. (Peridiniales, Dinophyceae). *Phycologia* **47**, 41–53 (2008).

76 Luo, Z. *et al.* Morpho-molecular diversity and phylogeny of *Bysmatrum* (Dinophyceae) from the South China Sea and France. *European Journal of Phycology* **53**, 318–335 (2018).

77 Gast, R. J. & Caron, D. A. Molecular phylogeny of symbiotic dinoflagellates from planktonic Foraminifera and Radiolaria. *Molecular Biology and Evolution* **13**, 1192–1197 (1996).

78 Gottschling, M. & McLean, T. I. New home for tiny symbionts: Dinophytes determined as *Zooxanthella* are Peridiniales and distantly related to *Symbiodinium*. *Molecular Phylogenetics and Evolution* **67**, 217–222 (2013).

79 Scholin, C. A., Herzog, M., Sogin, M. & Anderson, D. M. Identification of group- and strain-specific genetic markers for globally distributed *Alexandrium* (Dinophyceae). II. Sequence analysis of a fragment of the LSU rRNA gene. *Journal of Phycology* **30**, 999–1011 (1994).

80 Daugbjerg, N., Hansen, G., Larsen, J. & Moestrup, Ø. Phylogeny of some of the major genera of dinoflagellates based on ultrastructure and partial LSU rDNA sequence data, including the erection of three new genera of unarmoured dinoflagellates. *Phycologia* **39**, 302–317 (2000).

81 White, T. J., Bruns, T., Lee, S. & Taylor, F. J. R. in *PCR protocols: A guide to methods and applications* (eds M. A. Innis, D. H. Gelfand, J. J. Sninsky, & T. J. White) 315–322 (Academic Press, 1990).

82 Hsiao, C., Chatterton, N. J., Asay, K. H. & Jensen, K. B. Phylogenetic relationships of the monogenomic species of the wheat tribe, Triticeae (Poaceae), inferred from nuclear rDNA (internal transcribed spacer) sequences. *Genome announcements* **38**, 211–223 (1995).

83 Adachi, M., Sako, Y. & Ishida, Y. Analysis of *Alexandrium* (Dinophyceae) species using sequences of the 5.8S ribosomal DNA and internal transcribed spacer regions. *Journal of Phycology* **32**, 424–432 (1996).

84 Elwood, H. J., Olsen, G. J. & Sogin, M. L. The small-subunit ribosomal RNA gene sequences from the hypotrichous ciliates *Oxytricha nova* and *Stylonychia pustulata*. *Molecular Biology and Evolution* **2**, 399–410 (1985).

85 Medlin, L. K., Elwood, H. J., Stickel, S. & Sogin, M. L. The characterization of enzymatically amplified eukaryotic 16S-like rRna-coding regions. *Gene* **71**, 491–499 (1988).

86 Friedl, T. Evolution of the polyphyletic genus *Pleurastrum* (Chlorophyta): Inferences from nuclear-encoded ribosomal DNA sequences and motile cell ultrastructure. *Phycologia* **35**, 456–469 (1996).

87 Montresor, M., John, U., Beran, A. & Medlin, L. K. *Alexandrium tamutum* sp. nov. (Dinophyceae): A new nontoxic species in the genus *Alexandrium*. *Journal of Phycology* **40**, 398–411 (2004).

88 Giovannoni, S. J., DeLong, E. F., Olsen, G. J. & Pace, N. R. Phylogenetic group-specific oligodeoxynucleotide probes for identification of single microbial cells. *Journal of Bacteriology* **170**, 720–726 (1988).
