# Supplementary figures and images for: Spatial fragmentation in the distribution of diatom endosymbionts from the taxonomically clarified dinophyte Kryptoperidinium triquetrum (= Kryptoperidinium foliaceum, Peridiniales)
Source: Sci Rep. 2023 May 26;13:8593. doi: 10.1038/s41598-023-32949-y (PMC10219988; doi:10.1038/s41598-023-32949-y)

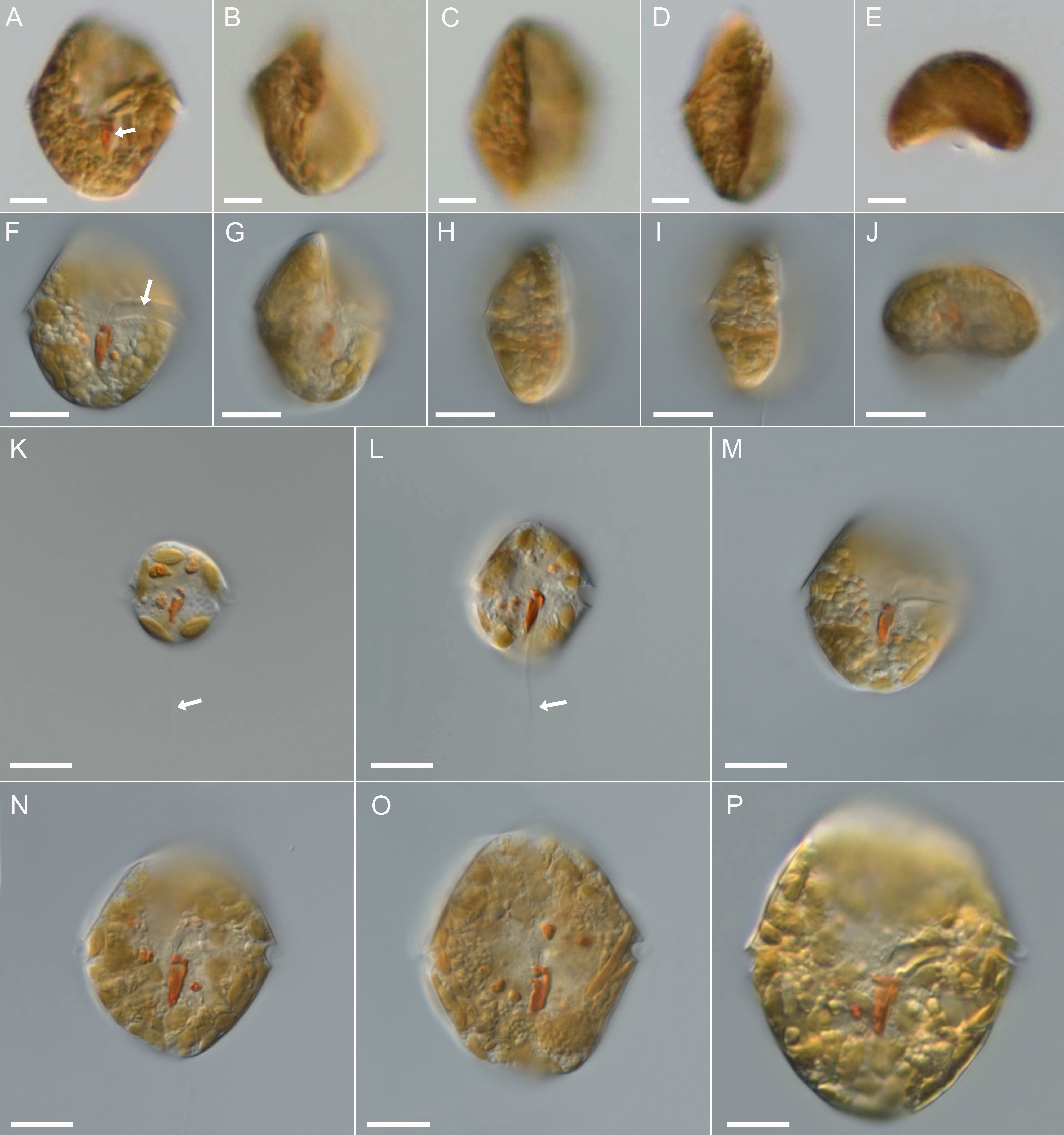

Supplement: Supplementary file 5 — Supplementary Figure 1. [file 41598_2023_32949_MOESM5_ESM.jpg]

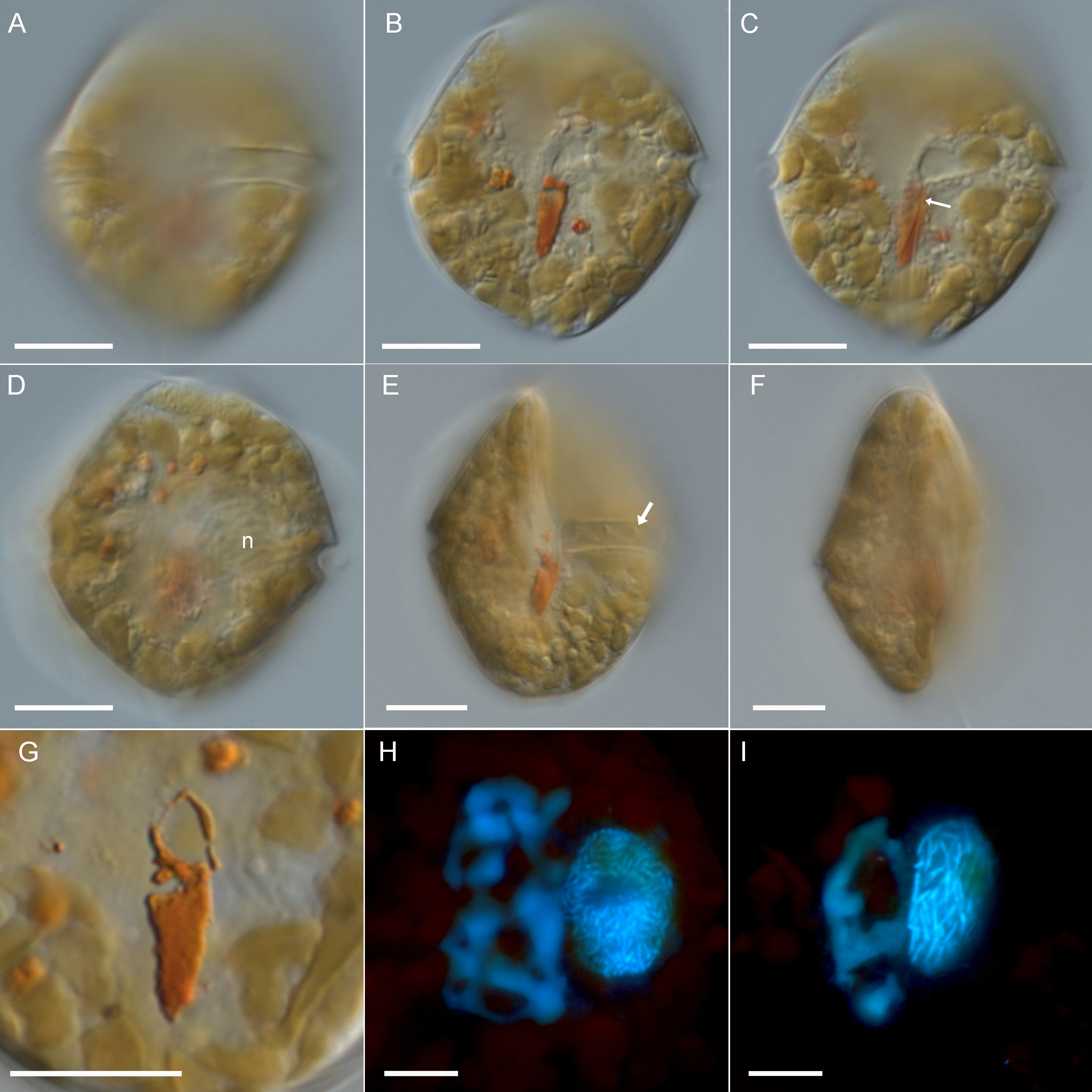

Supplement: Supplementary file 6 — Supplementary Figure 2. [file 41598_2023_32949_MOESM6_ESM.jpg]

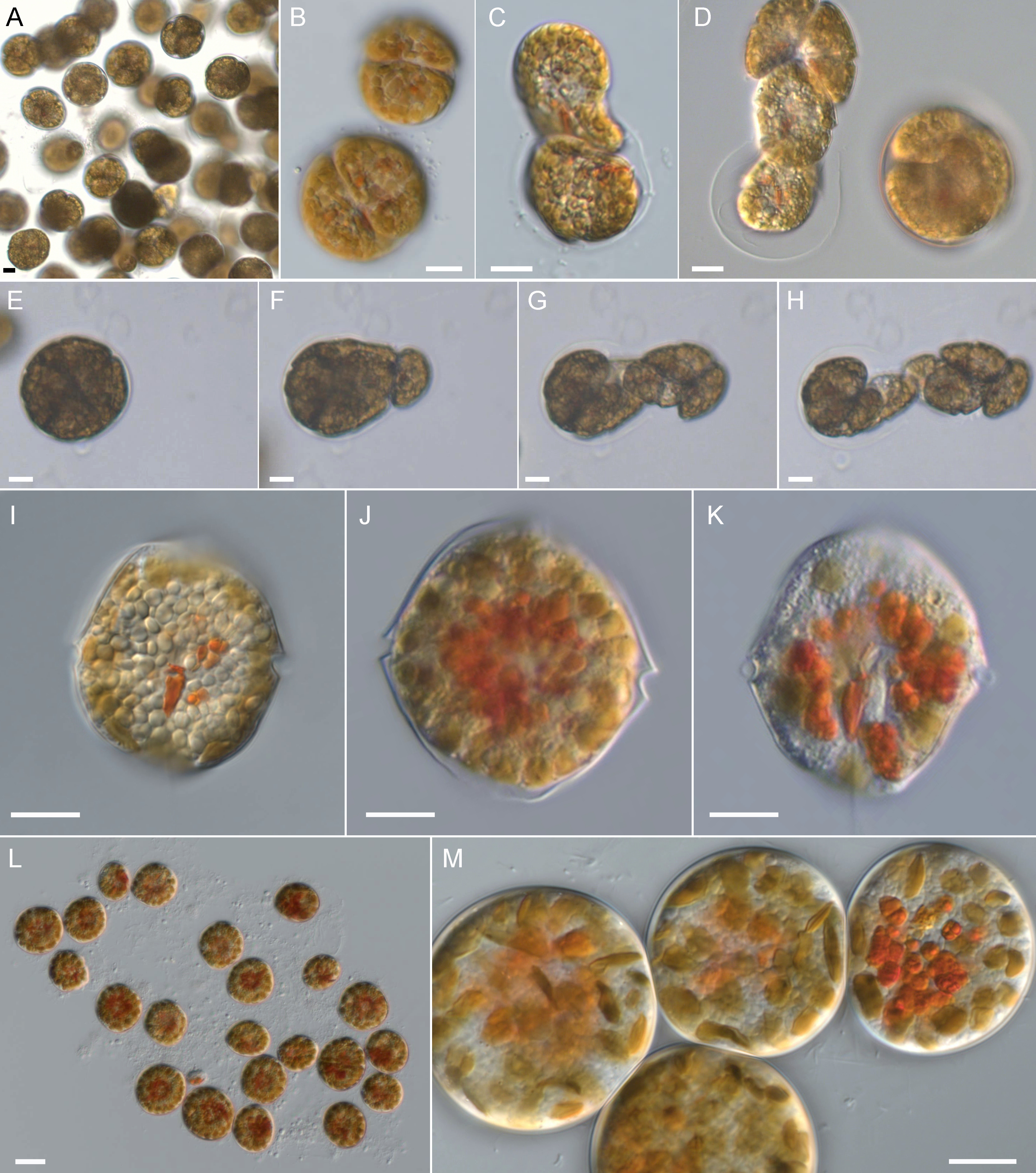

Supplement: Supplementary file 7 — Supplementary Figure 3. [file 41598_2023_32949_MOESM7_ESM.jpg]

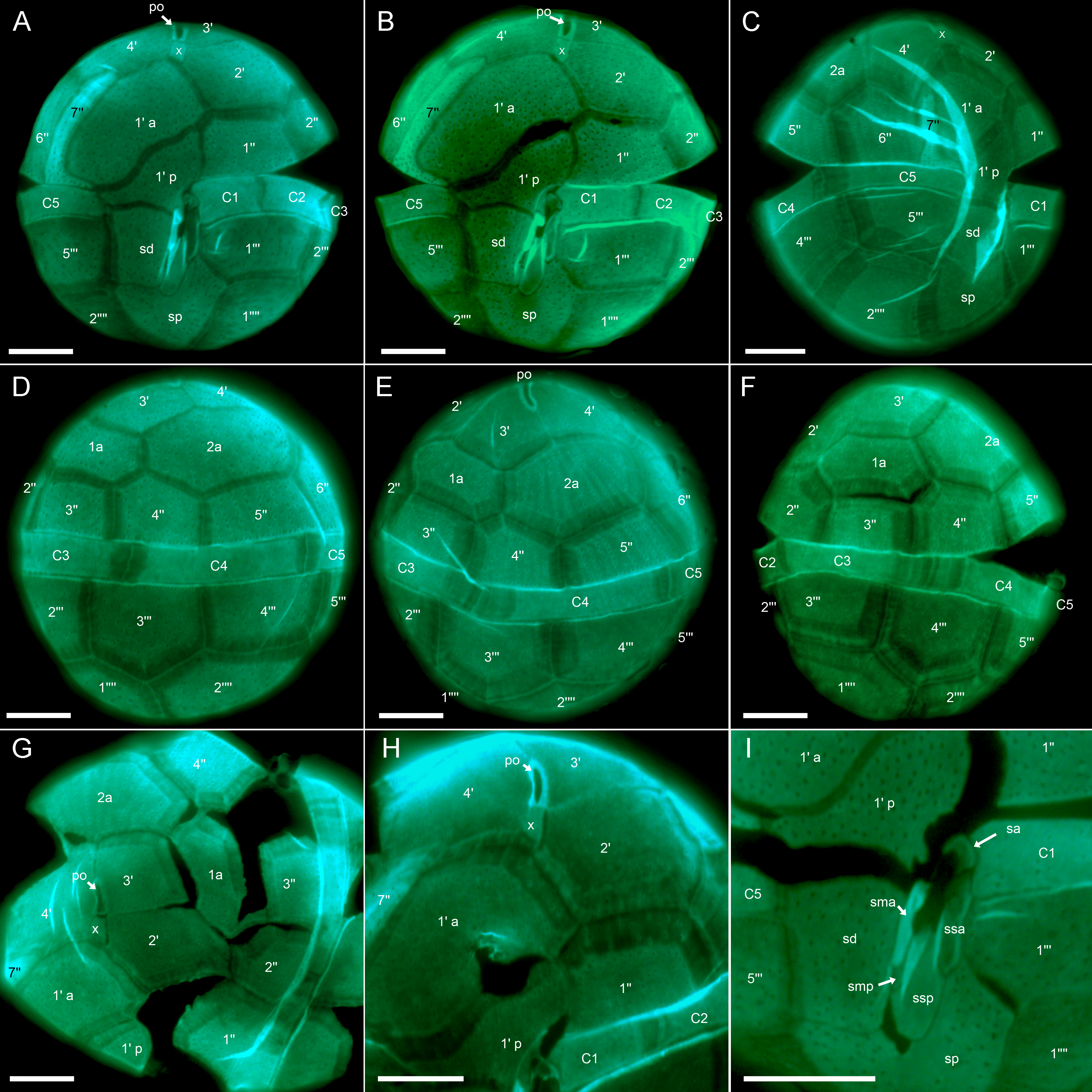

Supplement: Supplementary file 8 — Supplementary Figure 4. [file 41598_2023_32949_MOESM8_ESM.jpg]

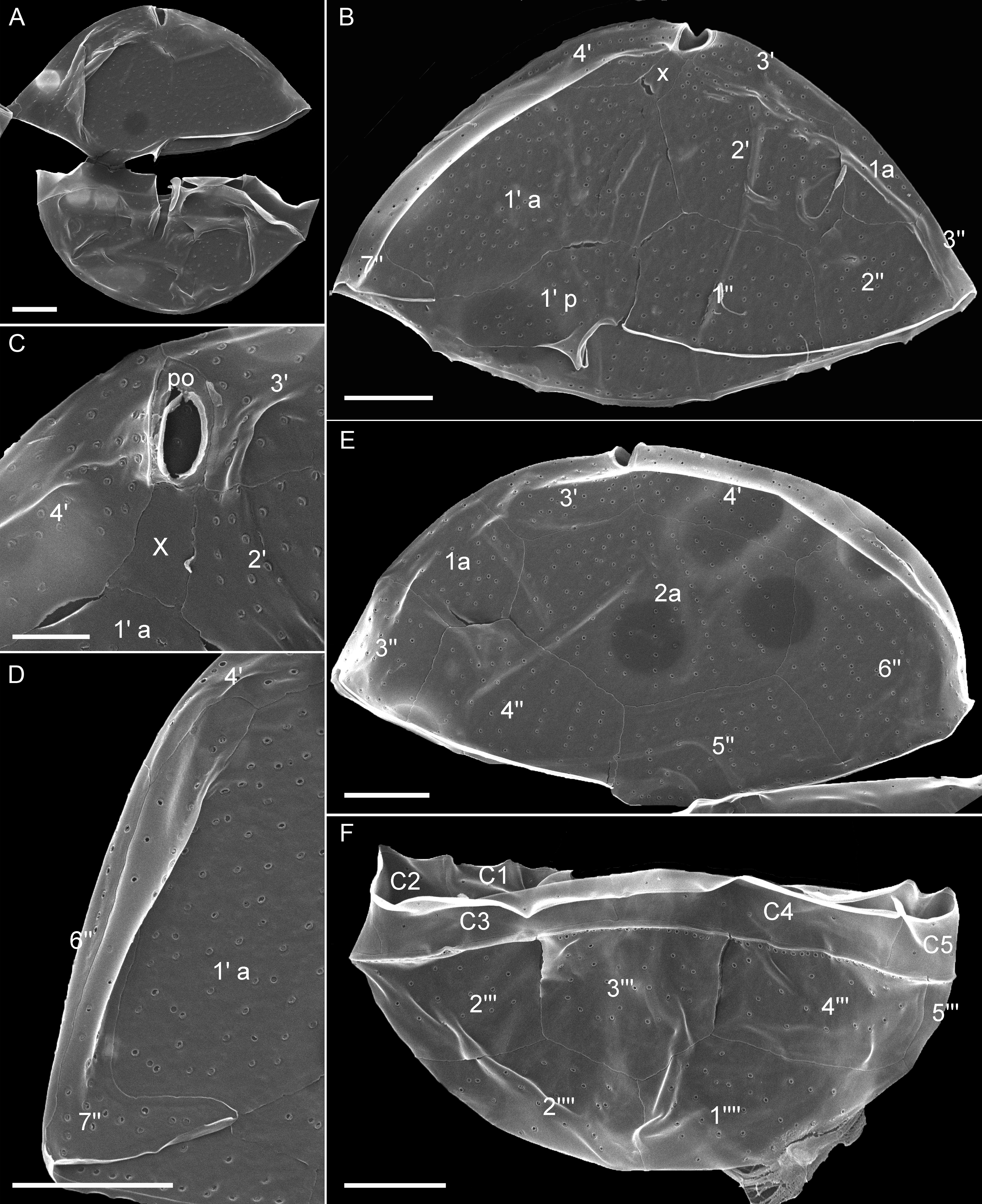

Supplement: Supplementary file 9 — Supplementary Figure 5. [file 41598_2023_32949_MOESM9_ESM.jpg]

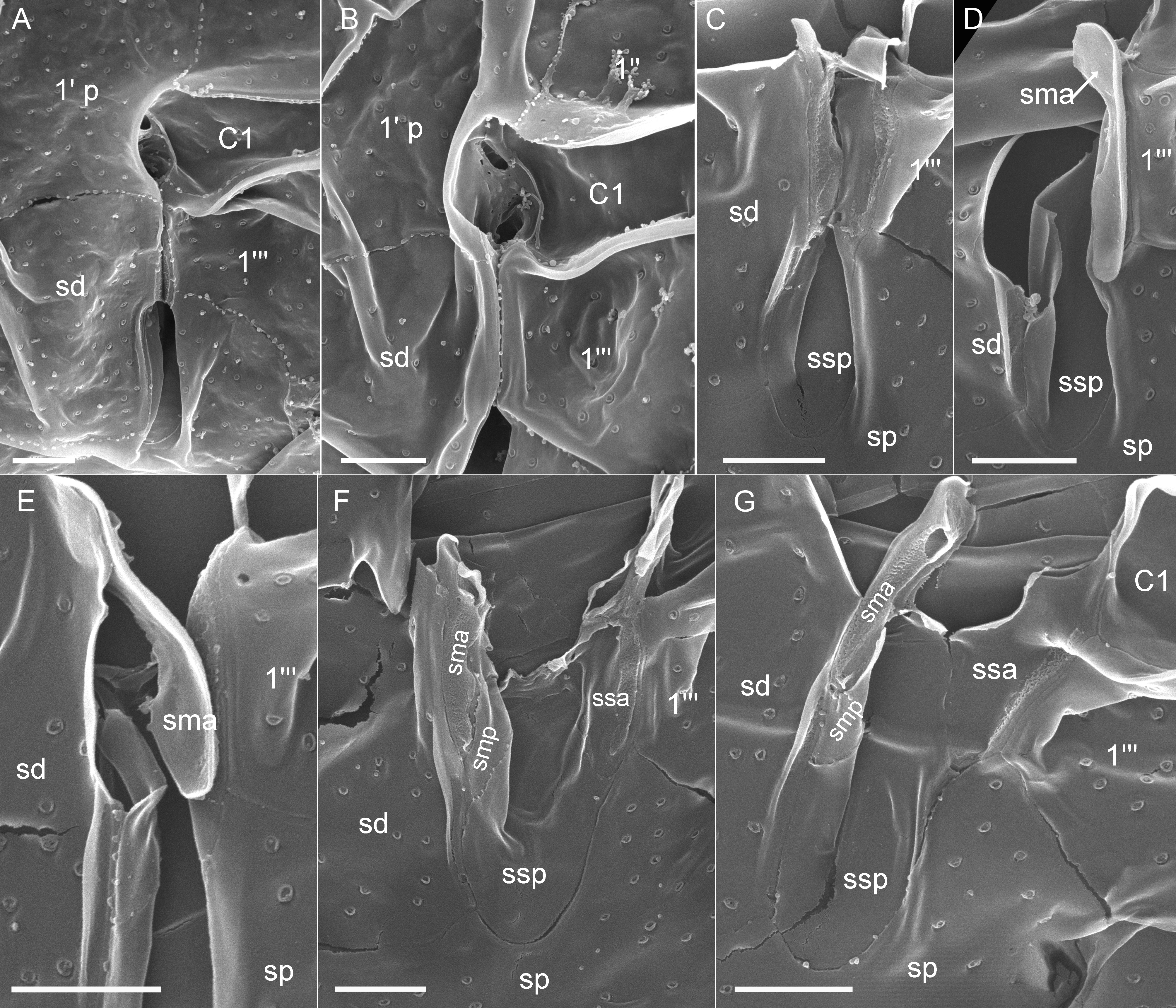

Supplement: Supplementary file 10 — Supplementary Figure 6. [file 41598_2023_32949_MOESM10_ESM.jpg]

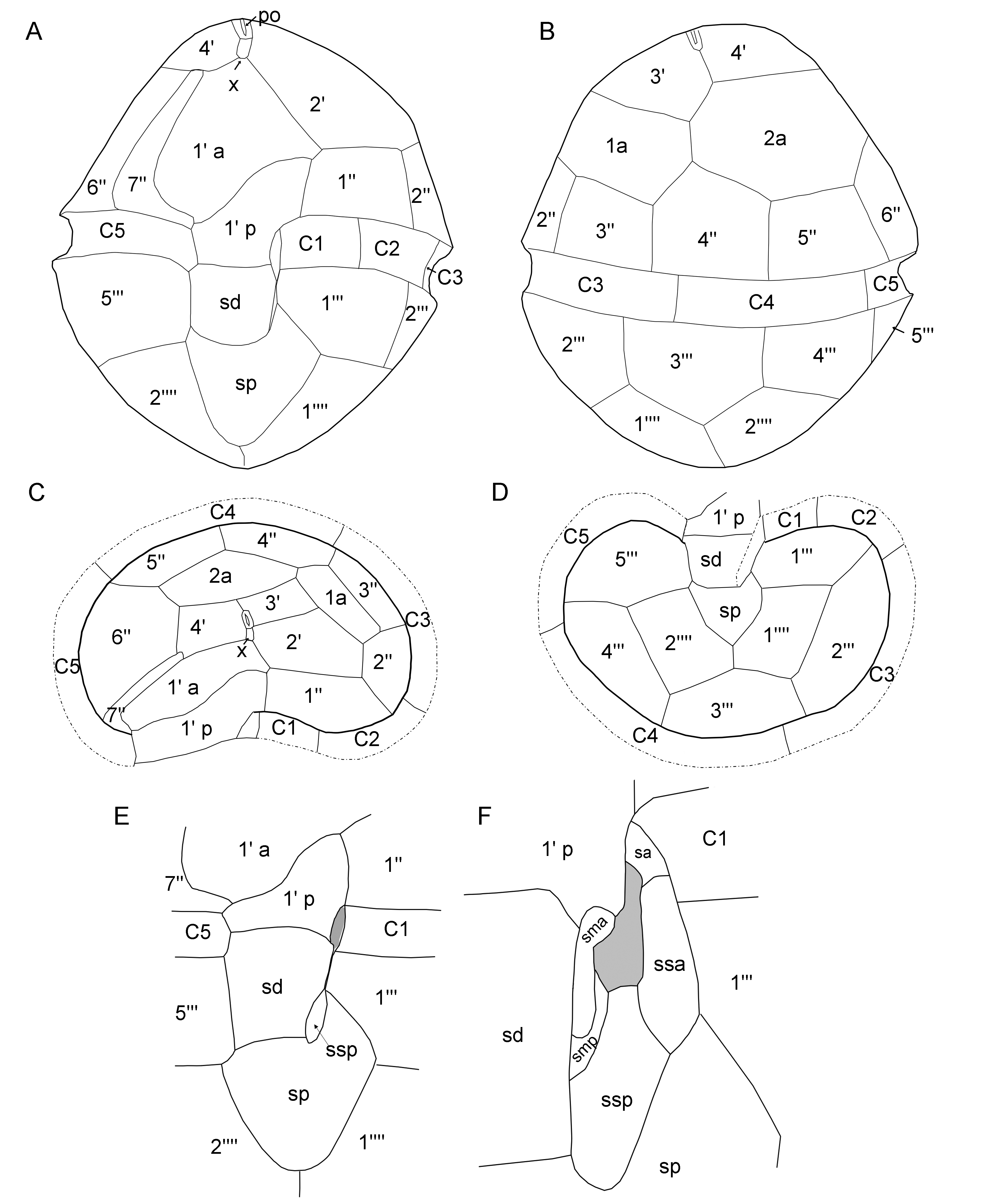

Supplement: Supplementary file 11 — Supplementary Figure 7. [file 41598_2023_32949_MOESM11_ESM.jpg]

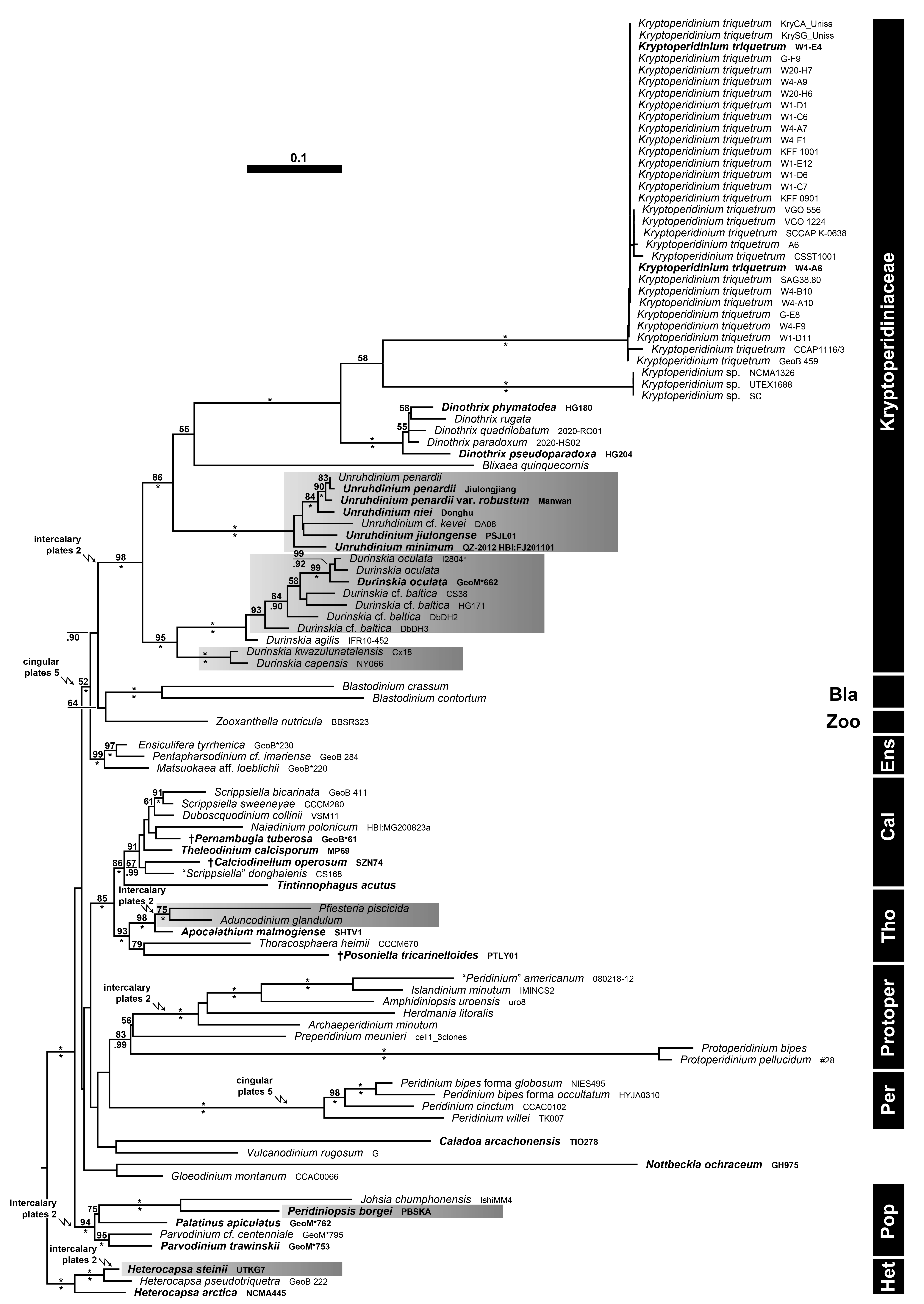

Supplement: Supplementary file 12 — Supplementary Figure 8. [file 41598_2023_32949_MOESM12_ESM.jpg]

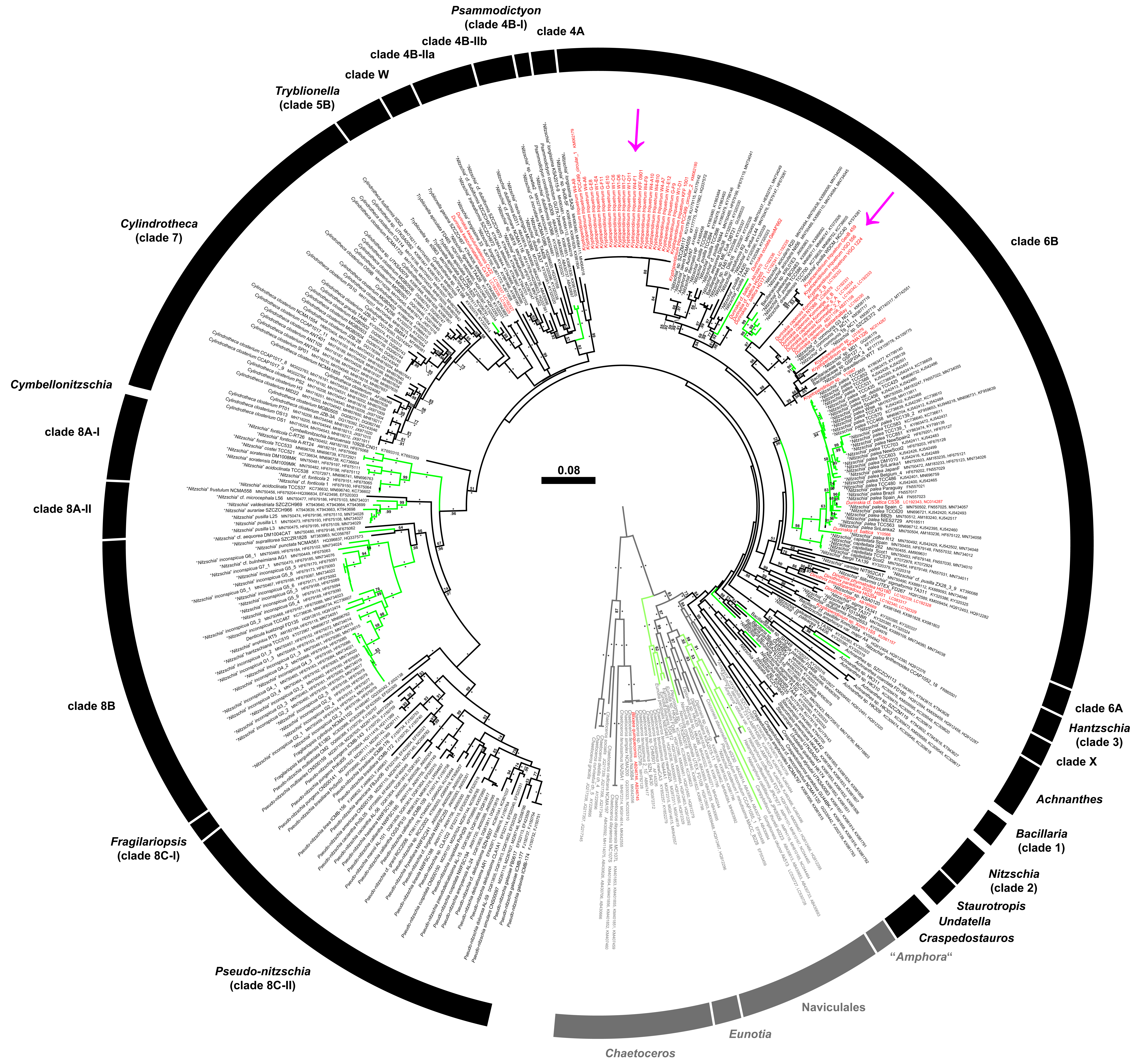

Supplement: Supplementary file 13 — Supplementary Figure 9. [file 41598_2023_32949_MOESM13_ESM.jpg]

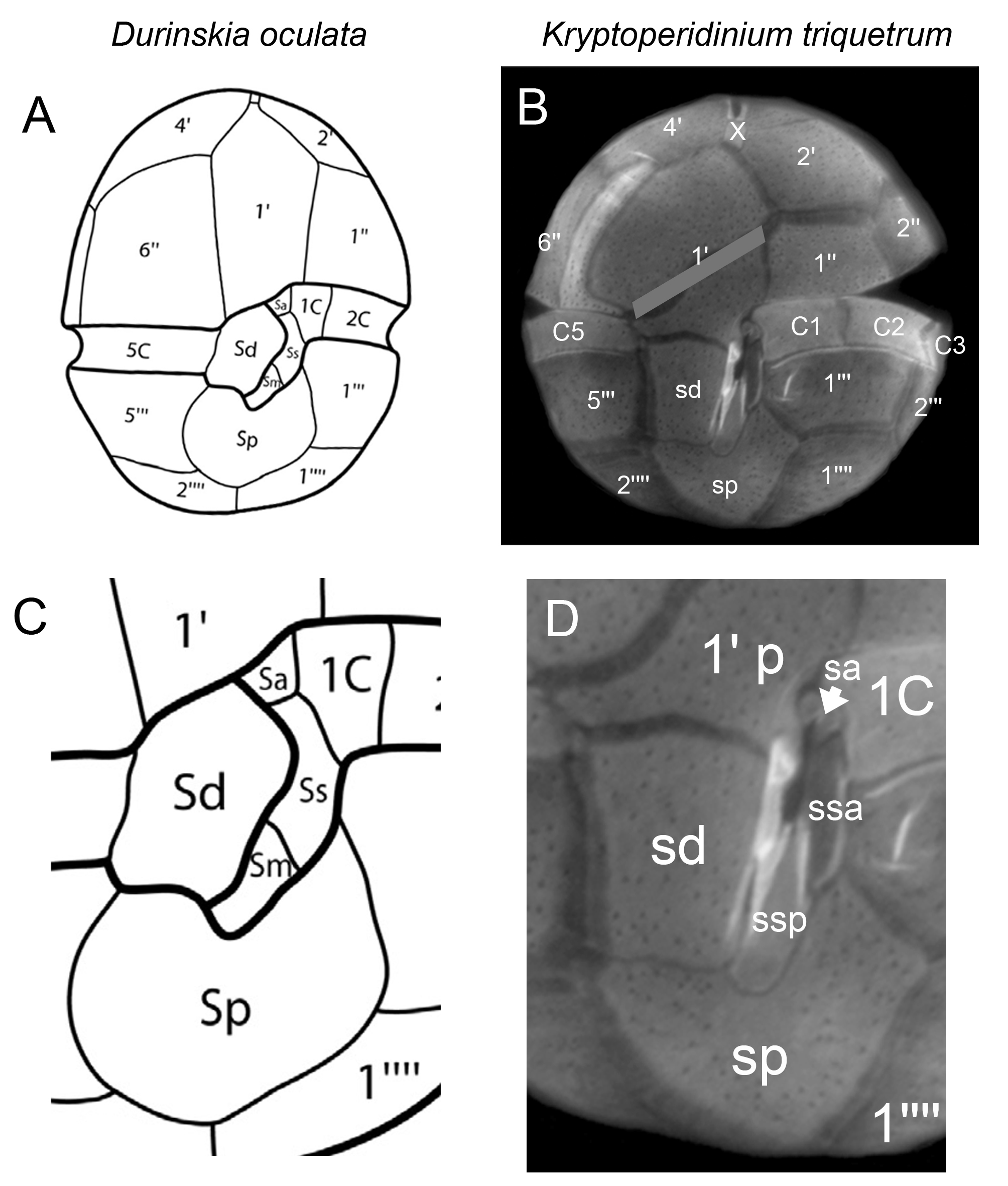

Supplement: Supplementary file 14 — Supplementary Figure 10. [file 41598_2023_32949_MOESM14_ESM.jpg]
